# Actively implementing an evidence-based feeding guideline for critically ill patients (NEED): a multicenter, cluster-randomized, controlled trial

**DOI:** 10.1186/s13054-022-03921-5

**Published:** 2022-02-16

**Authors:** Lu Ke, Jiajia Lin, Gordon S. Doig, Arthur R. H. van Zanten, Yang Wang, Juan Xing, Zhongheng Zhang, Tao Chen, Lixin Zhou, Dongpo Jiang, Qindong Shi, Jiandong Lin, Jun Liu, Aibin Cheng, Yafeng Liang, Peiyang Gao, Junli Sun, Wenming Liu, Zhenyu Yang, Rumin Zhang, Wei Xing, An Zhang, Zhigang Zhou, Tingfa Zhou, Yang Liu, Fei Tong, Qiuhui Wang, Aijun Pan, Xiaobo Huang, Chuming Fan, Weihua Lu, Dongwu Shi, Lei Wang, Wei Li, Liming Gu, Yingguang Xie, Rongqing Sun, Feng Guo, Lin Han, Lihua Zhou, Xiangde Zheng, Feng Shan, Jianbo Liu, Yuhang Ai, Yan Qu, Liandi Li, Hailing Li, Zhiguo Pan, Donglin Xu, Zhiqiang Zou, Yan Gao, Chunli Yang, Qiuye Kou, Xijing Zhang, Jinglan Wu, Chuanyun Qian, Weixing Zhang, Minjie Zhang, Yuan Zong, Bingyu Qin, Fusen Zhang, Zhe Zhai, Yun Sun, Ping Chang, Bo Yu, Min Yu, Shiying Yuan, Yijun Deng, Liyun Zhao, Bin Zang, Yuanfei Li, Fachun Zhou, Xiaomei Chen, Min Shao, Weidong Wu, Ming Wu, Zhaohui Zhang, Yimin Li, Qiang Guo, Zhiyong Wang, Yuanqi Gong, Yunlin Song, Kejian Qian, Yongjian Feng, Baocai Fu, Xueyan Liu, Zhiping Li, Chuanyong Gong, Cheng Sun, Jian Yu, Zhongzhi Tang, Linxi Huang, Biao Ma, Zhijie He, Qingshan Zhou, Rongguo Yu, Zhihui Tong, Weiqin Li, for the Chinese Critcal Care Nutrition Trials Group (CCCNTG), Lu Ke, Jiajia Lin, Zhihui Tong, Weiqin Li, Yang Wang, Juan Xing, Zhongheng Zhang, Feng Guo, Tao Chen, Lixin Zhou, Dongpo Jiang, Qindong Shi, Jiandong Lin, Jun Liu, Aibin Cheng, Yafeng Liang, Peiyang Gao, Junli Sun, Wenming Liu, Zhenyu Yang, Rumin Zhang, Wei Xing, An Zhang, Zhigang Zhou, Tingfa Zhou, Yang Liu, Fei Tong, Qiuhui Wang, Aijun Pan, Xiaobo Huang, Chuming Fan, Weihua Lu, Dongwu Shi, Lei Wang, Wei Li, Liming Gu, Yingguang Xie, Rongqing Sun, Lin Han, Lihua Zhou, Xiangde Zheng, Feng Shan, Liandi Li, Jianbo Liu, Yuhang Ai, Yan Qu, Hailing Li, Zhiguo Pan, Donglin Xu, Zhiqiang Zou, Yan Gao, Chunli Yang, Qiuye Kou, Xijing Zhang, Jinglan Wu, Chuanyun Qian, Weixing Zhang, Minjie Zhang, Yongjian Feng, Yuan Zong, Bingyu Qin, Fusen Zhang, Zhe Zhai, Yun Sun, Ping Chang, Bo Yu, Min Yu, Shiying Yuan, Yijun Deng, Liyun Zhao, Bin Zang, Yuanfei Li, Fachun Zhou, Xiaomei Chen, Min Shao, Weidong Wu, Ming Wu, Zhaohui Zhang, Yimin Li, Qiang Guo, Zhiyong Wang, Yuanqi Gong, Yunlin Song, Kejian Qian, Baocai Fu, Xueyan Liu, Zhiping Li, Chuanyong Gong, Cheng Sun, Jian Yu, Zhongzhi Tang, Linxi Huang, Biao Ma, Zhijie He, Qingshan Zhou, Rongguo Yu

**Affiliations:** 1 Department of Critical Care Medicine, Jinling Hospital, No. 305 Zhongshan East Road, Nanjing, 210000 Jiangsu Province China; 2 National Institute of Healthcare Data Science, Nanjing University, Nanjing, China; 3 Northern Clinical School, Royal North Shore Hospital, University of Sydney, Sydney, Australia; 4 Department of Intensive Care Medicine, Gelderse Vallei Hospital, Willy Brandtlaan 10, 6716 RP Ede, The Netherlands; 5 Department of Medical Research and Biometrics Center, State Key Laboratory of Cardiovascular Disease, Fuwai Hospital, National Center for Cardiovascular Diseases, Chinese Academy of Medical Sciences and Peking Union Medical College, Beijing, China; 6 Benq Medical Center, Nanjing, China; 7 Department of Emergency Medicine, Sir Run Run Shaw Hospital, Zhejiang University School of Medicine, Hangzhou, China; 8 Tropical Clinical Trials Unit, Department of Clinical Sciences, Liverpool School of Tropical Medicine, Liverpool, L3 5QA UK; 9 First People’s Hospital of Foshan, Foshan, China; 10 Daping Hospital, Army Medical University, Chongqing, China; 11 First Affiliated Hospital of Xi’an Jiao Tong University, Xi’an, China; 12 First Affiliated Hospital of Fujian Medical University, Fuzhou, China; 13 Suzhou Municipal Hospital, Suzhou, China; 14 North China University of Science and Technology Affiliated Hospital, Tangshan, China; 15 Qindao University Medical College Affiliated Yantai Yuhuangding Hospital, Yantai, China; 16 Chengdu University of Traditional Chinese Medicine Affiliated Hospital, Chengdu, China; 17 Luoyang Central Hospital Affiliated To Zhengzhou University, Luoyang, China; 18 Changzhou No. 2 People’s Hospital Affiliated to Nanjing Medical University, Changzhou, China; 19 The Second Affiliated Hospital of Harbin Medical University, Harbin, China; 20 Zibo Central Hospital, Zibo, China; 21 Department of Intensive Care Medicine, The Third Xiangya Hospital of Central South University, Changsha, China; 22 The Second Affiliated Hospital of Chongqing Medical University, Chongqing, China; 23 First People’s Hospital of Kunming, Kunming, China; 24 Linyi City People Hospital, Shandong, China; 25 Tangshan Gongren Hospital, Tangshan, China; 26 Hebei Medical University Second Affiliated Hospital, Shijiazhuang, China; 27 Wuxi People’s Hospital, Wuxi, China; 28 Anhui Provincial Hospital, Hefei, China; 29 Department of Critical Care Medicine, Sichuan Provincial People’s Hospital, University of Electronic Science and Technology of China, Chengdu, 610072 China; 30 First People’s Hospital of Yunnan, Kunming, China; 31 Yijishan Hospital of Wannan Medical College, Wuhu, China; 32 Shanxi Provincial People’s Hospital, Taiyuan, China; 33 Shanxi Medical University First Affiliated Hospital, Taiyuan, China; 34 The People’s Hospital of Fujian Province, Fuzhou, China; 35 People’s Hospital of Yuxi City, Yuxi, China; 36 Jining First People’s Hospital, Jining, China; 37 The First Affiliated Hospital of Zhengzhou University, Zhengzhou, China; 38 Department of Critical Care Medicine, Sir Run Run Shaw Hospital, Zhejiang University School of Medicine, Hangzhou, China; 39 People’s Hospital of Guangxi Zhuang Autonomous Region, Nanning, China; 40 Affiliated Hospital of Inner Mongolia Medical College, Huhehaote, China; 41 Dazhou Central Hospital, Dazhou, China; 42 Qindao University Medical College Affiliated Hospital, Qindao, China; 43 Inner Mongolia People’s Hospital, Huhehaote, China; 44 Xiangya Hospital Central South University, Changsha, China; 45 Qingdao Municipal Hospital Group, Qingdao, China; 46 No.971 Hospital of People’s Liberation Army Navy, Qingdao, China; 47 General Hospital of Southern Theatre Command, Guangzhou, China; 48 Guangzhou First People’s Hospital, School of Medicine, South China University of Technology, Guangzhou, Guangdong China; 49 Union Hospital of Fujian Medical University, Fuzhou, China; 50 The General Hospital of Shenyang Military, Shenyan, China; 51 Jiangxi Provincial People’s Hospital, Nanchang, China; 52 The Sixth Affiliated Hospital, Sun Yat-Sen University, Guangzhou, China; 53 Department of Anaesthesiology and Perioperative Medicine, Xijing Hospital, The Fourth Military Medical University, Xi’an, China; 54 Shenzhen Nanshan People’s Hospital, Shenzhen, China; 55 Kuming Medical University First Affiliated Hospital, Kuming, China; 56 Peking University Shenzhen Hospital, Guandong, China; 57 General ICU, Jinan University First Affiliated Hospital, Jinan, China; 58 Shaanxi Provincial People’s Hospital, Xi’an, China; 59 Henan Provincial People’s Hospital, Zhengzhou, China; 60 Tai’an City Central Hospital, Tai’an, China; 61 The Fourth Hospital of Harbin Medical University, Harbin, China; 62 Anhui Medical University Second Affiliated Hospital, Hefei, China; 63 Southern Medical University Zhujiang Hospital, Guangzhou, China; 64 Department of Critical Care Medicine, the Second Xiangya Hospital of Central South University, Changsha, 410000 Hunan China; 65 First People’s Hospital of Yichang, Yichang, China; 66 Union Hospital Affiliated to Tongji Medical College of Huazhong University of Science and Technology, Wuhan, China; 67 Yancheng First People’s Hospital, Yancheng, China; 68 Guangdong Second Traditional Chinese Medicine Hospital, Guangzhou, China; 69 China Medical University Affiliated Shengjing Hospital, Shenyang, China; 70 Changsha Central Hospital Affiliated to University of South China, Changsha, China; 71 Chongqing Medical University First Affiliated Hospital, Chongqing, China; 72 Department of Critical Care Medicine, Qilu Hospital, Cheeloo College of Medicine, Shandong University, Jinan, China; 73 The First Affiliated Hospital of Anhui Medical University, Hefei, China; 74 Shanxi Bethune Hospital, Taiyuan, China; 75 Health Science Center, The Second People’s Hospital of Shenzhen, First Affiliated Hospital of Shenzhen University, Shenzhen, China; 76 Yichang Central People’s Hospital, Yichang, China; 77 First Affiliated Hospital of Guangzhou Medical University, Guangzhou, China; 78 First Affiliated Hospital of Soochow University, Suzhou, China; 79 Hebei Medical University, Third Affiliated Hospital, Shijiazhuang, China; 80 The Second Affiliated Hospital of Nanchang University, Jiangxi, China; 81 The First Affiliated Hospital of Xinjiang Medical University, Xinjiang, China; 82 The First Affiliated Hospital of Nanchang University, Jiangxi, China; 83 Neurosurgical ICU, Jinan University First Affiliated Hospital, Jinan, China; 84 Yantai Mountain Hospital, Yantai, China; 85 Shenzhen People’s Hospital, Shenzhen, China; 86 Hunan Provincial People’s Hospital, Changsha, China; 87 Tianjing Hospital of Integration of Chinese and Western Medicine, Tianjing, China; 88 Guangdong Provincial People’s Hospital, Guangdong Academy of Medical Sciences, Guangzhou, China; 89 The Second Hospital of Dalian Medical University, Liaoning, China; 90 Wuhan General Hospital of Guangzhou Military Region, Wuhan, China; 91 Shantou University Medical College First Affiliated Hospital, Shantou, China; 92 Jining Medical College Affiliated Hospital, Jining, China; 93 Sun Yat-Sen Memorial Hospital, Sun Yat-Sen University, Guangzhou, China; 94 Hubei Provincial People’s Hospital, Wuhan, China; 95 Fujian Provincial Hospital, Fujian, China; 96 Chinese Critcal Care Nutrition Trials Group (CCCNTG), No. 22 Hankou Road, Nanjing, 210093 China

**Keywords:** Intensive care unit, Cluster-randomized trial, Nutrition therapy, Evidence-based guideline

## Abstract

**Background:**

Previous cluster-randomized controlled trials evaluating the impact of implementing evidence-based guidelines for nutrition therapy in critical illness do not consistently demonstrate patient benefits. A large-scale, sufficiently powered study is therefore warranted to ascertain the effects of guideline implementation on patient-centered outcomes.

**Methods:**

We conducted a multicenter, cluster-randomized, parallel-controlled trial in intensive care units (ICUs) across China. We developed an evidence-based feeding guideline. ICUs randomly allocated to the guideline group formed a local "intervention team", which actively implemented the guideline using standardized educational materials, a graphical feeding protocol, and live online education outreach meetings conducted by members of the study management committee. ICUs assigned to the control group remained unaware of the guideline content. All ICUs enrolled patients who were expected to stay in the ICU longer than seven days. The primary outcome was all-cause mortality within 28 days of enrollment.

**Results:**

Forty-eight ICUs were randomized to the guideline group and 49 to the control group. From March 2018 to July 2019, the guideline ICUs enrolled 1399 patients, and the control ICUs enrolled 1373 patients. Implementation of the guideline resulted in significantly earlier EN initiation (1.20 vs. 1.55 mean days to initiation of EN; difference − 0.40 [95% CI − 0.71 to − 0.09]; *P* = 0.01) and delayed PN initiation (1.29 vs. 0.80 mean days to start of PN; difference 1.06 [95% CI 0.44 to 1.67]; *P* = 0.001). There was no significant difference in 28-day mortality (14.2% vs. 15.2%; difference − 1.6% [95% CI − 4.3% to 1.2%]; *P* = 0.42) between groups.

**Conclusions:**

In this large-scale, multicenter trial, active implementation of an evidence-based feeding guideline reduced the time to commencement of EN and overall PN use but did not translate to a reduction in mortality from critical illness.

*Trial registration:* ISRCTN, ISRCTN12233792. Registered November 20th, 2017.

**Supplementary Information:**

The online version contains supplementary material available at 10.1186/s13054-022-03921-5.

## Introduction

Major international evidence-based guidelines consistently recommend that early targeted nutrition therapy should be provided to critically ill patients [1, 2]. However, multicenter cluster-randomized controlled trials (cRCTs) evaluating the impact of implementing evidence-based guidelines for early targeted nutrition therapy do not consistently show patient benefits [3–5]. Therefore, a considerable gap exists between international guideline recommendations and actual clinical practice [6, 7].

We developed an evidence-based practical feeding guideline to overcome barriers and enhance nutrition therapy in Chinese intensive care units (ICUs) [8]. A pilot before-and-after study (*N* = 410) showed that active implementation of the guideline was effective in increasing enteral nutrition (EN) delivery, thus warranting a large-scale, sufficiently powered study to ascertain effects on patient-centered outcomes [9].

The purpose of this study was to determine the effect of actively implementing an evidence-based feeding guideline on patient outcomes. Using a cluster-randomized design, participating ICUs were randomized to receive the active implementation package or remain as controls. We hypothesized that successful implementation of this guideline could enhance nutrition delivery, and therefore reduce 28-day mortality.

## Methods

### Trial design and oversight

This investigator-initiated, cluster-randomized, parallel-controlled trial assessed the effects of an actively implemented evidence-based guideline for nutrition therapy to control usual care on patient outcomes. The study was approved by the ethics committee of Jinling Hospital (trial sponsor) and registered in the ISRCTN registry before enrollment commenced (Ethical Number: 22017NZKY-019-02; ISRCTN Registry Identifier: ISRCTN12233792). The local hospital ethics committees of all the participating sites also approved the trial. At each site, informed consent was obtained from the patients or their next of kin before enrollment. Patients were enrolled from March 26th, 2018 (the first recruitment) to July 4th, 2019 (the last recruitment). The last patient’s follow-up was completed on July 31st, 2019.

The study was funded by the Key Research and Development Program Foundation of Jiangsu Province of China (no. BE2015685) and Nutricia, Wuxi, China, which supported meetings and training during the study period. The funders had no role in the study's design, data collection, analysis, or preparation of the manuscript. Representatives from Nutricia received copies of the paper before submission for publication but had no influence over content. The trial protocol and statistical analysis plan are available in Additional file 1. The dates of each protocol version, the changes made during each update, and the other details are also provided in Additional file 1.

### Participants

Patients admitted to the participating ICUs were eligible for inclusion if they were 18 years or older, were in the ICU less than 24 h, had one or more organ system failures (sequential organ failure assessment (SOFA) score for any individual organ system ≥ 2), were expected to stay in ICU for more than seven days, and were judged not likely to resume oral diet within three days. Patients who received EN in the past three days, were receiving palliative treatment, were expected to die within 48 h, were pregnant, had a long-term history of steroid use or other immunosuppressive agents, or were receiving radiotherapy or chemotherapy due to malignant diseases were not eligible for inclusion.

### Randomization and masking

Randomization was performed at ICU level. All the participating sites were stratified within province/state based on the type of ICU (emergency, medical, surgical, neurosurgery, and general). Randomization occurred in a 1:1 fashion (guideline group and control group) for the participating ICUs within the same strata using computer-generated random numbers. Allocation concealment was maintained by conducting randomization after hospital consent to participate was obtained.

### Implementation of the feeding guideline

**Fig. 1:**
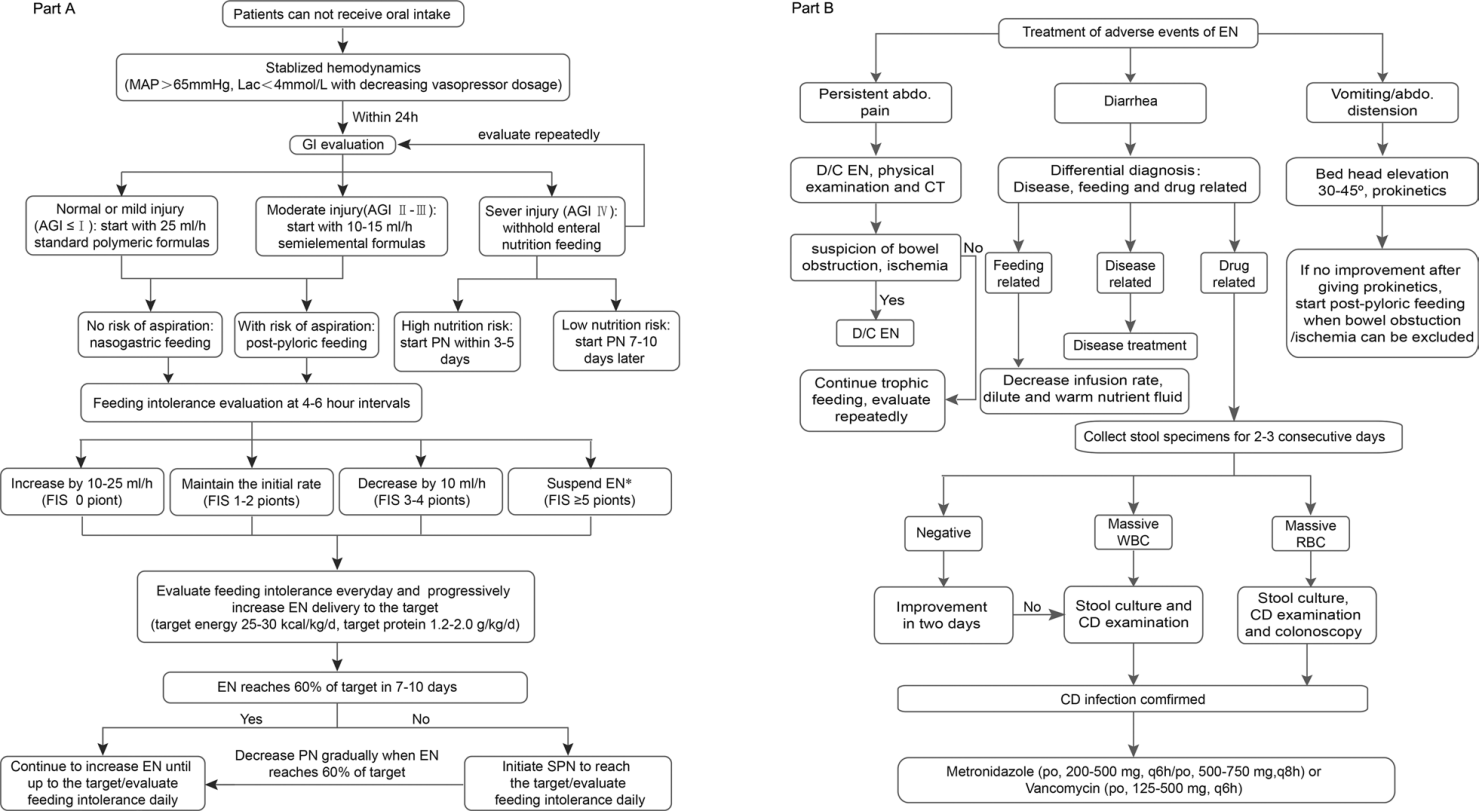
Evidence-based feeding guideline. **A** algorithm of the evidence-based feeding guideline. Feeding intolerance evaluation was implemented using the feeding intolerance score (Additional file 2: Table S1). GI denotes gastrointestinal, AGI denotes acute gastrointestinal injury, PN denotes parenteral nutrition, EN denotes enteral nutrition, and FIS denotes feeding intolerance score. **B** treatment of feeding intolerance. WBC denotes white blood cells, RBC denotes red blood cells, CD denotes *Clostridium difficile*, and D/C denotes discontinue

An up-to-date, evidence-based feeding guideline was developed by reviewing major international guidelines and conducting an updated literature search to include Chinese language publications[1, 10]. The guideline was finalized in April 2016 and tested in a small before-and-after study (*N* = 410) [9]. The graphical feeding protocol representing the guideline recommendations is presented in Fig. 1 (see the adjunct table in Additional file 2: Table S1). Briefly, the protocol includes when to start EN, when to adjust feeding rate, when to start parenteral nutrition and how to manage feeding intolerance. The major aims of this protocol include promoting early EN, standardizing the application of PN (avoiding universal early PN), and increasing target-reaching rate in the first week of ICU admission, as to address the major issues shown in our cross-sectional study [7].

Standardized educational materials were developed to facilitate the implementation of the feeding guideline in ICUs assigned to the study group [9]. A series of educational meetings were organized for all primary site investigators. The primary site investigators were responsible for the distribution, detailing, training, and implementation of the guideline at each center. Each center formed an "intervention team" led by the investigator, including local physicians, nurses, and dietitians. Paper materials, including the graphic feeding protocol and a checklist, were developed and distributed to all intervention sites. The intervention team was responsible for placing these materials at the bedside and in highly visible locations in the ICUs as passive reminders. Live online educational outreach meetings were arranged at request to maintain communication among the management committee and the local investigators. Members of the management committee were required to reply to any queries raised by a site investigator within 24 h.

ICUs assigned to the control group collected data but remained unaware of the contents of the feeding guideline throughout the study period.

### Data collection

A web-based database (Unimed Scientific Inc., Wuxi, China) was developed for data collection. Before enrollment, a start-up meeting for data entry and storage training was organized for all site investigators and research coordinators on March 20^th^, 2018.

### Trial outcomes

The primary study outcome was all-cause mortality within 28 days of enrollment; the secondary outcomes included: process measures of guideline uptake, organ failure related outcomes and corresponding therapies, ICU-free days within 28 days, the incidence of new infections. Detailed definitions of all outcome measures are provided in the study protocol (Additional file 1).

### Statistical analysis

In the previous cluster-randomized trials evaluating the effect of the use of a nutrition guideline on mortality, the 95% CI reported in the ACCEPT nutrition guidelines trial ranges from a 21% to a 0.002% reduction [5]. The ANZ guidelines trial 95% CI ranges from a 6.3% reduction to a 12% increase [3], and another guidelines cRCT conducted in Canada, 95% CI, ranged from a 14% reduction to 13% increase [4]. Simple pooling of the upper estimates of mortality benefit [(21 + 6.3 + 14)/3] reveals it could be reasonable to expect a 13.7% absolute (45% relative) reduction in mortality. Assuming 20% [7] mortality in the control group, a conservative 40% relative (8% absolute) treatment effect was assumed possible with an inter-class correlation of 0.1 [5]. Under these assumptions, a trial with 2,250 participants from 90 ICUs would achieve 80% power to detect the anticipated 8% mortality reduction (CRTSize, Rotondi 2009, version 1.0).

All analyses followed the intention-to-treat principle and were adjusted for clustering. Comparisons between the two groups were made using a mixed-effect model for the primary outcome and key secondary outcomes (ICU-free days within 28 days and the incidence of new infections), adjusting for the clustered nature of the data. Other secondary outcomes and baseline characteristics were compared by Chi-square test or *t*-test as appropriate, with the adjustment for the effects of clustering. Baseline imbalances in potentially confounding variables (*P* < 0.10) were addressed using an appropriately adjusted multivariable model for the primary outcome in additional sensitivity analysis. Two-sided 5% significance levels were used to identify statistically significant results. Analyses were conducted using SAS 9.4®.

## Results

### Results of recruitment

**Fig. 2:**
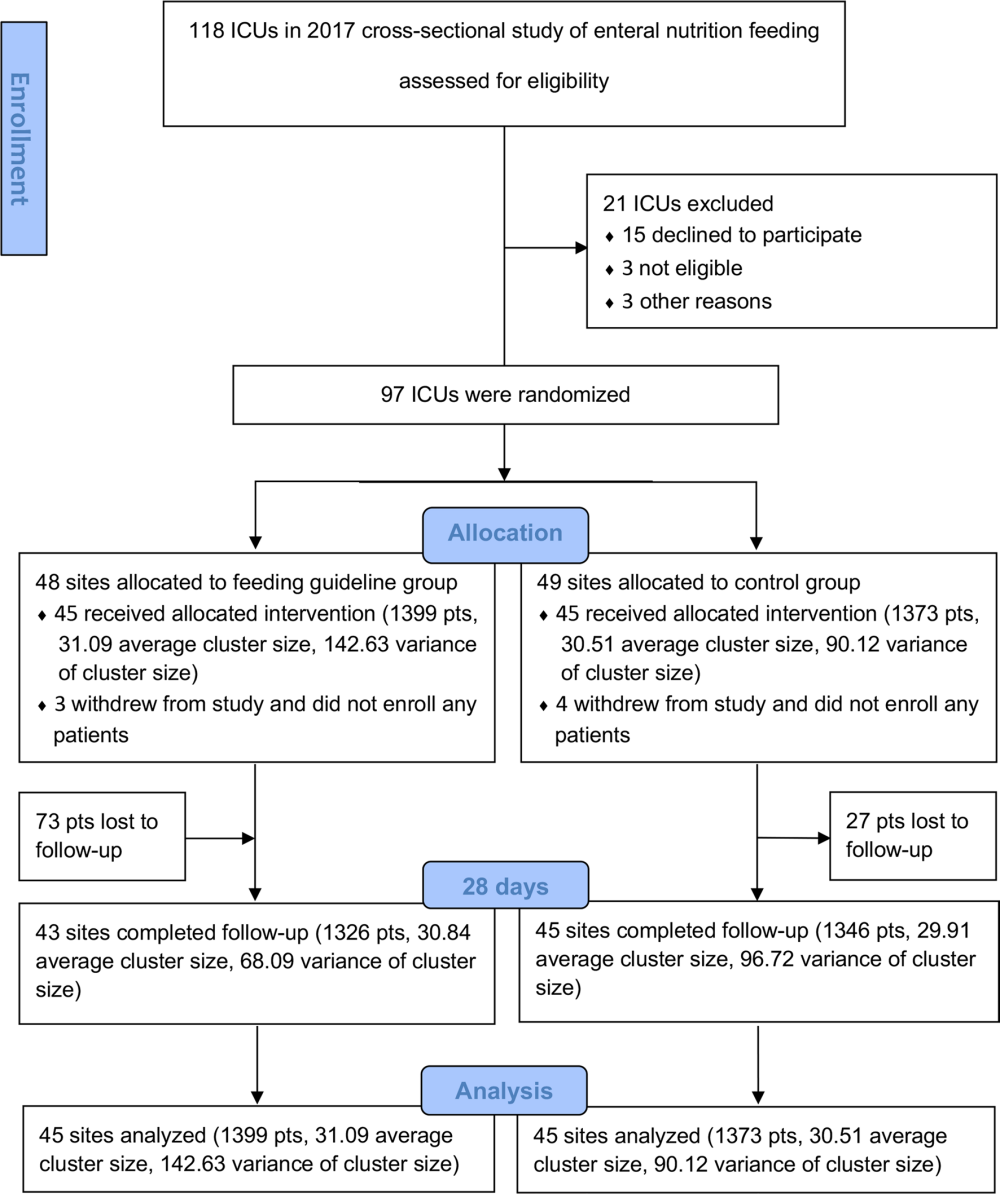
The flow of clusters (ICUs) and participants (patients) through the trial. ICU denotes intensive care unit

In total, 118 ICUs from 22 provinces/states were contacted for participation: 15 ICUs declined to participate in the trial, three failed to obtain ethics approval in time, and three were excluded because they had recently implemented a similar feeding guideline. We randomized 97 ICUs, as shown in the CONSORT flow (Fig. 2). After randomization, seven ICUs (three in the intervention group and four in the control group) withdrew from the study before enrolling any patients. Overall, 2,772 patients were enrolled from 90 ICUs (Additional file 2: Table S2). Twenty-eight day mortality was unavailable in 3.6% of patients (100/2,772, Fig. 2).

### Baseline characteristics

The baseline patient-level clinical characteristics were well balanced, except for SOFA score, abdominal infections, and proportion of patients with Acute Gastrointestinal Injury (AGI) score grade III. (Table 1, See Additional file 3 for additional information of baseline characteristics).

**Table 1:** Baseline ICU and Patient-Level Characteristics

Characteristics	Feeding guideline 48 ICUs, 1399 pts	Control 49 ICU, 1373 pts	*P* value
*ICU-level characteristics*			
Tertiary, No. (%)	34 (70.8)	37 (75.5)	0.61
ICU type, No. (%)			0.97
Emergency	2 (4.2)	2 (4.1)	
Medical	1 (2.1)	1 (2)	
Neuro	1 (2.1)	1 (2)	
Surgical	2 (4.2)	2 (4.1)	
General	42 (87.5)	43 (87.8)	
*Patient-level characteristics*			
Age, mean ± SD, y	61.0 ± 17.6	60.1 ± 17.7	0.98
Male, No. (%)	938 (67.0%)	928 (67.6%)	0.56
BMI, mean ± SD, kg/m^2^	22.8 ± 3.2	23.1 ± 3.2	0.27
APACHE II score, mean ± SD	18.3 ± 6.8	18.6 ± 7.6	0.63
mNUTRIC score, mean ± SD	4.28 ± 1.96	4.30 ± 2.05	0.97
SOFA score, mean ± SD	7.5 ± 3.4	8.1 ± 3.7	0.07
Proportion of organ failure (SOFA score for individual system ≥ 2), No. (%)			
Respiration	968 (72.1%)	1043 (78.4%)	0.10
Renal	284 (21.2%)	316 (23.7%)	0.46
Cardiovascular	384 (28.6%)	450 (33.8%)	0.52
Proportion of patients receiving organ support, No. (%)			
Mechanical ventilation	921 (68.6%)	966 (72.5%)	0.242
Renal replacement therapy	127 (9.5%)	204 (15.3%)	0.018
Vasoactive drugs	401 (29.9%)	523 (39.3%)	0.051
Gastrointestinal function, No. (%)			0.09
AGI-I	1019 (75.9%)	888 (66.5%)	
AGI-II	236 (17.6%)	290 (21.7%)	
AGI-III	50 (3.7%)	126 (9.4%)	
AGI-IV	37 (2.8%)	31 (2.3%)	
Comorbidities, No. (%)			
Hypertension	617 (44.1%)	574 (41.8%)	0.36
Coronary disease	214 (15.3%)	250 (18.2%)	0.31
Diabetes	236 (16.9%)	265 (19.3%)	0.20
Chronic Respiratory diseases	146 (10.4%)	122 (8.9%)	0.31
Stroke	211 (15.1%)	175 (12.7%)	0.42
Gastrointestinal disease	76 (5.4%)	125 (9.1%)	0.13
Malignant tumor	43 (3.1%)	59 (4.3%)	0.44
Others	524 (37.5%)	497 (36.2%)	0.84

ICU denotes intensive care unit; BMI denotes body mass index; APACHE, acute physiology and chronic health evaluation; mNUTRIC denotes modified nutrition risk in the critically ill; SOFA denotes sequential organ failure assessment; AGI denotes acute gastrointestinal injury

### Process measures

In ICUs allocated to guideline implementation, EN was initiated significantly earlier than in control ICUs (1.20 vs. 1.55 mean days to initiation of EN; difference − 0.40 [95% CI − 0.71 to − 0.09]; *P* = 0.01) with significantly more patients receiving EN within 48 h of ICU admission (772/1,399 vs. 451/1,373 patients, *P* < 0.001). Furthermore, PN initiation was significantly delayed in guideline ICUs (1.29 vs. 0.80 mean days to start of PN; difference 1.06 [95% CI, 0.44 to 1.67]; *P* = 0.001) with significantly fewer patients receiving PN during the first 48 h after enrollment (250/1342 vs. 555/1336 patients, *P* = 0.005). See Table 2 for additional process measures.

**Table 2:** Process measures of nutrition therapy

Process Measures	Feeding guideline 48 ICUs, 1399 pts	Controls 49 ICU, 1373 pts	Difference (95% CI)	*P*
Mean time from enrollment to EN initiation, d, mean ± SD	1.20 ± 1.42	1.55 ± 1.64	− 0.40 [− 0.71, − 0.09]	0.01
Mean time to from enrollment to PN initiation, d, mean ± SD	1.29 ± 1.74	0.80 ± 1.40	1.06 [ 0.44, 1.67]	0.001
Mean nutrition support days within first seven days after enrollment /10 patient-days, mean ± SD				
EN and/or PN	8.29 ± 2.26	8.34 ± 2.43	0.10 [− 0.44, 0.65]	0.71
EN (either alone or combined with PN)	7.51 ± 2.82	6.49 ± 3.42	1.09 [0.46, 1.73]	0.001
PN(either alone or combined with EN)	1.66 ± 3.12	3.72 ± 4.18	− 1.68 [− 2.86, − 0.49]	0.006
Mean energy delivered for patients within first seven days after enrollment / fed patient^*^-days, kcal, mean ± SD				
EN	1070.8 ± 500.6	1015.9 ± 423.5	64.45 [− 49.13,178.04]	0.26
PN	776.5 ± 472.9	829.9 ± 611.1	− 43.21 [− 245.8,159.41]	0.67
Patients never fed during first seven days, No. (%)	7(0.6%)	12(0.9%)	0.2% [− 0.6%; 1.0%]	0.67
Patients received EN during first two days after enrollment, No. (%)	883(65.8%)	687(51.4%)	16.5% [ 7.0%; 25.9%]	< 0.001
Patients received PN during first two days after enrollment, No. (%)	250(18.6%)	555(41.5%)	− 19.5% [− 33.1%; − 5.9%]	0.005
Patients fed during first two days after enrollment, No. (%)	1036(77.2%)	1042(78.0%)	0.7% [− 8.4%; 9.9%]	0.87
Patients received EN or PN first two days after enrollment, No. (%)	1022(76.2%)	1006(75.3%)	3.2% [− 6.0%; 12.5%]	0.49
EN tolerance score after enrollments, mean ± SD				
Day 1	0.2 ± 0.8	0.2 ± 0.8	− 0.03 [− 0.23, 0.16]	0.74
Day 2	0.3 ± 0.9	0.3 ± 1.0	− 0.05 [− 0.24, 0.14]	0.62
Day 3	0.4 ± 1.0	0.4 ± 1.0	− 0.02 [− 0.20, 0.16]	0.85
Day 4	0.3 ± 0.9	0.4 ± 1.1	− 0.06 [− 0.23, 0.11]	0.47
Day 5	0.3 ± 0.9	0.4 ± 1.1	− 0.11 [− 0.25, 0.04]	0.16
Day 6	0.3 ± 0.9	0.4 ± 1.0	− 0.08 [− 0.23, 0.07]	0.30
Day 7	0.3 ± 0.9	0.4 ± 1.0	− 0.10 [− 0.25, 0.05]	0.18
Days requiring prokinetic agents within first seven days enrollment /10 patient-days, mean ± SD	1.1 ± 2.7	1.0 ± 2.5	0.37 [− 0.29, 1.03]	0.26
Proportion of patients who received a post-pyloric feeding tube (patients receiving EN) within first seven days after enrollment, No. (%)	91(6.5%)	149(10.9%)	− 3.1% [− 9.3%; 3.1%]	0.32

EN denotes enteral nutrition, and PN denotes parenteral nutrition

*Fed patients denotes patients who received oral intake, EN or PN, either alone or in combination

**Fig. 3:**
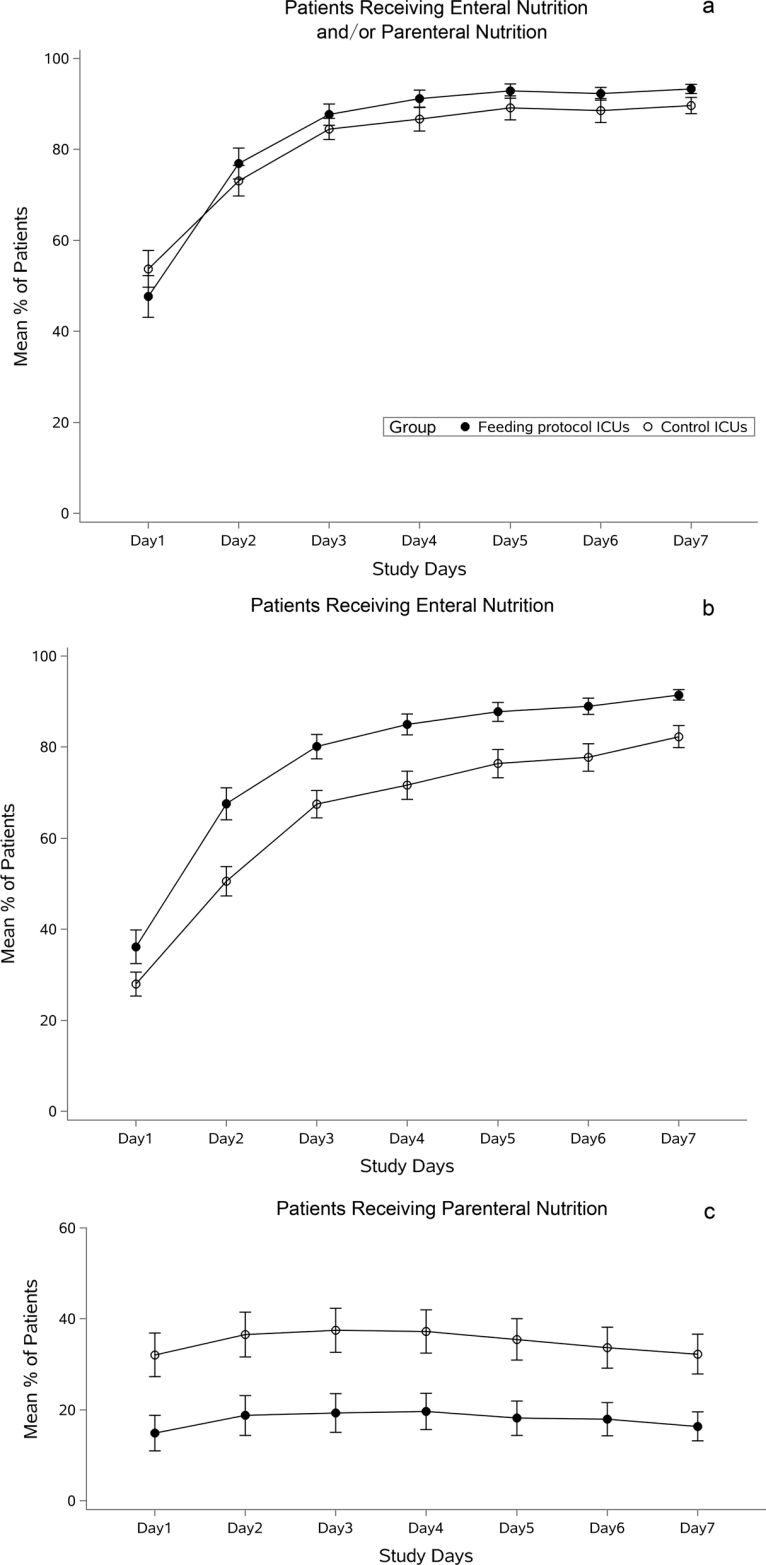
Nutritional support within the first seven days after enrollment. Error bars indicate test-based 95% confidence intervals (adjusted for cluster effect). **a** Proportion of patients receiving enteral and/or parenteral nutrition. *P* > 0.05 (adjusted for cluster effect) between feeding protocol and control groups at each day from day 1 to day 7. **b** Proportion of patients receiving enteral nutrition. *P* < 0.05 (adjusted for cluster effect) between feeding protocol and control groups at each day within seven days of enrollment except day 1. **c** Proportion of patients receiving parenteral nutrition P < 0.05 (adjusted for cluster effect) between feeding protocol and control groups at each day within seven days of enrollment

During the first seven days of enrollment, significantly more patients in the guideline ICUs received EN from day 2 to day 7 (Fig. 3b). Correspondingly, fewer patients received PN on each day of the first seven days of enrollment (Fig. 3c). The proportion of total daily energy delivered by EN was significantly higher in the guideline ICUs on each day of the first seven days after enrollment (Additional file 2: Figure S1). Details (rates, means, *P* values, etc.) for each day are reported in Table S3-S6 (Additional file 2). For the proportion of patients reaching 70% of the estimated energy target and daily protein intake from day 1 to day 7 after enrollment, there is no difference between groups (Additional file 2: Tables S7–S8, Figure S2–S3).

### Primary outcome and other clinical outcomes

On crude analysis there was no significant difference in 28-day mortality (14.2% vs. 15.2%; difference − 1.6% [95%CI − 4.3% to 1.2%]; *P* = 0.42) between study groups. Multivariable analysis controlling for the stratification factors (province/state and type of ICUs) and potentially confounding factors (SOFA score, abdominal infections, and AGI score) did not alter the overall interpretation of the primary outcome (difference, − 0.4% [95% CI  − 5.6% to 4.8%]; *P* = 0.76).

There were no differences in new-onset organ failure within the first seven days after enrollment between groups (Additional file 2: Table S9). ICUs assigned to implement the feeding guideline reported a significantly reduced need for renal replacement therapy (0.97 vs. 1.46 days/10 patient-days; difference − 0.48 days [95%CI − 0.88 to − 0.08 days]; *P* = 0.02) and vasoactive agent use within the first seven days of enrollment (2.19 vs. 2.98 days/10 patient-days; difference − 0.73 days [95%CI − 1.34 to − 0.12 days]; *P* = 0.02).

There was no difference in ICU-free days (9.1 vs. 8.7 days; difference 0.5 [95%CI − 1.0 to 2.0]; *P* = 0.53) or incidence of new infections (6.9% vs. 6.7%; difference 0.1% [95%CI − 1.9% to 2.1%]; *P* = 0.93) between groups. The intracluster correlation coefficient (ICC) and design effects for the primary and key secondary outcomes are shown in Table 3. Serious Adverse Events were reported in one patient from the guideline group and three patients (1/1399 vs. 3/1373, *P* = 0.38) in the control group (Table 3).

**Table 3:** Patient-centered outcomes and adverse events for all enrolled patients

Outcome measure	Feeding guideline 48 ICUs, 1399 pts	Control 49 ICU, 1373 pts	Difference (95% CI)	*P* value	ICC or design effect	
					Feeding guideline	Control
All-cause mortality at day 28, No (%)	188 (14.2%)	205 (15.2%)	− 1.59% [− 4.34%, 1.15%]	0.42	0.11	0.05
ICU-free days within 28 days, d	9.1 ± 8.9	8.7 ± 8.8	0.48 [− 1.02, 1.98]	0.53	0.13	0.14
Incidence of new infections in ICU, No (%)	97 (6.9%)	92 (6.7%)	0.13% [− 1.87%, 2.13%]	0.93	0.21	0.26
Adverse events, no./total no. of events	6	11		0.47		
Gastrointestinal events	4	3		0.66		
Others^*^	2	8		0.32		
Serious adverse events^#^, no./total no. of patients	1	3		0.38		

ICU denotes intensive care unit; CI denotes confidence interval; ICC denotes intraclass correlation coefficient

^*^Others include tachypnea, unplanned urinary catheter removal, aspiration, transient confusion, subcutaneous abscess, decreased muscle strength, mild abdominal bleeding

^#^The serious adverse events were cardiogenic shock in the protocol group and cardiac arrest(one patient), and extremity ischemia(two patients)

## Discussion

We evaluated the effectiveness of an active implementation package supporting an evidence-based feeding guideline for nutrition therapy in critical illness in this 90 ICU cluster-randomized trial. Overall, the successful implementation of the feeding guideline significantly increased early EN delivery and significantly reduced PN use. However, these changes in practice did not influence our primary outcome, 28-day mortality.

Active implementation of an evidence-based feeding guideline has been reported to improve nutrition performance [3–5]. However, the impact on clinical outcomes has been variable in previous cRCTs [3–5], with only one of the three studies showing an improvement in mortality. This study was conducted in 1997–1998 and contained a key recommendation that early EN should commence within 24 h [5]. However, a second larger cRCT undertaken in 2003 that also recommended EN should commence within 24 h of ICU admission failed to duplicate this mortality effect [3]. The third cRCT on this topic recommended early EN should commence within 48 h, like our guideline, however this third cRCT failed to demonstrate a significant effect on any clinical outcomes[4]. Given our current cRCT was powered to detect a meaningful 8% absolute reduction in mortality, the 95% CIs around our estimate of mortality effectively rule out any mortality reduction greater than 4.3% (5.6% after adjustment). None of the previous three cRCTs were adequately powered to detect an effect of this magnitude (Ex. 4.3%).

A previous multicenter cRCT evaluating the active implementation of an evidence-based protocol for nutrition therapy in critical illness demonstrated a significant reduction in the duration of renal failure [3]. This is consistent with our finding of a reduced need for renal replacement therapy, which may be attributed to the renal protective effects of protein administration through maintenance of renal blood flow [11]. However, the overall protein intake in both groups did not achieve the latest threshold recommended by the ESPEN2019(> 1.3 g/kg/d) [12]. Given that secondary endpoints are not adjusted for multiplicity of testing, and the need for organ support are not pre-specified secondary outcomes, these findings should be interpreted with caution. Further trials should be undertaken to investigate the relationship between protein intake and kidney injury.

### Practice change

Implementation of our feeding guideline resulted in comprehensive practice changes across the participating ICUs, marked by significantly earlier EN delivery and reduced PN use. The benefits of early EN have been well addressed in multiple critically ill populations [2], and achieving earlier EN commencement is one of the primary aims of this feeding guideline. A discrepancy between our feeding guideline and those used in the previous cRCTs [3–5] is that we clearly discouraged early initiation of PN if EN was not feasible in patients with low nutrition risk, according to the recommendations made by the ASPEN/SCCM guidelines [1]. Early initiation of PN is costly and may result in worse outcomes, as shown in the EPaNIC trial [13]. However, the overall improvement in early EN and reduction in PN use achieved in our study was modest and did not translate into improvements in mortality or a reduction of new infections. This failure to impact the onset of new infections is consistent with the results of the previous large trials, which also found no impact of PN on infectious complications [14, 15].

The most recent ESPEN guidelines recommend that clinicians should strive to provide more than 70% of a patient's calculated energy targets by ICU day 4 [12]. In our study, active implementation of the feeding guideline did not result in significantly more patients achieving this goal, although the proportion of EN-delivered energy did increase. Feeding intolerance is a significant concern impeding the early achievement of energy targets worldwide [16]. Our feeding guideline incorporated a self-developed feeding intolerance score for repeated gastrointestinal function assessment [17]. The feeding intolerance score includes key gastrointestinal symptoms and intra-abdominal pressure, both associated with ICU mortality [18, 19], and we categorized them into four grades of severity for quantitive measurement. Our results suggest that applying this score as a tool for repeated feeding tolerance assessment may have effectively facilitated EN delivery without additional feeding intolerance. We recommend additional individual patient RCTs to evaluate the effectiveness of this intolerance score.

### Limitations

Our study was adequately powered to detect a meaningful difference in the primary outcome, mortality. However, previous studies, including our own before-and-after study (*N* = 410) [9], which investigated nutrition in ICUs, showed that the likeliness of nutrition practice to reduce mortality is very limited [20], which means an estimation of an 8% reduction in mortality might be overoptimistic. Besides, the mortality in the control group is lower than expected (15.2% vs. 20% for sample size estimation), which might make our trial underpowered. Regarding the guideline, we recommend using semi-elemental products to initiate EN in patients with AGI II-III, which is not a common practice and only recommended by a few international nutrition guidelines [4]. This practice may impact the generalizability of our results to come countries. Moreover, although 32% of patients at standard care hospitals received PN on the first day of enrollment, this was reduced to 15% of patients under guideline care. This level of PN use in our guideline hospitals appears to be similar to standard care in other countries around the world [6, 21]. Furthermore, large-scale clinical trials randomizing critically ill patients to commence EN first vs. PN first establish there is no impact on clinical outcomes or infectious complications [14, 15]. Therefore we suggest that PN use in our participating ICUs does not affect the generalizability of our results.

From a technical perspective, although the active implementation package supporting the feeding guideline resulted in measurable and meaningful differences in practice, the guidelines are complex and make multiple clinical recommendations. Because of this complexity, we cannot attribute specific changes in clinical outcomes to any specific guideline recommendation, we can only hypothesize such a relationship may exist. Any such hypothesis must be tested in an individual patient RCT evaluating specific clinical outcomes and specific clinical recommendations.

## Conclusions

In conclusion, successful active implementation of an evidence-based feeding guideline reduced the time to commencement of EN and overall PN use but did not translate into a reduction in our primary outcome, 28-day all-cause mortality. Additional research is warranted to investigate the impact of enhanced nutrition, especially protein, on other outcomes.

## Supplementary Information

**Additional file 1**. Protocol and Statistical Analysis Plan.

**Additional file 2**. **Table S1**. Feeding intolerance score. **Table S2**. Recruitment of patients. **Table S3**. Proportion of patients receiving enteral and/or parenteral nutrition. **Table S4**. Proportion of patients receiving enteral nutrition. **Table S5**. Proportion of patients receiving parenteral nutrition. **Table S6**. Proportion of enteral nutrition delivered energy in daily energy delivery. **Table S7**. Target-reaching rate in fed patients from day1 to day7 after enrollment. **Table S8**. Protein intake from day1 to day7 after enrollment. **Table S9**. Organ failure-related outcomes. **Figure S1**. Proportion of enteral nutrition delivered energy in daily energy delivery within the first seven days after enrollment. **Figure S2**. Target-reaching (more than 70% of the estimated energy target) rate in fed patients for energy delivery from day1 to day7 after enrollment. **Figure S3**. Daily protein intake from day1 to day7 after enrollment.

**Additional file 3**. Continued Table 1. Baseline ICU and Patient-Level Characteristics..

## Data Availability

The datasets generated and analyzed in this article are not publicly available due to health privacy concerns but are available from the corresponding author on reasonable request.

## Abbreviations

cRCT: Cluster-randomized controlled trials
ICU: Intensive care unit
EN: Enteral nutrition
PN: Parenteral nutrition
SOFA: Sequential organ failure assessment
AGI: Acute gastrointestinal injury
ICC: Intracluster correlation coefficient
FIS: Feeding intolerance score
