# Correction to: Actively implementing an evidence-based feeding guideline for critically ill patients (NEED): a multicenter, cluster-randomized, controlled trial

**DOI:** 10.1186/s13054-022-03982-6

**Published:** 2022-04-21

**Authors:** Lu Ke, Jiajia Lin, Gordon S. Doig, Arthur R. H. van Zanten, Yang Wang, Juan Xing, Zhongheng Zhang, Tao Chen, Lixin Zhou, Dongpo Jiang, Qindong Shi, Jiandong Lin, Jun Liu, Aibin Cheng, Yafeng Liang, Peiyang Gao, Junli Sun, Wenming Liu, Zhenyu Yang, Rumin Zhang, Wei Xing, An Zhang, Zhigang Zhou, Tingfa Zhou, Yang Liu, Fei Tong, Qiuhui Wang, Aijun Pan, Xiaobo Huang, Chuming Fan, Weihua Lu, Dongwu Shi, Lei Wang, Wei Li, Liming Gu, Yingguang Xie, Rongqing Sun, Feng Guo, Lin Han, Lihua Zhou, Xiangde Zheng, Feng Shan, Jianbo Liu, Yuhang Ai, Yan Qu, Liandi Li, Hailing Li, Zhiguo Pan, Donglin Xu, Zhiqiang Zou, Yan Gao, Chunli Yang, Qiuye Kou, Xijing Zhang, Jinglan Wu, Chuanyun Qian, Weixing Zhang, Minjie Zhang, Yuan Zong, Bingyu Qin, Fusen Zhang, Zhe Zhai, Yun Sun, Ping Chang, Bo Yu, Min Yu, Shiying Yuan, Yijun Deng, Liyun Zhao, Bin Zang, Yuanfei Li, Fachun Zhou, Xiaomei Chen, Min Shao, Weidong Wu, Ming Wu, Zhaohui Zhang, Yimin Li, Qiang Guo, Zhiyong Wang, Yuanqi Gong, Yunlin Song, Kejian Qian, Yongjian Feng, Baocai Fu, Xueyan Liu, Zhiping Li, Chuanyong Gong, Cheng Sun, Jian Yu, Zhongzhi Tang, Linxi Huang, Biao Ma, Zhijie He, Qingshan Zhou, Rongguo Yu, Zhihui Tong, Weiqin Li, for the Chinese Critical Care Nutrition Trials Group (CCCNTG), Lu Ke, Jiajia Lin, Zhihui Tong, Weiqin Li, Yang Wang, Juan Xing, Zhongheng Zhang, Feng Guo, Tao Chen, Lixin Zhou, Dongpo Jiang, Qindong Shi, Jiandong Lin, Jun Liu, Aibin Cheng, Yafeng Liang, Peiyang Gao, Junli Sun, Wenming Liu, Zhenyu Yang, Rumin Zhang, Wei Xing, An Zhang, Zhigang Zhou, Tingfa Zhou, Yang Liu, Fei Tong, Qiuhui Wang, Aijun Pan, Xiaobo Huang, Chuming Fan, Weihua Lu, Dongwu Shi, Lei Wang, Wei Li, Liming Gu, Yingguang Xie, Rongqing Sun, Lin Han, Lihua Zhou, Xiangde Zheng, Feng Shan, Liandi Li, Jianbo Liu, Yuhang Ai, Yan Qu, Hailing Li, Zhiguo Pan, Donglin Xu, Zhiqiang Zou, Yan Gao, Chunli Yang, Qiuye Kou, Xijing Zhang, Jinglan Wu, Chuanyun Qian, Weixing Zhang, Minjie Zhang, Yongjian Feng, Yuan Zong, Bingyu Qin, Fusen Zhang, Zhe Zhai, Yun Sun, Ping Chang, Bo Yu, Min Yu, Shiying Yuan, Yijun Deng, Liyun Zhao, Bin Zang, Yuanfei Li, Fachun Zhou, Xiaomei Chen, Min Shao, Weidong Wu, Ming Wu, Zhaohui Zhang, Yimin Li, Qiang Guo, Zhiyong Wang, Yuanqi Gong, Yunlin Song, Kejian Qian, Baocai Fu, Xueyan Liu, Zhiping Li, Chuanyong Gong, Cheng Sun, Jian Yu, Zhongzhi Tang, Linxi Huang, Biao Ma, Zhijie He, Qingshan Zhou, Rongguo Yu

**Affiliations:** 1 Department of Critical Care Medicine, Jinling Hospital, Medical School of Nanjing University, No. 305, Zhongshan East Road, Nanjing, 210000 Jiangsu Province China; 2 National Institute of Healthcare Data Science, Nanjing University, Nanjing, China; 3 Northern Clinical School, Royal North Shore Hospital, University of Sydney, Sydney, Australia; 4 Department of Intensive Care Medicine, Gelderse Vallei Hospital, Willy Brandtlaan 10, 6716 RP Ede, The Netherlands; 5 Department of Medical Research and Biometrics Center, State Key Laboratory of Cardiovascular Disease, Fuwai Hospital, National Center for Cardiovascular Diseases, Chinese Academy of Medical Sciences and Peking Union Medical College, Beijing, China; 6 Benq Medical Center, Nanjing, China; 7 Department of Emergency Medicine, Sir Run Run Shaw Hospital, Zhejiang University School of Medicine, Hangzhou, China; 8 Tropical Clinical Trials Unit, Department of Clinical Sciences, Liverpool School of Tropical Medicine, Liverpool, L3 5QA UK; 9 First People’s Hospital of Foshan, Foshan, China; 10 Daping Hospital, Army Medical University, Chongqing, China; 11 First Affiliated Hospital of Xi’an Jiao Tong University, Xi’an, China; 12 First Affiliated Hospital of Fujian Medical University, Fuzhou, China; 13 Suzhou Municipal Hospital, Suzhou, China; 14 North China University of Science and Technology Affiliated Hospital, Tangshan, China; 15 Qindao University Medical College Affiliated Yantai Yuhuangding Hospital, Yantai, China; 16 Chengdu University of Traditional Chinese Medicine Affiliated Hospital, Chengdu, China; 17 Luoyang Central Hospital Affiliated to Zhengzhou University, Luoyang, China; 18 Changzhou No. 2 People’s Hospital Affiliated to Nanjing Medical University, Changzhou, China; 19 The Second Affiliated Hospital of Harbin Medical University, Harbin, China; 20 Zibo Central Hospital, Zibo, China; 21 Department of Intensive Care Medicine, The Third Xiangya Hospital of Central South University, Changsha, China; 22 The Second Affiliated Hospital of Chongqing Medical University, Chongqing, China; 23 First People’s Hospital of Kunming, Kunming, China; 24 Linyi City People Hospital, Shandong, China; 25 Tangshan Gongren Hospital, Tangshan, China; 26 Hebei Medical University Second Affiliated Hospital, Shijiazhuang, China; 27 Wuxi People’s Hospital, Wuxi, China; 28 Anhui Provincial Hospital, Hefei, China; 29 Department of Critical Care Medicine, Sichuan Provincial People’s Hospital, University of Electronic Science and Technology of China, Chengdu, 610072 China; 30 First People’s Hospital of Yunnan, Kunming, China; 31 Yijishan Hospital of Wannan Medical College, Wuhu, China; 32 Shanxi Provincial People’s Hospital, Taiyuan, China; 33 Shanxi Medical University First Affiliated Hospital, Taiyuan, China; 34 The People’s Hospital of Fujian Province, Fuzhou, China; 35 People’s Hospital of Yuxi City, Yuxi, China; 36 Jining First People’s Hospital, Jining, China; 37 The First Affiliated Hospital of Zhengzhou University, Zhengzhou, China; 38 Department of Critical Care Medicine, Sir Run Run Shaw Hospital, Zhejiang University School of Medicine, Hangzhou, China; 39 People’s Hospital of Guangxi Zhuang Autonomous Region, Nanning, China; 40 Affiliated Hospital of Inner Mongolia Medical College, Huhehaote, China; 41 Dazhou Central Hospital, Dazhou, China; 42 Qindao University Medical College Affiliated Hospital, Qindao, China; 43 Inner Mongolia People’s Hospital, Huhehaote, China; 44 Xiangya Hospital Central South University, Changsha, China; 45 Qingdao Municipal Hospital Group, Qingdao, China; 46 No.971 Hospital of People’s Liberation Army Navy, Qingdao, China; 47 General Hospital of Southern Theatre Command, Guangzhou, China; 48 Guangzhou First People’s Hospital, School of Medicine, South China University of Technology, Guangzhou, Guangdong China; 49 Union Hospital of Fujian Medical University, Fuzhou, China; 50 The General Hospital of Shenyang Military, Shenyan, China; 51 Jiangxi Provincial People’s Hospital, Nanchang, China; 52 The Sixth Affiliated Hospital, Sun Yat-Sen University, Guangzhou, China; 53 Department of Anaesthesiology and Perioperative Medicine, Xijing Hospital, The Fourth Military Medical University, Xi’an, China; 54 Shenzhen Nanshan People’s Hospital, Shenzhen, China; 55 Kuming Medical University First Affiliated Hospital, Kuming, China; 56 Peking University Shenzhen Hospital, Guandong, China; 57 General ICU, Jinan University First Affiliated Hospital, Jinan, China; 58 Shaanxi Provincial People’s Hospital, Xi’an, China; 59 Henan Provincial People’s Hospital, Zhengzhou, China; 60 Tai’an City Central Hospital, Tai’an, China; 61 The Fourth Hospital of Harbin Medical University, Harbin, China; 62 Anhui Medical University Second Affiliated Hospital, Hefei, China; 63 Southern Medical University Zhujiang Hospital, Guangzhou, China; 64 Department of Critical Care Medicine, The Second Xiangya Hospital of Central South University, Changsha, 410000 Hunan China; 65 First People’s Hospital of Yichang, Yichang, China; 66 Union Hospital Affiliated to Tongji Medical College of Huazhong University of Science and Technology, Wuhan, China; 67 Yancheng First People’s Hospital, Yancheng, China; 68 Guangdong Second Traditional Chinese Medicine Hospital, Guangzhou, China; 69 China Medical University Affiliated Shengjing Hospital, Shenyang, China; 70 Changsha Central Hospital Affiliated to University of South China, Changsha, China; 71 Chongqing Medical University First Affiliated Hospital, Chongqing, China; 72 Department of Critical Care Medicine, Qilu Hospital, Cheeloo College of Medicine, Shandong University, Jinan, China; 73 The First Affiliated Hospital of Anhui Medical University, Hefei, China; 74 Shanxi Bethune Hospital, Taiyuan, China; 75 Health Science Center, The Second People’s Hospital of Shenzhen, First Affiliated Hospital of Shenzhen University, Shenzhen, China; 76 Yichang Central People’s Hospital, Yichang, China; 77 First Affiliated Hospital of Guangzhou Medical University, Guangzhou, China; 78 First Affiliated Hospital of Soochow University, Suzhou, China; 79 Hebei Medical University, Third Affiliated Hospital, Shijiazhuang, China; 80 The Second Affiliated Hospital of Nanchang University, Jiangxi, China; 81 The First Affiliated Hospital of Xinjiang Medical University, Xinjiang, China; 82 The First Affiliated Hospital of Nanchang University, Jiangxi, China; 83 Neurosurgical ICU, Jinan University First Affiliated Hospital, Jinan, China; 84 Yantai Mountain Hospital, Yantai, China; 85 Shenzhen People’s Hospital, Shenzhen, China; 86 Hunan Provincial People’s Hospital, Changsha, China; 87 Tianjing Hospital of Integration of Chinese and Western Medicine, Tianjing, China; 88 Guangdong Provincial People’s Hospital, Guangdong Academy of Medical Sciences, Guangzhou, China; 89 The Second Hospital of Dalian Medical University, Liaoning, China; 90 Wuhan General Hospital of Guangzhou Military Region, Wuhan, China; 91 Shantou University Medical College First Affiliated Hospital, Shantou, China; 92 Jining Medical College Affiliated Hospital, Jining, China; 93 Sun Yat-Sen Memorial Hospital, Sun Yat-Sen University, Guangzhou, China; 94 Hubei Provincial People’s Hospital, Wuhan, China; 95 Fujian Provincial Hospital, Fujian, China; 96 Chinese Critical Care Nutrition Trials Group (CCCNTG), No. 22 Hankou Road, Nanjing, 210093 China

## Correction to: Critical Care (2022) 26:46. https://doi.org/10.1186/s13054-022-03921-5

Following publication of the original article [1], the authors reported an error in one of the affiliations. The revised affiliation is indicated hereafter and the changes have been highlighted in **bold typeface**.

The incorrect affiliation reads:

^1^Department of Critical Care Medicine, Jinling Hospital, No. 305, Zhongshan East Road, Nanjing 210000, Jiangsu Province, China

The correct affiliation should read:

^1^Department of Critical Care Medicine, Jinling Hospital, **Medical School of Nanjing University**, No. 305, Zhongshan East Road, Nanjing 210000, Jiangsu Province, China

All the changes requested are implemented in this correction, and the original article [1] has been corrected.
