# Supplementary material for: Actively implementing an evidence-based feeding guideline for critically ill patients (NEED): a multicenter, cluster-randomized, controlled trial
Source: Crit Care. 2022 Feb 16;26:46. doi: 10.1186/s13054-022-03921-5 (PMC8848648; doi:10.1186/s13054-022-03921-5)
Supplement: Supplementary file 1 — Additional file 1. Protocol and Statistical Analysis Plan. [file 13054_2022_3921_MOESM1_ESM.pdf]

Effectiveness of a feeding protocol on nutritional therapy and clinical outcomes in critically ill patients

Protocol and Statistical Analysis Plan Amendment History

CONTENTS

1. Original protocol, final protocol, summary of changes

1.1. Protocol V1 July 2017 (original protocol) …………………………..... Page 1

1.2. Protocol V2 Feb 2018 (updated before commencement of the study, and changes of eligbility criteria was included in this version) ……….……… Page 28

1.3. Protocol V4 (final protocol) ……………………………………….…... Page 57

1.4. Summary of changes from V1-4……………………………………….. Page 90

2. Original statistical analysis plan (the only version)

2.1. Original statistical analysis plan ……………………………………… Page 97

N.E.D.D

EffectiveNess of a feEding protocol on nutritional thErapy and clinical outcomes in critically ill patients: a multi-center, cluster-ranDomized, parallel-controlled trial

Protocol Ver. 1.0 dated 02. July. 2017

Primary Investigator

Professor. Weiqin Li

Director of surgical intensive care unit, Jinling Hospital, Nanjing University

305 East Zhongshan Rd,210002

Nanjing, Jiangsu province, China

Email: ctgchina@medbit.cn

Phone: +8680860007

Fax: +8680863073

Coordinating Centre

Chinese Critical Care Nutrition Trials Group

Nanjing, Jiangsu province, China

Phone: +8680325431

Table of contents

1.Abrreviations 4
2.Study Administrative information 5
2.1 Steering and management committee 5
2.2 Coordinating and data management center 8
2.3 Writing and publication committee 8
2.4 Data and safety monitoring board 9
2.5 Registration 9
2.6 Funding 9
3. Background and rationale 10
3.1 The role of nutritional therapy in critical care 10
3.2 The rationale for conducting this study 10
4. Study Design 11
4.1 Aim of the study 11
4.2 General study setting 11
4.3 Sample size 11
5. Study population 12
5.1 Patient recruitment 12
5.2 Eligibility criteria 12
6.Randomization and assignment of interventions 13
6.1 Sequence generation 13
6.2 Blinding method 13
6.3. Study procedures 13
7. Outcome measures 14
7.1 Primary outcome measures 14
7.2 Secondary outcome measures 14
8. Data management, analysis, and statistics 15
8.1 Data collection 15
8.2 Participant timeline 15
8.3 Basic principle of analysis 16
8.4 Interim analysis 16
9.Ethics and dissemination 17
9.1 Ethical issues of this study 17
9.2 Potential risks and benefits 17
9.3 Consent and confidentiality 17
9.4 Dissemination policy 17
10. Safety issues and monitoring 18
10.1 Data and safety monitoring board 18
10.2 Adverse events 18
10.3 Serious adverse events 18
10.4 Monitoring of potential adverse events 19
11.References 20
Figure 1: Feeding guideline 22

Figure 2: Protocols for the management of adverse events 23
Table 1: EN tolerance score 24
Appendix 1. Participant of ICUs 25

1.Abrreviations

CDMC	Coordinating and Data Management Center
DSMB	Data and Safety Monitoring Board
SAE	Serious Adverse Events
ICU	Intensive Care Unit
PN	Parenteral Nutrition
EN	Enteral Nutrition
SOFA	Sequential Organ Failure Assessment
MAP	Mean Artery Pressure
CPAP	Continuous Positive Airway Pressure
NIPPV	Non-Invasive Positive Pressure Ventilation
NOK	Next Of Kin
ITT	Intention-to-treat
FAS	Full-Analysis Set
AEs	Adverse Events
AGI	Acute Gastrointestinal injury
CRP	C-reaction protein
RRT	Renal Replacement Therapy
MV	Mechanical Ventilation

2.Study Administrative information

2.1 Steering and management committee

The steering and management committee is responsible for the approval of the full protocol, database, and its related methods. The members of the committee will also oversee the implementation of the study and play an advisory role.

Members of the steering committee are listed below

Name	Email
Prof. Weiqin Li (Primary investigator)	ctgchina@medbit.cn
Prof. Zhihui Tong	njzyantol@hotmail.com
Prof. Lu Ke	ctgkelu@nju.edu.cn
Dr. Zhongheng Zhang	zh_zhang1984@zju.edu.cn
Juan Xing	lasaison@126.com
Lixin Zhou	drzhoulixin@gmail.com
Dongpo Jiang	66dbjiang@163.com
Qindong Shi	shiqindong@163.com
Jiandong Lin	linjd01680067@sina.com
Jun Liu	liujunphd@sina.cn
Aibin Cheng	myfyicu@126.com
Yafeng Liang	13954551363@163.com
Peiyang Gao	GaoPY930@126.com
Junli Sun	zxyysicu@163.com
Wenming Liu	lwmicu@163.com
Zhenyu Yang	Yang_19840214@163.com
Rumin Zhang	sdzhangrm@126.com
Weixing	xy3yyxw@csu.edu.cn
An Zhang	zhangan@hospital.cqmu.edu.cn
Zhigang Zhou	ynkmzzg@163.com
Tingfa Zhou	zhoutingfa66@126.com
Yang Liu	13933358101@139.com
Fei Tong	tongfei168@163.com

Qiuhui Wang	qiuhui4403@sina.com
Aijun Pan	aijunpan@fsyy.ustc.edu.cn
Xiaobo Huang	814129752@qq.com
Chuming Fan	fanchuming@sina.com
Weihua Lu	lwh683@126.com
Dongwu Shi	shidw1@163.com
Lei Wang	413060682@163.com
Wei Li	sicu.lw@163.com
Liming Gu	glm39887@126.com
Yingguang Xie	zzyxkx@163.com
Rongqing Sun	rongqing.sun@126.com
Feng Guo	zjguofeng@aliyun.com
Lin Han	xhan0507@sina.com
Lihua Zhou	xiulika@163.com
Xiangde Zheng	zxdfg123@163.com
Feng Shan	mountain1963@163.com
Jianbo Liu	qicaixia2007@126.com
Yuhang Ai	ayhicu1978@sina.com
Yan Qu	qdquyan@aliyun.com
Liandi Li	liliandi75@sina.com
Hailing Li	lihailing608@163.com
Zhiguo Pan	gzpzg@126.com
Donglin Xu	syicuxdl@126.com
Zhiqiang Zou	396746023@qq.com
Yan Gao	Gaoyan1515@163.com
Chunli Yang	646233247@qq.com
Qiuye Kou	kouqiuye@126.com
Xijing Zhang	zhangxj918@163.com
Jinglan Wu	doctorwu99@126.com
Chuanyun Qian	qianchuanyun@126.com
Weixing Zhang	zhangwx@hotmail.com
Minjie Zhang	hychen427@163.com
Yuan Zong	chuchu20040317@qq.com

Bingyu Qin	nicolasby@126.com
Fusen Zhang	13793812109@126.com
Zhe Zhai	zhaizhe0357@163.com
Yun Sun	sunyun15@163.com
Ping Chang	changp963@163.com
Bo Yu	yuboicu@csu.edu.cn
Min Yu	ycyyicuyumin@163.com
Shiying Yuan	yuan_shiying@163.com
Yijun Deng	dengyj2003@163.com
Liyun Zhao	403756433@qq.com
Bin Zang	zangb@sj-hospital.org
Yuanfei Li	156636305@qq.com
Fachun Zhou	zfc88@126.com
Xiaomei Chen	chenxm008@163.com
Min Shao	shaominmail @yahoo.com
Weidong Wu	13503545010@163.com
Ming Wu	Boshiyy@126.com
Zhaohui Zhang	1410956890@qq.com
Yimin Li	dryiminli@vip.163.com
Qiang Guo	ggqq7910@163.com
Zhiyong Wang	18533112993@126.com
Yuanqi Gong	760225787@qq.com
Yunlin Song	17929294@qq.com
Kejian Qian	2682244275@qq.com
Yongjian Feng	horse1980@163.com
Baocai Fu	kailiaokailiao@sina.com
Xueyan Liu	1736574753@qq.com
Zhiping Li	354855270@qq.com
Chuanyong Gong	chuanyonggong@163.com
Cheng Sun	suncheng2008@139.com
Jian Yu	yujian0427@163.com
Zhongzhi Tang	Jzktzz_wh2014@163.com
Linxi Huang	13502733122@126.com
Biao Ma	305058382@qq.com

Zhijie He	805545775@qq.com
Qingshan Zhou	white1962@qq.com
Rongguo Yu	garyyrg@yahoo.com

2.2 Coordinating and data management center

Coordinating and data management centers (CDMC) was organized before the implementation of the current study. They are responsible for day to day management of the trial, assistance for ethic application in each center, protocol and case report form design, online database design and maintenance, protocol and case report form design, online database design and maintenance, protocol training for the participating centers, enrollment, data entry and quality control, severe adverse event monitor and notification and data analysis. The CDMC plans to meet before enrollment, three months after initial enrollment, and six months after initial enrollment to ensure qualified data entry.

Members of CDMC are listed below

Prof. Zhihui Tong

Prof. Lu Ke

Dr. Jing Zhou

Mr. Yafei Yan

Dr. Juan Xing

Dr. Jiajia Lin

Ms. Yan Chen

2.3 Writing and publication committee

The writing and publication committee is responsible for drafting the manuscript and submission of the manuscript to adequate journals. The Writing and publication committee will also decide on the authorship of this study. After the conclusion of this study, all participating centers are welcome to submit proposals for post-hoc analysis

to the writing and publication committee is responsible for reviewing and rating all the proposals for further analysis.

Members are listed below

Prof. Weiqin Li

Dr. Lu Ke

Dr. Zhongheng Zhang

Ms. Jiajia Lin

2.4 Data and safety monitoring board

Data and safety monitoring board (DSMB) is an independent group of experts that offers advice during the implementation of the study. The DSMB can recommend that a trial be stopped early because of concerns about participant safety.

Members of DSMB are listed below

Prof. Yuxiu Liu

Dr. Mengjie Lu

Prof. Wenkui Yu

Prof. Qiang Li

2.5 Registration

The NEED trial was registered on the ISRCTN registry (ISRCTN12233792) before commencement of the study.

2.6 Funding

The study was funded partly by the Key Research and Development Program Foundation of Jiangsu Province of China (No. BE2015685) and partly by the Nutricia, Wuxi, China.

3. Background and rationale

3.1 The role of nutritional therapy in critical care

In intensive care unit (ICU), nutritional therapy is one of the most crucial treatments for critically ill patients, which may significantly influence the clinical outcomes [1,2]. There is a large body of evidence showing that malnutrition is associated with a significantly increased risk of death. Besides, ICU patients are prone to suffer from underfeeding, which could further exacerbate the existing gap between energy demand and intake [3]. The route of nutrition delivery is another important issue when starting nutrition therapy for ICU patients. Enteral feeding has been repeatedly proven to be superior to parenteral nutrition (PN) with respect to the outcomes such as nosocomial infection, mortality, and also medical resource utilization [4-7].

As nutritional therapy involves a battery of interventions and procedures, a variety of clinical practice guidelines have been published to standardize nutritional therapy. However, it was reported that the adherence to these guidelines was suboptimal so that the clinical practices of feeding vary substantially across different regions and hospitals. Moreover, it remains controversial whether feeding protocol is effective in improving clinical outcomes such as mortality, nosocomial infections, duration of mechanical ventilation, and length of stay in ICU [8-11].

3.2 The rationale for conducting this study

China has the world's largest population of critically ill patients, and the clinical practice of nutrition therapy varies massively. Previous small studies have shown that the proportion of EN was as low as 40% on the second day after ICU admission, which could be potentially improved with the implementation of a feeding protocol [12]. Our previous cross-sectional study showed that the proportions of subjects starting EN within 24, 48 and 72 h after ICU entry was 24.8% (84/352), 32.7%

(150/459) and 40.0% (200/541), suggesting the unsatisfying adherence to the current international guidelines and huge space for improvement [13]. A cluster-randomized method can effectively control the confounding factors between groups to obtain more reliable conclusions. In addition, the interventional study of feeding protocol would lead to the pollution effect. Randomization at the hospital level and appropriate stratification could avoid the pollution.

4. Study Design

4.1 Aim of the study

We aimed to verify the effect of feeding protocol on nutrition therapy and clinical outcome in critically ill patients.

4.2 General study setting

Clinical interventional study performed in 97 different ICUs across China with different clinical settings, including surgical ICU, medical ICU, neurological ICU, etc. This is a cluster, randomized, controlled study. Stratified randomization will be used based on the provincial distribution of the hospitals and type of ICUs (emergency, medical, surgical, neurosurgery, and general).

4.3 Sample size

According to previous studies in China, we presumed that the 28-day mortality for mixed ICU patients was 20%, which was used as the event rate in the control group. The EN protocol was assumed to be able to reduce the mortality rate by 8% at least. The type I error was 0.05, and the statistical power was 80%. The inter-class correlation was 0.1. A total of 90 centers with 2250 subjects were required to meet the statistical power. Sample size calculation was performed by using the CRTSize

package. The full code for the calculation was: n4props (pe=0.12, pc=0.20, m=25, ICC=0.10, AR=1, alpha=0.05, power=0.80)

5. Study population

5.1 Patient recruitment

We are going to recruit approximately 2250 patients presenting to the participating ICUs into the study during an estimated one-year period. Based on the volume of all the participating centers, the aim should be able to be achieved within the study period.

5.2 Eligibility criteria

5.21 Inclusion Criteria

1. 18 years old or older;
2. Expected to stay in ICU for more than three days;

5.22 Exclusion Criteria

1. Subjects receiving EN in previous seven days;
2. Contraindications for nasogastric or nasoenteric tube placement;
3. Subjects who have already undergone percutaneous endoscopic jejunostomy (PEJ), percutaneous endoscopic gastrostomy (PEG), and surgical jejunostomy;
4. Age younger than 18 years old;
5. Women who are pregnant or undergo breastfeeding;
6. Burn patients;

6.Randomization and assignment of interventions

6.1 Sequence generation

The R language will be used for randomization for this study. All the participating centers will be stratified according to the provincial/state distribution of the hospitals and type of ICUs (emergency, medical, surgical, neurosurgery, and general). Randomization will occur in a 1:1 fashion for the participating sites within the same category with computer-generated random numbers. Allocation concealment was maintained by conducting randomization after consent to participate was obtained from the sites.

6.2 Blinding method

This study is an open-label study and no blinding method would be applied for both the participants and the investigators.

6.3. Study procedures

6.3.1 Interventional arms

Arm#1 feeding guideline group

Before EN initiation, hemodynamic parameters should be stabilized, evidenced by MAP≥65 mmHg and lactate<4mmol/L, with decreasing vasoactive dose. The gastrointestinal function will then be evaluated with the acute gastrointestinal injury (AGI) grading system. For patients with AGI of I or none, EN will be started at 25 ml/h. For patients with AGI II-III, predigested EN will be started at 10–15 ml/h. EN should be withheld for those with AGI IV. If patients are at high risk of malnutrition based on the Nutric score ≥5 (IL-6 not included), and EN cannot be initiated for reasons, PN should be started. Otherwise, PN will be withheld for 7–10 days. Patients on EN will be evaluated using a tolerance score for every 4-6 hours. The graphic feeding protocol derived from the guideline and the tolerance score are shown in

Figure 1 and Table 1, respectively. EN will be discontinued when the EN tolerance score is greater than 5 points. Adverse events will be treated and managed with a standardized protocol (Figure 2) by the treating team. In detail, EN will be discontinued in the presence of persistent abdominal pain. Physical examination and abdominal computed tomography will be ordered if deemed necessary by the treating team. If there are signs of bowel obstruction and/or ischemia, EN should be discontinued immediately. Diarrhea can be caused by enteral feeding, specific diseases and drugs, and infections, which should be considered and diagnosed by the treating team. If *Clostridium difficile* infection is identified, the patient will be treated with metronidazole or vancomycin.

Arm#2 Control group

All the participants in this group will be treated without any change to current clinical practice. No adherence to uniform protocol or guidelines will be required during the study period.

6.3.2 General management

All patients will be cared for by the local treating team in each participating ICU. In the control group, nutritional therapy would be implemented routinely at the discretion of the treating team in each participating ICU. All co-interventions will be left to the discretion of the treating clinical teams and recorded in the patient's medical record.

7. Outcome measures

7.1 Primary outcome measures

28-day mortality

7.2 Secondary outcome measures

1. Duration of mechanical ventilation at 28 days;

2. New nosocomial infection;
3. Proportion of patients receive nutrition therapy (EN/PN);
4. Mean energy delivery of EN;
5. Mean energy delivery of PN;
6. Time to start nutrition therapy (EN/PN);
7. EN tolerance score;
8. Days requiring prokinetic agents;
9. New-onset organ failure;

8. Data management, analysis, and statistics

8.1 Data collection

All the data that is necessary to define baseline patient characteristics, the implementation of the feeding protocol and control therapies, potential confounding co-interventions, and outcomes will be collected.

The primary investigator of each center will be responsible for the patient's enrollment and data input. A group of statisticians will be accountable for the pre-definition of statistical analysis and subgroup analysis.

A web-based electrical database will be used for data collection and storage. All data will be input by the site primary investigator or nominated investigator (less than two for each participating center) approved by the primary investigator. Training for data entry will be performed by the supplier of the electrical database and the sponsor of the NEED trial.

8.2 Participant timeline

All patients enrolled will be followed until either 28 days after enrollment or death, depending on which comes first. Follow-up will be restricted to information regarding the vital status and other related study clinical outcomes. Follow-up will be conducted by the study staff through either direct contact with the patient or their next of kin.

Patients who withdraw from the study for any reason will also be followed up according to the study follow-up schedule and analyzed on an intention-to-treat principle unless they withdraw from data collection and use.

8.3 Basic principle of analysis

The reporting and presentation of this trial will follow the CONSORT guidelines for cluster-randomized trials [14], with the primary comparative analysis being conducted on an intention-to-treat basis.

Descriptive statistics will be used to assess any marked baseline differences in demographics or outcome measures between the two groups, taking clustering into account. Comparisons of binary outcomes will be expressed as relative difference with 95% confidence intervals and comparisons of continuous outcomes as mean differences together with 95% confidence intervals. Between-group comparisons will be made using a generalized linear mixed-model (weighted by clusters).

All analyses will account for clustering to ensure correct type I error rates and confidence intervals. Our cluster-randomized trial will be analyzed at the individual level and use a generalized linear mixed model to account for clustering among patients in the same cluster.

Based on the principle of intention to treat (ITT), full-analysis set (FAS) will be performed on all the randomized subjects. FAS will be used for the analysis of baseline characteristics and main therapeutic interventions.

Two-sided 5% significance levels will be used to identify statistically significant results. All confidence intervals reported will be 95% confidence intervals. All p-values and estimates of change will be calculated at the individual level.

8.4 Interim analysis

A formal interim analysis will be performed at study mid-recruitment to ensure the safety of the participants. The DSMB will meet and determine whether the trial should continue.

9.Ethics and dissemination

This study has been approved by the ethics committee of the Jinling Hospital. Ethics approval of each participating center is required before initiation of enrollment.

9.1 Ethical issues of this study

The major ethical considerations include:

1. Some potential participants are unable to give consent for themselves;
2. The nutritional therapy in the control arm will be conventional.

This study has been approved by the ethics committee of the Jinling Hospital, Nanjing University, which is the sponsor of the NEED trial. Ethics approval of each participating center is required before initiation of enrollment.

9.2 Potential risks and benefits

As the feeding guideline used in this study has been tested in a pilot study without significant adverse effects, the application of this protocol should be largely safe. No specific monetary compensation is available for each participant in this study.

9.3 Consent and confidentiality

Informed consent is required for each participant of this study, either signed by the patient himself or next of kin. All the data stored in the electronic database are de-identified to guarantee patients' privacy.

9.4 Dissemination policy

All the primary investigators of the participating ICUs and the sponsor will have full access to the data after the conclusion of the study. Anyone who wants to do a

post-hoc analysis needs to submit a formal writing proposal to the *writing and publication committee*. Only approved author can have access to the database.

10. Safety issues and monitoring

10.1 Data and safety monitoring board

Data and safety monitoring board (DSMB) is an independent group of experts that offers advice during the implementation of the study. The DSMB can recommend that a trial be stopped early because of concerns about participant safety.

10.2 Adverse events

Adverse events (AEs) are defined in accordance with the National Cancer Institute-Common Terminology Criteria for Adverse Events (National Cancer Institute-Common Terminology Criteria for Adverse Events) as any untoward medical occurrence in a patient or clinical investigation subject administered an investigational intervention and which does not necessarily have to have a causal relationship with this treatment.

It is recognized that the patient population admitted to ICUs will experience a number of common aberrations in laboratory values, signs, and symptoms due to the severity of the underlying disease and the impact of standard therapies. These will not necessarily constitute an adverse event unless they require significant intervention or are considered to be of concern in the investigator's clinical judgment.

In all cases, the condition or disease underlying the symptom, sign or laboratory value should be reported, e.g. renal failure rather than hyperkalemia, and agitation rather than self-extubation.

10.3 Serious adverse events

SAEs are defined when any untoward medical occurrence that:

1.Results in death

2.Is life-threatening

3.Requires inpatient hospitalization or prolongation of existing hospitalization

4. Results in persistent or significant disability/incapacity

5. Is a congenital anomaly/birth defect

6. Is an important medical event that may require intervention to prevent one of the previously listed outcomes.

In this study, all SAEs will be reported regardless of suspected causality.

10.4 Monitoring of potential adverse events

The DSMB will be responsible for overseeing all subjects' safety and monitoring total mortality and serious adverse events. All serious adverse events occurring during the trial will be reported to the Coordinating and data management center (CDMC) within 48 hours. Minimum information to report will include:

1. Initials of the patient and study number

2. Course and nature of the event

3. An investigator's opinion of the relationship between study involvement and the event (unrelated, possibly, probably or definitely related)

4. Whether treatment is required and what treatment was applied

The contact information for CDMC

Telephone number

+86 80860007

+86 80863073

Mobile number of the primary investigator:
+86 13951839654

11.References

1. Stuani Franzosi O, Delfino von Frankenberg A, Loss SH, Silva Leite Nunes D, Rios Vieira SR. Underfeeding versus full enteral feeding in critically ill patients with acute respiratory failure: a systematic review with meta-analysis of randomized controlled trials. Nutr Hosp. 2017; 34: 19–29.
2. Hofman Z, Swinkels S, van Zanten ARH. Glutamine, fish oil and antioxidants in critical illness: MetaPlus trial post hoc safety analysis. Ann Intensive Care. Springer Paris; 2016; 6: 119.
3. Heyland DK, Dhaliwal R, Wang M, Day AG. The prevalence of iatrogenic underfeeding in the nutritionally "at-risk" critically ill patient: Results of an international, multicenter, prospective study. Clin Nutr. Elsevier; 2015; 34: 659–666.
4. McClave SA, Taylor BE, Martindale RG, Warren MM, Johnson DR, Braunschweig C, et al. Guidelines for the Provision and Assessment of Nutrition Support Therapy in the Adult Critically Ill Patient: Society of Critical Care Medicine (SCCM) and American Society for Parenteral and Enteral Nutrition (A.S.P.E.N.). Vol. 40, JPEN. Journal of parenteral and enteral nutrition. 2016. pp. 159–211.
5. Gramlich L, Kichian K, Pinilla J, Rodych NJ, Dhaliwal R, Heyland DK. Does enteral nutrition compared to parenteral nutrition result in better outcomes in critically ill adult patients? A systematic review of the literature. Nutrition. Elsevier; 2004 Oct;20(10):843–8.
6. Elke G, van Zanten ARH, Lemieux M, McCall M, Jeejeebhoy KN, Kott M, et al. Enteral versus parenteral nutrition in critically ill patients: an updated systematic review and meta-analysis of randomized controlled trials. Crit Care. BioMed Central; 2016 Apr 29;20(1):117.
7. Heyland DK, Dhaliwal R, Drover JW, Gramlich L, Dodek P, Canadian Critical Care Clinical Practice Guidelines Committee. Canadian clinical practice guidelines for nutrition support in mechanically ventilated, critically ill adult

patients. Vol. 27, JPEN. Journal of parenteral and enteral nutrition. SAGE PublicationsSage UK: London, England; 2003. pp. 355–73.

8. Padar M, Uusvel G, Starkopf L, Starkopf J, Reintam Blaser A. Implementation of enteral feeding protocol in an intensive care unit: Before-and-after study. World J Crit Care Med. 2017; 6: 56–64.
9. Cunningham CA, Gervais LB, Mazurak VC, Anand V, Garros D, Crick K, et al. Adherence to a NurseDriven Feeding Protocol in a Pediatric Intensive Care Unit. JPEN J Parenter Enteral Nutr. SAGE PublicationsSage CA: Los Angeles, CA; 2017; 170: 148607117692751.
10. Ventura AMC, Waitzberg DL. Enteral nutrition protocols for critically ill patients: are they necessary? Nutr Clin Pract. SAGE PublicationsSage CA: Los Angeles, CA; 2015; 30: 351–362.
11. Mehta NM, Bechard LJ, Cahill N, Wang M, Day A, Duggan CP, et al. Nutritional practices and their relationship to clinical outcomes in critically ill children—an international multicenter cohort study*. Crit Care Med. 2012; 40: 2204–2211..
12. Li Q, Zhang Z, Xie B, Ji X, Lu J, Jiang R, et al. Effectiveness of enteral feeding protocol on clinical outcomes in critically ill patients: A before and after study. Brakenridge S, editor. PLoS ONE. Public Library of Science; 2017;12(8):e0182393.
13. Xing J, Zhang Z, Ke L, Zhou J, Qin B, Liang H, Chen X, Liu W, Liu Z, Ai Y et al: Enteral nutrition feeding in Chinese intensive care units: a cross-sectional study involving 116 hospitals. Crit Care 2018, 22(1):229.
14. Hemming, K.; Taljaard, M.; McKenzie, J. E.; Hooper, R.; Copas, A.; Thompson, J. A.; Dixon-Woods, M.; Aldcroft, A.; Doussau, A.; Grayling, M.; Kristunas, C.; Goldstein, C. E.; Campbell, M. K.; Girling, A.; Eldridge, S.; Campbell, M. J.; Lilford, R. J.; Weijer, C.; Forbes, A. B.; Grimshaw, J. M., Reporting of stepped wedge cluster randomised trials: extension of the CONSORT 2010 statement with explanation and elaboration. BMJ 2018, 363, k1614.

Figure 1: Feeding guideline

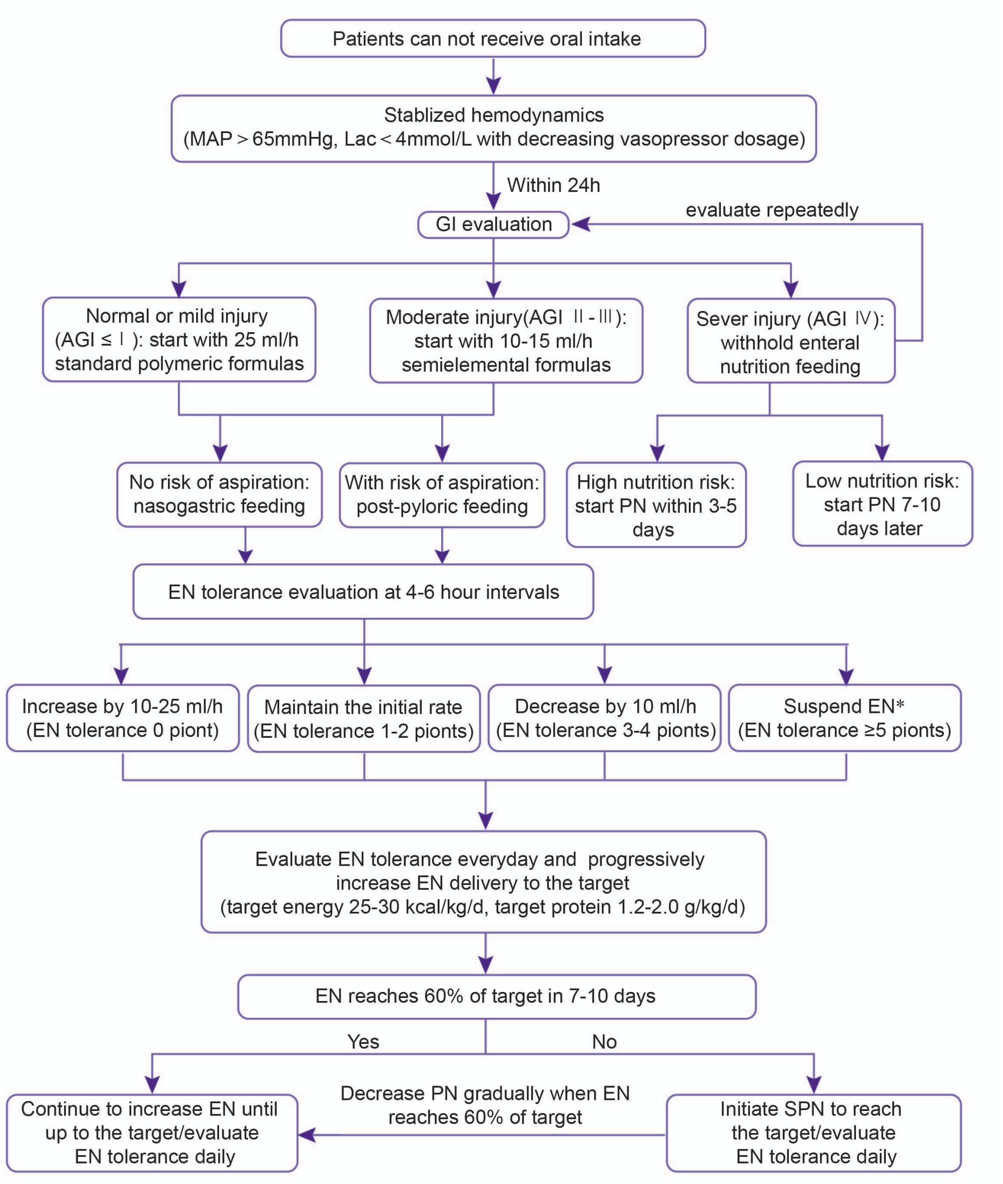

Figure 2: Protocols for the management of adverse events

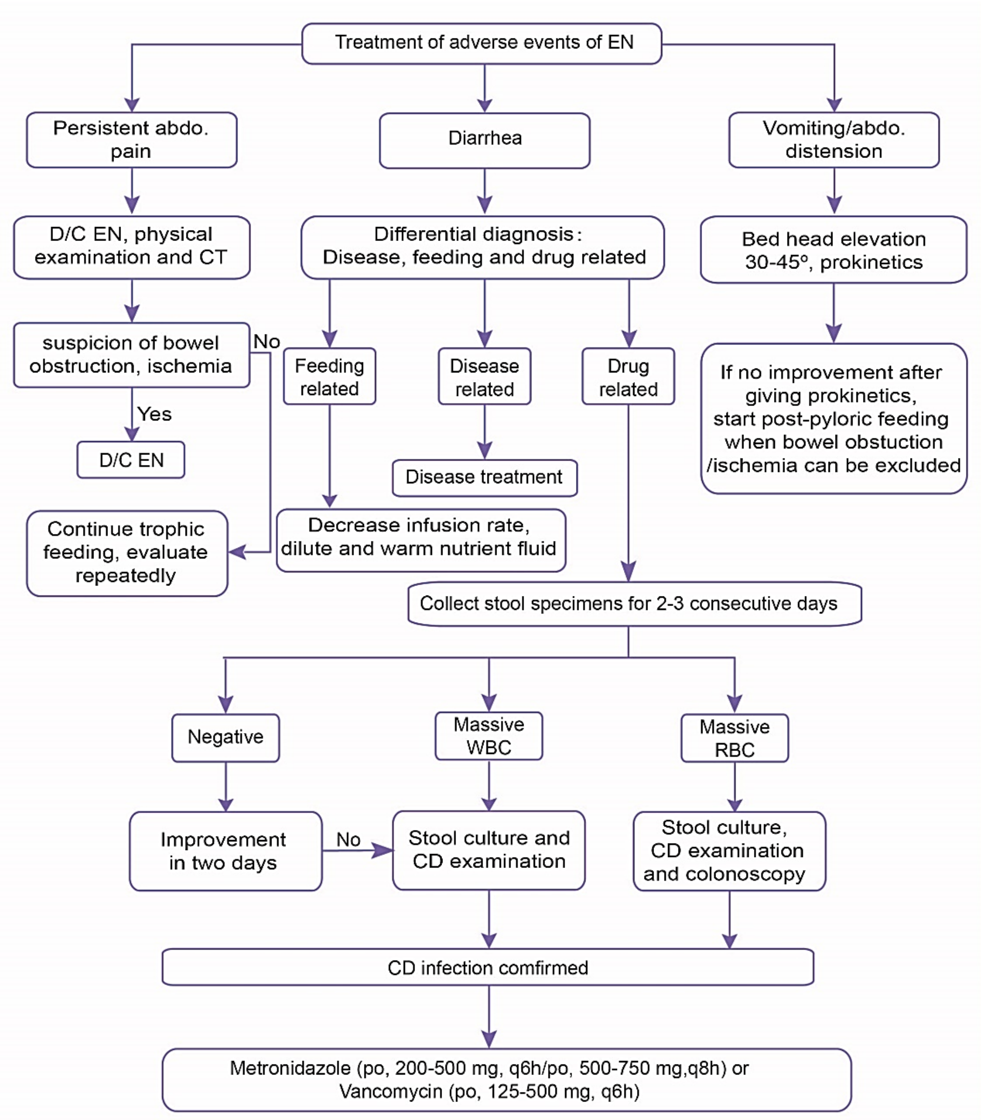

Table 1: EN tolerance score

Points	0	1	2	5
Abdominal distension/pain	None	Mild distension and no distension	Moderate distension **OR** IAP 15~20mmHg **OR** Spontaneous resolution of abdominal pain	Severe distension **OR** IAP>20mmHg **OR** No spontaneous resolution of abdominal pain
Nausea/vomiting	None **OR** Continuous gastric decompression without symptom	Nausea but no vomiting	Nausea and vomiting without need for decompression **OR** 250ml≦ GRV<500ml	Vomiting requiring gastric decompression **OR** GRV≧500ml
Diarrhea	None	Loose stools≧3 times/day with 250≦ volume<500 ml	Loose stools≧3 times/day with 500≦ volume<1500ml	Loose stools≧3 times/day with volume≧1500ml

Total score= Abdominal distension/pain + Nausea/vomiting + Diarrhea

0-2 points: continue enteral nutrition, increase or maintain initial speed, symptomatic treatment;

3-4 points: continue enteral nutrition, slow down the speed, reevaluate EN tolerance after 2h;

≥ 5 points: suspend enteral nutrition, reevaluate or replace the infusion route;

Appendix 1. Participant of ICUs

**Participant center**	**ICU type**
Anhui Provincial Hospital	General ICU
Yijishan Hospital of Wannan Medical College	General ICU
Anhui Medical University Second Affiliated Hospital	General ICU
The First Affiliated Hospital of Anhui Medical University	General ICU
First Affiliated Hospital of Fujian Medical University	General ICU
The People’s Hospital of Fujian Province	General ICU
Union Hospital of Fujian Medical University	General ICU
Fujian Provincial Hospital	Surgical ICU
First People's Hospital of Foshan	General ICU
General Hospital of Southern Theatre Command	Emergency ICU
Guangzhou First People's Hospital	General ICU
The Sixth Affiliated Hospital, Sun Yat-Sen University	General ICU
Huazhong University of Science and Technology Union Shenzhen Hospital	General ICU
Peking University Shenzhen Hospital	General ICU
General ICU, Jinan University First Affiliated Hospital	Surgical ICU
Southern Medical University Zhujiang Hospital	General ICU
Guangdong Second Traditional Chinese Medicine Hospital	General ICU
The Second People’s Hospital of Shenzhen	Medical ICU
First Affiliated Hospital of Guangzhou Medical University	General ICU
Jinan University First Affiliated Hospital	Neurosurgical ICU
Shenzhen People's Hospital	General ICU
Guangdong Provincial People's Hospital	General ICU
Shantou University Medical College First Affiliated Hospital	General ICU
Sun Yat-sen Memorial Hospital, Sun Yat-sen University	General ICU
The First Affiliated Hospital, Sun Yat-sen University	Surgical ICU
Zhongshan People's Hospital	General ICU
the First Affiliated Hospital of Guangzhou University of Chinese Medicine	General ICU
People's Hospital of Guangxi Zhuang Autonomous Region	Medical ICU
North China University of Science and Technology Affiliated Hospital	General ICU
Tangshan Gongren Hospital	General ICU
Hebei Medical University Second Affiliated Hospital	General ICU
Hebei Medical University Third Affiliated Hospital	General ICU
Luoyang Central Hospital Affiliated to Zhengzhou University	General ICU
Zhengzhou University First Affiliated Hospital	General ICU
Henan Provincial People's Hospital	General ICU
The Second Affiliated Hospital of Harbin Medical University	General ICU
The Fourth Hospital of Medical University	General ICU
First People's Hospital of Yichang	General ICU
Union Hospital Affiliated to Tongji Medical College of Huazhong University of Science and Technology	General ICU
Yichang Central People's Hospital	General ICU
Wuhan General Hospital of Guangzhou Military Region	General ICU

Hubei Provincial People's Hospital	General ICU
Zhongnan Hospital of Wuhan University	General ICU
The Third Xiangya Hospital of Central South University	General ICU
Xiangya Hospital Central South University	General ICU
The Second Xiangya Hospital of Central South University	General ICU
Changsha Central Hospital	General ICU
Hunan Provincial People's Hospital	General ICU
Suzhou Municipal Hospital	General ICU
Changzhou No.2 People's Hospital affiliated to Nanjing Medical University	General ICU
Wuxi People's Hospital	General ICU
Benq Medical Center	General ICU
Jinling Hospital	Surgical ICU
Yancheng First People's Hospital	General ICU
First Affiliated Hospital of Soochow University	Emergency ICU
Jiangxi Provincial People's Hospital	General ICU
Nanchang University Second Affiliated Hospital	General ICU
The First Affiliated Hospital of Nanchang University	General ICU
The General Hospital of Shenyang Military	Emergency ICU
China Medical University Second Affiliated Hospital	General ICU
The Second Hospital of Dalian Medical University	General ICU
Affiliated Hospital of Inner Mongolia Medical College	General ICU
Inner Mongolia People's Hospital	General ICU
Qindao University Medical College Affiliated Yantai Yuhuangding Hospital	General ICU
Zibo Central Hospital	General ICU
Linyi City People Hospital	General ICU
Jining First People's Hospital	General ICU
Qindao University Medical College Affiliated Hospital	General ICU
Qingdao Municipal Hospital Group	General ICU
Qindao University Medical College Affiliated Hospital	General ICU
No.971 hospital of People’s Liberation Army Navy	General ICU
Tai'an City Central Hospital	General ICU
Qilu Hospital, Shandong University	General ICU
Yantai Mountain Hospital	General ICU
Jining Medical College Affiliated Hospital	General ICU
Qindao University Medical College Affiliated Yantai Yuhuangding Hospital	Neurosurgical ICU
Shanxi Provincial People's Hospital	General ICU
Shanxi Medical University First Affiliated Hospital	General ICU
Shanxi Bethune Hospital	General ICU
First Affiliated Hospital of Xi'an Jiao Tong University	General ICU
Xijing Hospital	General ICU
Shaanxi Provincial People's Hospital	General ICU
Chengdu University of Traditional Chinese Medicine Affiliated Hospital	General ICU
Sichuan Provincial People's Hospital	General ICU
Dazhou Central Hospital	General ICU

Tianjing Hospital of Integration of Chinese and Western Medicine	General ICU
Tianjin Union Medical Center	General ICU
The First Affiliated Hospital of Xinjiang Medical University	General ICU
General Hospital of Xinjiang Military Command	General ICU
First People's Hospital of Kunming	General ICU
First People's Hospital of Yunnan	General ICU
People's Hospital of Yuxi City	General ICU
Kuming Medical University First Affiliated Hospital	Emergency ICU
Zhejiang University School of Medicine Sir Run Run Shaw Hospital	General ICU
Daping Hospital, Army Medical University	General ICU
The Second Affiliated Hospital of Chongqing Medical University	General ICU
Chongqing Medical University First Affiliated Hospital	General ICU

N.E.D.D

EffectiveNess of a feEding protocol on nutritional thErapy and clinical outcomes in critically ill patients: a multi-center, cluster-ranDomized, parallel-controlled trial

Protocol Ver. 2.0 dated 07. Feb. 2018

Primary Investigator

Professor. Weiqin Li

Director of surgical intensive care unit, Jinling Hospital, Nanjing University

305 East Zhongshan Rd,210002

Nanjing, Jiangsu province, China

Email: ctgchina@medbit.cn

Phone: +8680860007

Fax: +8680863073

Coordinating Centre

Chinese Critical Care Nutrition Trials Group

Nanjing, Jiangsu province, China

Phone: +8680325431

Table of contents

1.Abrreviations 31
2.Study Administrative information 32
2.1 Steering and management committee 32
2.2 Coordinating and data management center 35
2.3 Writing and publication committee 35
2.4 Data and safety monitoring board 36
2.5 Registration 36
2.6 Funding 36
3. Background and rationale 37
3.1 The role of nutritional therapy in critical care 37
3.2 The rationale for conducting this study 37
4. Study Design 38
4.1 Aim of the study 38
4.2 General study setting 38
4.3 Sample size 39
5. Study population 39
5.1 Patient recruitment 39
5.2 Eligibility criteria 39
6.Randomization and assignment of interventions 40
6.1 Patients screening procedures 40
6.2 Recruitment 40
6.3 Randomization procedures 41
6.4. Study procedures 41
7. Outcome measures 42
7.1 Primary outcome measures 42
7.2 Secondary outcome measures 42
8. Data management, analysis, and statistics 43
8.1 Data collection 43
8.2 Participant timeline 44
8.3 Basic principle of analysis 44
8.4 Interim analysis 45
9.Ethics and dissemination 45
9.1 Ethical issues of this study 45
9.2 Potential risks and benefits 45
9.3 Consent and confidentiality 46
9.4 Dissemination policy 46
10. Safety issues and monitoring 46
10.1 Data and safety monitoring board 46
10.2 Adverse events 46
10.3 Serious adverse events 47
10.4 Monitoring of potential adverse events 47
11.References 48

Figure 1: Feeding guideline 51
Figure 2: Protocols for the management of adverse events 52
Table 1: EN tolerance score 53
Appendix 1. Participant of ICUs.. 54

1.Abrreviations

CDMC	Coordinating and Data Management Center
DSMB	Data and Safety Monitoring Board
SAE	Serious Adverse Events
ICU	Intensive Care Unit
PN	Parenteral Nutrition
EN	Enteral Nutrition
SOFA	Sequential Organ Failure Assessment
MAP	Mean Artery Pressure
CPAP	Continuous Positive Airway Pressure
NIPPV	Non-Invasive Positive Pressure Ventilation
NOK	Next Of Kin
ITT	Intention-to-treat
FAS	Full-Analysis Set
AEs	Adverse Events
AGI	Acute Gastrointestinal injury
CRP	C-reaction protein
RRT	Renal Replacement Therapy
MV	Mechanical Ventilation

2.Study Administrative information

2.1 Steering and management committee

The steering and management committee is responsible for the approval of the full protocol, database, and its related methods. The members of the committee will also oversee the implementation of the study and play an advisory role.

Members of the steering committee are listed below

Name	Email
Prof. Weiqin Li (Primary investigator)	ctgchina@medbit.cn
Prof. Zhihui Tong	njzyantol@hotmail.com
Prof. Lu Ke	ctgkelu@nju.edu.cn
Dr. Zhongheng Zhang	zh_zhang1984@zju.edu.cn
Juan Xing	lasaison@126.com
Lixin Zhou	drzhoulixin@gmail.com
Dongpo Jiang	66dbjiang@163.com
Qindong Shi	shiqindong@163.com
Jiandong Lin	linjd01680067@sina.com
Jun Liu	liujunphd@sina.cn
Aibin Cheng	myfyicu@126.com
Yafeng Liang	13954551363@163.com
Peiyang Gao	GaoPY930@126.com
Junli Sun	zxyysicu@163.com
Wenming Liu	lwmicu@163.com
Zhenyu Yang	Yang_19840214@163.com
Rumin Zhang	sdzhangrm@126.com
Weixing	xy3yyxw@csu.edu.cn
An Zhang	zhangan@hospital.cqmu.edu.cn
Zhigang Zhou	ynkmzzg@163.com
Tingfa Zhou	zhoutingfa66@126.com
Yang Liu	13933358101@139.com
Fei Tong	tongfei168@163.com

Qiuhui Wang	qiuhui4403@sina.com
Aijun Pan	aijunpan@fsyy.ustc.edu.cn
Xiaobo Huang	814129752@qq.com
Chuming Fan	fanchuming@sina.com
Weihua Lu	lwh683@126.com
Dongwu Shi	shidw1@163.com
Lei Wang	413060682@163.com
Wei Li	sicu.lw@163.com
Liming Gu	glm39887@126.com
Yingguang Xie	zzyxkx@163.com
Rongqing Sun	rongqing.sun@126.com
Feng Guo	zjguofeng@aliyun.com
Lin Han	xhan0507@sina.com
Lihua Zhou	xiulika@163.com
Xiangde Zheng	zxdfg123@163.com
Feng Shan	mountain1963@163.com
Jianbo Liu	qicaixia2007@126.com
Yuhang Ai	ayhicu1978@sina.com
Yan Qu	qdquyan@aliyun.com
Liandi Li	liliandi75@sina.com
Hailing Li	lihailing608@163.com
Zhiguo Pan	gzpzg@126.com
Donglin Xu	syicuxdl@126.com
Zhiqiang Zou	396746023@qq.com
Yan Gao	Gaoyan1515@163.com
Chunli Yang	646233247@qq.com
Qiuye Kou	kouqiuye@126.com
Xijing Zhang	zhangxj918@163.com
Jinglan Wu	doctorwu99@126.com
Chuanyun Qian	qianchuanyun@126.com
Weixing Zhang	zhangwx@hotmail.com
Minjie Zhang	hychen427@163.com
Yuan Zong	chuchu20040317@qq.com

Bingyu Qin	nicolasby@126.com
Fusen Zhang	13793812109@126.com
Zhe Zhai	zhaizhe0357@163.com
Yun Sun	sunyun15@163.com
Ping Chang	changp963@163.com
Bo Yu	yuboicu@csu.edu.cn
Min Yu	ycyyicuyumin@163.com
Shiying Yuan	yuan_shiying@163.com
Yijun Deng	dengyj2003@163.com
Liyun Zhao	403756433@qq.com
Bin Zang	zangb@sj-hospital.org
Yuanfei Li	156636305@qq.com
Fachun Zhou	zfc88@126.com
Xiaomei Chen	chenxm008@163.com
Min Shao	shaominmail @yahoo.com
Weidong Wu	13503545010@163.com
Ming Wu	Boshiyy@126.com
Zhaohui Zhang	1410956890@qq.com
Yimin Li	dryiminli@vip.163.com
Qiang Guo	ggqq7910@163.com
Zhiyong Wang	18533112993@126.com
Yuanqi Gong	760225787@qq.com
Yunlin Song	17929294@qq.com
Kejian Qian	2682244275@qq.com
Yongjian Feng	horse1980@163.com
Baocai Fu	kailiaokailiao@sina.com
Xueyan Liu	1736574753@qq.com
Zhiping Li	354855270@qq.com
Chuanyong Gong	chuanyonggong@163.com
Cheng Sun	suncheng2008@139.com
Jian Yu	yujian0427@163.com
Zhongzhi Tang	Jzktzz_wh2014@163.com
Linxi Huang	13502733122@126.com
Biao Ma	305058382@qq.com

Zhijie He	805545775@qq.com
Qingshan Zhou	white1962@qq.com
Rongguo Yu	garyyrg@yahoo.com

2.2 Coordinating and data management center

Coordinating and data management centers (CDMC) was organized before the implementation of the current study. They are responsible for day to day management of the trial, assistance for ethic application in each center, protocol and case report form design, online database design and maintenance, protocol and case report form design, online database design and maintenance, protocol training for the participating centers, enrollment, data entry and quality control, severe adverse event monitor and notification and data analysis. The CDMC plans to meet before enrollment, three months after initial enrollment, and six months after initial enrollment to ensure qualified data entry.

Members of CDMC are listed below

Prof. Zhihui Tong

Prof. Lu Ke

Dr. Jing Zhou

Mr. Yafei Yan

Dr. Juan Xing

Dr. Jiajia Lin

Ms. Yan Chen

2.3 Writing and publication committee

The writing and publication committee is responsible for drafting the manuscript and submission of the manuscript to adequate journals. The Writing and publication committee will also decide on the authorship of this study. After the conclusion of this study, all participating centers are welcome to submit proposals for post-hoc analysis

to the writing and publication committee is responsible for reviewing and rating all the proposals for further analysis.

Members are listed below

Prof. Weiqin Li

Dr. Lu Ke

Dr. Zhongheng Zhang

Ms. Jiajia Lin

2.4 Data and safety monitoring board

Data and safety monitoring board (DSMB) is an independent group of experts that offers advice during the implementation of the study. The DSMB can recommend that a trial be stopped early because of concerns about participant safety.

Members of DSMB are listed below

Prof. Yuxiu Liu

Dr. Mengjie Lu

Prof. Wenkui Yu

Prof. Qiang Li

2.5 Registration

The NEED trial was registered on the ISRCTN registry (ISRCTN12233792) before commencement of the study.

2.6 Funding

The study was funded partly by the Key Research and Development Program Foundation of Jiangsu Province of China (No. BE2015685) and partly by the Nutricia, Wuxi, China.

3. Background and rationale

3.1 The role of nutritional therapy in critical care

In intensive care unit (ICU), nutritional therapy is one of the most crucial treatments for critically ill patients, which may significantly influence the clinical outcomes [1,2]. There is a large body of evidence showing that malnutrition is associated with a significantly increased risk of death. Besides, ICU patients are prone to suffer from underfeeding, which could further exacerbate the existing gap between energy demand and intake [3]. The route of nutrition delivery is another important issue when starting nutrition therapy for ICU patients. Enteral feeding has been repeatedly proven to be superior to parenteral nutrition (PN) with respect to the outcomes such as nosocomial infection, mortality, and also medical resource utilization [4-7].

As nutritional therapy involves a battery of interventions and procedures, a variety of clinical practice guidelines have been published to standardize nutritional therapy. However, it was reported that the adherence to these guidelines was suboptimal so that the clinical practices of feeding vary substantially across different regions and hospitals. Moreover, it remains controversial whether a feeding guideline could improve the adherence to current international guidelines and therefore benefit the patients in terms of clinical outcomes [8-11].

3.2 The rationale for conducting this study

China has the world's largest population of critically ill patients, and nutritional therapy varies massively. Previous small studies have shown that the proportion of EN was as low as 40% on the second day after ICU admission, which could be potentially improved with the implementation of a feeding guideline [12]. Our previous cross-sectional study showed that the proportions of subjects starting EN within 24, 48 and 72 h after ICU entry was 24.8% (84/352), 32.7% (150/459) and

40.0% (200/541), suggesting the unsatisfying adherence to the current international guidelines and huge space for improvement [13]. Accordingly, we developed an evidence-based, multifaceted, and practical feeding guideline and related educational materials to overcome barriers and enhance nutritional therapy performance. A before-and-after pilot study was conducted in 2016 to test the feasibility of the feeding guideline [12]. The results showed that it significantly changed the practice but failed to provide clinically meaningful benefits, warranting a large-scale study to verify the findings further. Considering the possibility that patients assigned to the control group may receive nutritional therapy guided or partly guided by the feeding guideline if it was simultaneously operational at the same institute, randomization was performed at ICU-level rather than individual patient level. A cluster-randomized method can effectively control the confounding factors between groups to obtain more reliable conclusions.

4. Study Design

4.1 Aim of the study

We aimed to assess the effect of an evidence-based feeding guideline on nutritional therapy and clinical outcomes in critically ill patients. The hypothesis is that a feeding guideline, compared to routine clinical practice, could reduce 28-day mortality in critically ill patients. The null hypothesis is there is no difference in terms of 28-day mortality in patients assigned to either feeding guideline group or routine group.

4.2 General study setting

Clinical interventional study performed in 97 different ICUs across China with different clinical settings, including surgical ICU, medical ICU, neurological ICU, etc. This is a cluster, randomized, controlled study. Stratified randomization will be used

based on the provincial distribution of the hospitals and type of ICUs (emergency, medical, surgical, neurosurgery, and general).

4.3 Sample size

According to previous studies in China, we presumed that the 28-day mortality for mixed ICU patients was 20%, which was used as the event rate in the control group. The EN protocol was assumed to be able to reduce the mortality rate by 8% at least. The type I error was 0.05, and the statistical power was 80%. The inter-class correlation was 0.1. A total of 90 centers with 2250 subjects were required to meet the statistical power. Sample size calculation was performed by using the CRTSize package. The full code for the calculation was: n4props (pe=0.12, pc=0.20, m=25, ICC=0.10, AR=1, alpha=0.05, power=0.80)

5. Study population

5.1 Patient recruitment

We are going to recruit approximately 2250 patients presenting to the participating ICUs into the study during an estimated one-year period. Based on the volume of all the participating centers, the aim should be able to be achieved within the study period.

5.2 Eligibility criteria

5.21 Inclusion Criteria

1. Informed consent form obtained from the patient or next of kin;
2. 18 years old or older;
3. Within 24h of ICU admission;
4. With one or more organ failure (SOFA for any individual organ system≥2);

5. Expected to stay in ICU for more than seven days;
6. Oral diet is not likely to be restored within three days.

5.22 Exclusion Criteria

1. Patients receive EN in the past three days before admission;
2. Patients receiving palliative treatment or expected to die within 48 hours;
3. Women in pregnancy;
4. Long-term use of steroids or immunosuppressive agents;
5. Patients with malignant diseases receiving radiotherapy or chemotherapy.

6.Randomization and assignment of interventions

6.1 Patients screening procedures

All patients presenting to the ICUs of each participating hospital will be assessed by the treating physician and receive medical care immediately according to the current best clinical practice. The treating clinician team will be responsible for identifying potential patients and contact the NEED coordinator or a member of the NEED study team who will assess the patients for eligibility into the study. Screening tools will be provided, and a screening log will be kept.

Each participating center will be led by a NEED project leader (center primary investigator) and a site coordinator. The former will be responsible for implementing the feeding guideline if assigned to the study group and monitor the NEED trial. The latter will be responsible for daily screening and data management.

6.2 Recruitment

All patients admitted to the participating ICUs will be considered for enrollment and assessed for inclusion and exclusion criteria. Where possible, informed consent will be obtained from the patient or next of kin (NOK) prior to enrollment into the study.

6.3 Randomization procedures

6.3.1 Sequence generation

The R language will be used for randomization for this study. All the participating centers will be stratified according to the provincial/state distribution of the hospitals and types of ICUs (emergency, medical, surgical, neurosurgery, and general). Randomization will occur in a 1:1 fashion for the participating sites within the same category with computer-generated random numbers. Allocation concealment was maintained by conducting randomization after consent to participate was obtained from the sites.

6.3.2 Blinding method

This study is an open-label study, and no blinding method would be applied for both the participants and the investigators.

6.4. Study procedures

6.4.1 Interventional arms

Arm#1 feeding guideline group

Before EN initiation, hemodynamic parameters should be stabilized evidenced by MAP≥65 mmHg and lactate<4mmol/L, with decreasing vasoactive dose. The gastrointestinal function will then be evaluated with the acute gastrointestinal injury (AGI) grading system. For patients with AGI of I or none, EN will be started at 25 ml/h. For patients with AGI II-III, predigested EN will be started at 10–15 ml/h. EN should be withheld for those with AGI IV. If patients are at high risk of malnutrition based on the Nutric score ≥5 (IL-6 not included), but EN cannot be initiated, PN should be started. Otherwise, PN will be withheld for 7–10 days. Patients on EN will be evaluated using a tolerance score for every 4-6 hours. The graphic feeding protocol derived from the guideline and the tolerance score are shown in Figure 1 and Table 1, respectively. EN will be discontinued when the EN tolerance score is greater than 5 points. Adverse events will be treated and managed with a standardized protocol (Figure 2) by the treating team. In detail, EN will be discontinued in the presence of

persistent abdominal pain. Physical examination and abdominal computed tomography will be ordered if deemed necessary by the treating team. If there are signs of bowel obstruction and/or ischemia, EN should be discontinued immediately. Diarrhea can be caused by enteral feeding, specific diseases and drugs, and infections, which should be considered and diagnosed by the treating team. If *Clostridium difficile* infection is identified, the patient will be treated with metronidazole or vancomycin.

Arm#2 Control group

All the participants in this group will be treated without any change to the current clinical practice. No adherence to uniform protocol or guidelines will be required during the study period.

6.4.2 General management

All patients will be cared for by the local treating team at each participating ICU. In the control group, nutritional therapy would be implemented routinely at the discretion of the treating team. All co-interventions will be left to the discretion of the treating clinical teams and recorded in the patient's medical record.

7. Outcome measures

7.1 Primary outcome measures

The primary outcome measure is all-cause mortality at day 28 after enrollment (the day of enrollment will be set as day 1).

7.2 Secondary outcome measures

1. Time to start EN
2. Time to start PN
3. Mean nutrition support days within first seven days after enrollment
4. Mean energy per day over the first seven days for patients who were fed
5. The proportion of patients never fed during the first seven days after enrollment

6. The proportion of patients received EN within two days after enrollment
7. The proportion of patients received PN within two days after enrollment
8. The proportion of fed patients within two days after enrollment
9. The proportion of patients received EN or PN within two days after enrollment
10. EN tolerance score during the first seven days after enrollment
11. Days requiring prokinetic agents within the first seven days after enrollment
12. The proportion of patients who received a post-pyloric feeding tube (patients receiving EN) within first seven days after enrollment.
13. New-onset organ failure within first seven days (respiratory, cardiovascular, renal)
14. Days requiring MV within first seven days after enrollment.
15. Incidence of new infection in ICU

8. Data management, analysis, and statistics

8.1 Data collection

All the data that is necessary to define baseline patient characteristics, the implementation of the feeding protocol and control therapies, potential confounding co-interventions, and outcomes will be collected.

The primary investigator of each center will be responsible for the patient's enrollment and data input. A group of statisticians will be accountable for the pre-definition of statistical analysis and subgroup analysis.

A web-based electrical database will be used for data collection and storage. All data will be input by the site primary investigator or nominated investigator (less than two for each participating center) approved by the primary investigator. Training for data entry will be performed by the supplier of the electrical database and the sponsor of the NEED trial.

8.2 Participant timeline

All patients enrolled will be followed until either 28 days after enrollment or death, depending on which comes first. Follow-up will be restricted to information regarding the vital status and other related study clinical outcomes. Follow-up will be conducted by the study staff through either direct contact with the patient or their next of kin. Patients who withdraw from the study for any reason will also be followed up according to the study follow-up schedule and analyzed on an intention-to-treat principle unless they withdraw from data collection and use.

8.3 Basic principle of analysis

The reporting and presentation of this trial will follow the CONSORT guidelines for cluster-randomized trials [14], with the primary comparative analysis being conducted on an intention-to-treat basis.

Descriptive statistics will be used to assess any marked baseline differences in demographics or outcome measures between the two groups, taking clustering into account. Comparisons of binary outcomes will be expressed as relative difference with 95% confidence intervals and comparisons of continuous outcomes as mean differences together with 95% confidence intervals. Between-group comparisons will be made using a generalized linear mixed-model (weighted by clusters).

All analyses will account for clustering to ensure correct type I error rates and confidence intervals. Our cluster-randomized trial will be analyzed at the individual level and use a generalized linear mixed model to account for clustering among patients in the same cluster.

Based on the principle of intention to treat (ITT), full-analysis set (FAS) will be performed on all the randomized subjects. FAS will be used for the analysis of baseline characteristics and main therapeutic interventions.

Two-sided 5% significance levels will be used to identify statistically significant results. All confidence intervals reported will be 95% confidence intervals. All p-values and estimates of change will be calculated at the individual level.

8.4 Interim analysis

A formal interim analysis will be performed at study mid-recruitment to ensure the safety of the participants. The DSMB will meet and determine whether the trial should continue.

9.Ethics and dissemination

This study has been approved by the ethics committee of the Jinling Hospital. Ethics approval of each participating center is required before initiation of enrollment.

9.1 Ethical issues of this study

The major ethical considerations include:

1. Some potential participants are unable to give consent for themselves;
2. The nutritional therapy in the control arm will be conventional.

This study has been approved by the ethics committee of the Jinling Hospital, Nanjing University, which is the sponsor of the NEED trial. Ethics approval of each participating center is required before initiation of enrollment.

9.2 Potential risks and benefits

As the feeding guideline used in this study has been tested in a pilot study without significant adverse effects, the application of this protocol should be largely safe. No specific monetary compensation is available for each participant in this study.

9.3 Consent and confidentiality

Informed consent is required for each participant of this study, either signed by the patient himself or next of kin. All the data stored in the electronic database are de-identified to guarantee patients' privacy.

9.4 Dissemination policy

All the primary investigators of the participating ICUs and the sponsor will have full access to the data after the conclusion of the study. Anyone who wants to do a post-hoc analysis needs to submit a formal writing proposal to the *writing and publication committee*. Only approved author can have access to the database.

10. Safety issues and monitoring

10.1 Data and safety monitoring board

Data and safety monitoring board (DSMB) is an independent group of experts that offers advice during the implementation of the study. The DSMB can recommend that a trial be stopped early because of concerns about participant safety.

10.2 Adverse events

Adverse events (AEs) are defined in accordance with the National Cancer Institute-Common Terminology Criteria for Adverse Events (National Cancer Institute-Common Terminology Criteria for Adverse Events) as any untoward medical occurrence in a patient or clinical investigation subject administered an investigational intervention and which does not necessarily have to have a causal relationship with this treatment.

It is recognized that the patient population admitted to ICUs will experience a number of common aberrations in laboratory values, signs, and symptoms due to the

severity of the underlying disease and the impact of standard therapies. These will not necessarily constitute an adverse event unless they require significant intervention or are considered to be of concern in the investigator's clinical judgment.

In all cases, the condition or disease underlying the symptom, sign or laboratory value should be reported, e.g. renal failure rather than hyperkalemia, and agitation rather than self-extubation.

10.3 Serious adverse events

SAEs are defined when any untoward medical occurrence that:

1.Results in death

2.Is life-threatening

3.Requires inpatient hospitalization or prolongation of existing hospitalization

4. Results in persistent or significant disability/incapacity

5. Is a congenital anomaly/birth defect

6. Is an important medical event that may require intervention to prevent one of the previously listed outcomes.

In this study, all SAEs will be reported regardless of suspected causality.

10.4 Monitoring of potential adverse events

The DSMB will be responsible for overseeing all subjects' safety and monitoring total mortality and serious adverse events. All serious adverse events occurring during the trial will be reported to the Coordinating and data management center (CDMC) within 48 hours. Minimum information to report will include:

1. Initials of the patient and study number

2. Course and nature of the event

3. An investigator's opinion of the relationship between study involvement and the event (unrelated, possibly, probably or definitely related)

4. Whether treatment is required and what treatment was applied

The contact information for CDMC

Telephone number

+86 80860007

+86 80863073

Mobile number of the primary investigator:
+86 13951839654

11.References

1. Stuani Franzosi O, Delfino von Frankenberg A, Loss SH, Silva Leite Nunes D, Rios Vieira SR. Underfeeding versus full enteral feeding in critically ill patients with acute respiratory failure: a systematic review with meta-analysis of randomized controlled trials. Nutr Hosp. 2017; 34: 19–29.
2. Hofman Z, Swinkels S, van Zanten ARH. Glutamine, fish oil and antioxidants in critical illness: MetaPlus trial post hoc safety analysis. Ann Intensive Care. Springer Paris; 2016; 6: 119.
3. Heyland DK, Dhaliwal R, Wang M, Day AG. The prevalence of iatrogenic underfeeding in the nutritionally “at-risk” critically ill patient: Results of an international, multicenter, prospective study. Clin Nutr. Elsevier; 2015; 34: 659–666.
4. McClave SA, Taylor BE, Martindale RG, Warren MM, Johnson DR, Braunschweig C, et al. Guidelines for the Provision and Assessment of Nutrition Support Therapy in the Adult Critically Ill Patient: Society of Critical Care Medicine (SCCM) and American Society for Parenteral and Enteral Nutrition (A.S.P.E.N.). Vol. 40, JPEN. Journal of parenteral and enteral nutrition. 2016. pp. 159–211.
5. Gramlich L, Kichian K, Pinilla J, Rodych NJ, Dhaliwal R, Heyland DK. Does enteral nutrition compared to parenteral nutrition result in better outcomes in

critically ill adult patients? A systematic review of the literature. Nutrition. Elsevier; 2004 Oct;20(10):843–8.

6. Elke G, van Zanten ARH, Lemieux M, McCall M, Jeejeebhoy KN, Kott M, et al. Enteral versus parenteral nutrition in critically ill patients: an updated systematic review and meta-analysis of randomized controlled trials. Crit Care. BioMed Central; 2016 Apr 29;20(1):117.
7. Heyland DK, Dhaliwal R, Drover JW, Gramlich L, Dodek P, Canadian Critical Care Clinical Practice Guidelines Committee. Canadian clinical practice guidelines for nutrition support in mechanically ventilated, critically ill adult patients. Vol. 27, JPEN. Journal of parenteral and enteral nutrition. SAGE PublicationsSage UK: London, England; 2003. pp. 355–73.
8. Padar M, Uusvel G, Starkopf L, Starkopf J, Reintam Blaser A. Implementation of enteral feeding protocol in an intensive care unit: Before-and-after study. World J Crit Care Med. 2017; 6: 56–64.
9. Cunningham CA, Gervais LB, Mazurak VC, Anand V, Garros D, Crick K, et al. Adherence to a NurseDriven Feeding Protocol in a Pediatric Intensive Care Unit. JPEN J Parenter Enteral Nutr. SAGE PublicationsSage CA: Los Angeles, CA; 2017; 170: 148607117692751.
10. Ventura AMC, Waitzberg DL. Enteral nutrition protocols for critically ill patients: are they necessary? Nutr Clin Pract. SAGE PublicationsSage CA: Los Angeles, CA; 2015; 30: 351–362.
11. Mehta NM, Bechard LJ, Cahill N, Wang M, Day A, Duggan CP, et al. Nutritional practices and their relationship to clinical outcomes in critically ill children—an international multicenter cohort study*. Crit Care Med. 2012; 40: 2204–2211..
12. Li Q, Zhang Z, Xie B, Ji X, Lu J, Jiang R, et al. Effectiveness of enteral feeding protocol on clinical outcomes in critically ill patients: A before and after study. Brakenridge S, editor. PLoS ONE. Public Library of Science; 2017;12(8):e0182393.

13. Xing J, Zhang Z, Ke L, Zhou J, Qin B, Liang H, Chen X, Liu W, Liu Z, Ai Y et al: Enteral nutrition feeding in Chinese intensive care units: a cross-sectional study involving 116 hospitals. Crit Care 2018, 22(1):229.
14. Hemming, K.; Taljaard, M.; McKenzie, J. E.; Hooper, R.; Copas, A.; Thompson, J. A.; Dixon-Woods, M.; Aldcroft, A.; Doussau, A.; Grayling, M.; Kristunas, C.; Goldstein, C. E.; Campbell, M. K.; Girling, A.; Eldridge, S.; Campbell, M. J.; Lilford, R. J.; Weijer, C.; Forbes, A. B.; Grimshaw, J. M., Reporting of stepped wedge cluster randomised trials: extension of the CONSORT 2010 statement with explanation and elaboration. BMJ 2018, 363, k1614.

Figure 1: Feeding guideline

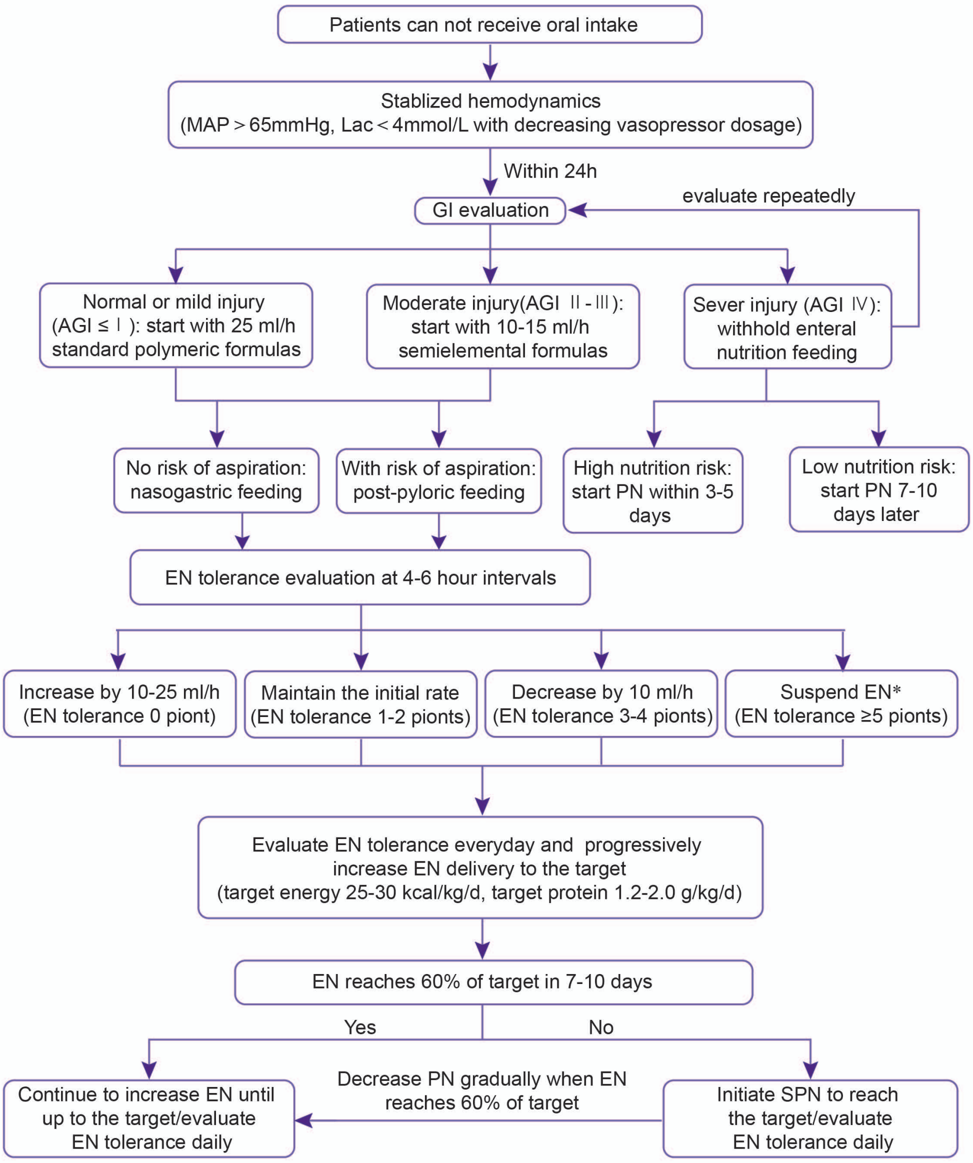

Figure 2: Protocols for the management of adverse events

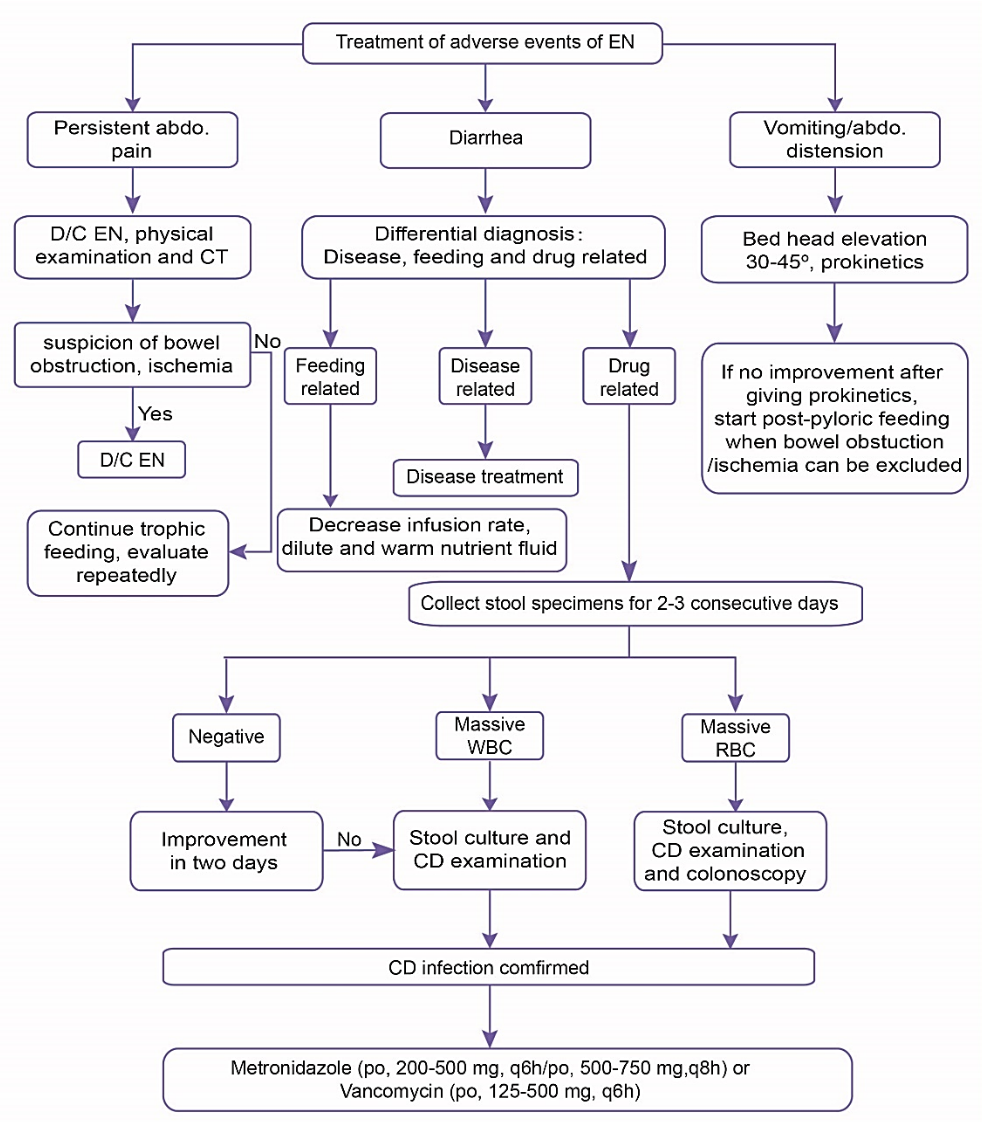

Table 1: EN tolerance score

Points	0	1	2	5
Abdominal distension/pain	None	Mild distension and no distension	Moderate distension **OR** IAP 15~20mmHg **OR** Spontaneous resolution of abdominal pain	Severe distension **OR** IAP>20mmHg **OR** No spontaneous resolution of abdominal pain
Nausea/vomiting	None **OR** Continuous gastric decompression without symptom	Nausea but no vomiting	Nausea and vomiting without need for decompression **OR** 250ml≦GRV<500ml	Vomiting requiring gastric decompression **OR** GRV≧500ml
Diarrhea	None	Loose stools≧3 times/day with 250≦volume<500 ml	Loose stools≧3 times/day with 500≦volume<1500ml	Loose stools≧3 times/day with volume≧1500ml

Total score= Abdominal distension/pain + Nausea/vomiting + Diarrhea

0-2 points: continue enteral nutrition, increase or maintain initial speed, symptomatic treatment;

3-4 points: continue enteral nutrition, slow down the speed, reevaluate EN tolerance after 2h;

≥ 5 points: suspend enteral nutrition, reevaluate or replace the infusion route;

Appendix 1. Participant of ICUs

**Participant center**	**ICU type**
Anhui Provincial Hospital	General ICU
Yijishan Hospital of Wannan Medical College	General ICU
Anhui Medical University Second Affiliated Hospital	General ICU
The First Affiliated Hospital of Anhui Medical University	General ICU
First Affiliated Hospital of Fujian Medical University	General ICU
The People’s Hospital of Fujian Province	General ICU
Union Hospital of Fujian Medical University	General ICU
Fujian Provincial Hospital	Surgical ICU
First People's Hospital of Foshan	General ICU
General Hospital of Southern Theatre Command	Emergency ICU
Guangzhou First People's Hospital	General ICU
The Sixth Affiliated Hospital, Sun Yat-Sen University	General ICU
Huazhong University of Science and Technology Union Shenzhen Hospital	General ICU
Peking University Shenzhen Hospital	General ICU
General ICU, Jinan University First Affiliated Hospital	Surgical ICU
Southern Medical University Zhujiang Hospital	General ICU
Guangdong Second Traditional Chinese Medicine Hospital	General ICU
The Second People’s Hospital of Shenzhen	Medical ICU
First Affiliated Hospital of Guangzhou Medical University	General ICU
Jinan University First Affiliated Hospital	Neurosurgical ICU
Shenzhen People's Hospital	General ICU
Guangdong Provincial People's Hospital	General ICU
Shantou University Medical College First Affiliated Hospital	General ICU
Sun Yat-sen Memorial Hospital, Sun Yat-sen University	General ICU
The First Affiliated Hospital, Sun Yat-sen University	Surgical ICU
Zhongshan People's Hospital	General ICU
the First Affiliated Hospital of Guangzhou University of Chinese Medicine	General ICU
People's Hospital of Guangxi Zhuang Autonomous Region	Medical ICU
North China University of Science and Technology Affiliated Hospital	General ICU
Tangshan Gongren Hospital	General ICU
Hebei Medical University Second Affiliated Hospital	General ICU
Hebei Medical University Third Affiliated Hospital	General ICU
Luoyang Central Hospital Affiliated to Zhengzhou University	General ICU
Zhengzhou University First Affiliated Hospital	General ICU
Henan Provincial People's Hospital	General ICU
The Second Affiliated Hospital of Harbin Medical University	General ICU
The Fourth Hospital of Medical University	General ICU
First People's Hospital of Yichang	General ICU
Union Hospital Affiliated to Tongji Medical College of Huazhong University of Science and Technology	General ICU
Yichang Central People's Hospital	General ICU
Wuhan General Hospital of Guangzhou Military Region	General ICU

Hubei Provincial People's Hospital	General ICU
Zhongnan Hospital of Wuhan University	General ICU
The Third Xiangya Hospital of Central South University	General ICU
Xiangya Hospital Central South University	General ICU
The Second Xiangya Hospital of Central South University	General ICU
Changsha Central Hospital	General ICU
Hunan Provincial People's Hospital	General ICU
Suzhou Municipal Hospital	General ICU
Changzhou No.2 People's Hospital affiliated to Nanjing Medical University	General ICU
Wuxi People's Hospital	General ICU
Benq Medical Center	General ICU
Jinling Hospital	Surgical ICU
Yancheng First People's Hospital	General ICU
First Affiliated Hospital of Soochow University	Emergency ICU
Jiangxi Provincial People's Hospital	General ICU
Nanchang University Second Affiliated Hospital	General ICU
The First Affiliated Hospital of Nanchang University	General ICU
The General Hospital of Shenyang Military	Emergency ICU
China Medical University Second Affiliated Hospital	General ICU
The Second Hospital of Dalian Medical University	General ICU
Affiliated Hospital of Inner Mongolia Medical College	General ICU
Inner Mongolia People's Hospital	General ICU
Qindao University Medical College Affiliated Yantai Yuhuangding Hospital	General ICU
Zibo Central Hospital	General ICU
Linyi City People Hospital	General ICU
Jining First People's Hospital	General ICU
Qindao University Medical College Affiliated Hospital	General ICU
Qingdao Municipal Hospital Group	General ICU
Qindao University Medical College Affiliated Hospital	General ICU
No.971 hospital of People’s Liberation Army Navy	General ICU
Tai'an City Central Hospital	General ICU
Qilu Hospital, Shandong University	General ICU
Yantai Mountain Hospital	General ICU
Jining Medical College Affiliated Hospital	General ICU
Qindao University Medical College Affiliated Yantai Yuhuangding Hospital	Neurosurgical ICU
Shanxi Provincial People's Hospital	General ICU
Shanxi Medical University First Affiliated Hospital	General ICU
Shanxi Bethune Hospital	General ICU
First Affiliated Hospital of Xi'an Jiao Tong University	General ICU
Xijing Hospital	General ICU
Shaanxi Provincial People's Hospital	General ICU
Chengdu University of Traditional Chinese Medicine Affiliated Hospital	General ICU
Sichuan Provincial People's Hospital	General ICU
Dazhou Central Hospital	General ICU

Tianjing Hospital of Integration of Chinese and Western Medicine	General ICU
Tianjin Union Medical Center	General ICU
The First Affiliated Hospital of Xinjiang Medical University	General ICU
General Hospital of Xinjiang Military Command	General ICU
First People's Hospital of Kunming	General ICU
First People's Hospital of Yunnan	General ICU
People's Hospital of Yuxi City	General ICU
Kuming Medical University First Affiliated Hospital	Emergency ICU
Zhejiang University School of Medicine Sir Run Run Shaw Hospital	General ICU
Daping Hospital, Army Medical University	General ICU
The Second Affiliated Hospital of Chongqing Medical University	General ICU
Chongqing Medical University First Affiliated Hospital	General ICU

.

N.E.D.D

EffectiveNess of a feEding guideline on nutritional thErapy and clinical outcomes in critically ill patients: a multi-center, cluster-ranDomized, parallel-controlled trial

Protocol Ver 4.0 dated 01.Jan. 2019

Primary Investigator

Professor. Weiqin Li

Director of surgical intensive care unit, Jinling Hospital, Nanjing University

305 East Zhongshan Rd,210002

Nanjing, Jiangsu province, China

Email: ctgchina@medbit.cn

Phone: +8680860007

Fax:+8680863073

Coordinating Centre

Chinese Critical Care Nutrition Trials Group

Nanjing, Jiangsu province, China

Phone: +8680325431

SUMMARY INFORMATION TYPE	SUMMARY DETAILS
**Acronym (Short Title)**	NEED
Long Title	Effectiveness of a feeding guideline on nutritional therapy and clinical outcomes in critically ill patients: a multi-center, cluster-ranDomized, parallel-controlled trial
**Version**	4.0
**Date**	01 Jan 2019
**ISRCTN #**	ISRCTN12233792
**Study Design**	A multi-center, cluster-randomized, parallel-controlled trial
**Type of Participants to be studied**	Patients aged 18 and older who are expected to stay in the ICU for more than seven days
**Setting**	97 ICUs (See the Appendix 1)
**Interventions to be Compared**	1. Intervention Group: Implementation of a feeding guideline.<br>2. Control Group: No adherence to uniform protocol and without any change to current clinical practice.
**Study Hypothesis**	The hypothesis is that a feeding guideline, compared to routine clinical practice, could reduce 28-day mortality in critically ill patients.
**Primary Outcomes Measure(s)**	All-cause mortality at day 28 after enrollment
**Secondary Outcome Measure(s)**	***# Process measures***<br>1. Time to start EN<br>2. Time to start PN<br>3. Mean nutrition support days within first seven days after enrollment<br>3.1 Mean nutritional support(either EN or PN or both) days within first seven days after enrollment<br>3.2 Mean EN support days within first seven days after enrollment<br>3.3 Mean PN support days within first s even days after enrollment<br>4. Mean energy per day over the first seven days for patients who were fed<br>4.1 Mean EN per day over the first seven days for patients who were fed<br>4.2 Mean PN per day over the first seven days for patients who were fed<br>5. The proportion of patients never fed during the first seven days after enrollment<br>6. The proportion of patients received EN within two days after enrollment<br>7. The proportion of patients received PN

	within two days after enrollment<br>8. The proportion of fed patients within two days after enrollment<br>9. The proportion of patients received EN or PN within two days after enrollment<br>10. EN tolerance score during the first seven days after enrollment<br>11. Days requiring prokinetic agents within the first seven days after enrollment<br>12. The proportion of patients who received a post-pyloric feeding tube (patients receiving EN) within first seven days after enrollment.<br>***# Organ dysfunction-related outcomes***<br>1.New-onset organ failure within first seven days<br>1.1 New-onset respiratory failure;<br>1.2 New-onset cardiovascular failure;<br>1.3 New-onset renal failure<br>2. New receipt of organ support therapy within first seven days<br>2.1 New receipt of mechanical ventilation (non-invasive included)<br>2.2 New receipt of renal replacement therapy<br>2.3 New receipt of vasoactive agents<br>2.4 Days requiring CRRT within first seven days after enrollment<br>2.5 Days requiring insulin within first seven days after enrollment<br>2.6 Days requiring MV within first seven days after enrollment<br>***# Key secondary outcomes***<br>1. Incidence of new infection in ICU<br>2. ICU-free days within 28 days
**Randomization**	Randomization at ICU-level using R language
**Number of Clusters and Participates to be studied**	97 ICUs from different hospitals<br>2250 patients
**Duration**	Three years
**Sponsor**	Jinling Hospital of Nanjing University
**Founder**	Key Research and Development Program Foundation of Jiangsu Province of China (No. BE2015685) and the Nutricia, Wuxi, China.
**Chief Investigators**	Professor. Weiqin Li

Table of contents

1.Abbreviations 62
2.Study Administrative information 63
2.1 Study timelines........ 63
2.2 Steering and management committee 63
2.3 Coordinating and data management center........ 66
2.4 Writing and publication committee 67
2.5 Data and safety monitoring board........ 68
2.6 Registration 68
2.7 Funding 68
3. Background and rationale........ 68
3.1 The role of nutritional therapy in critical care 68
3.2 The rationale for conducting this study 69
4. Study Design 70
4.1 Aims and hypotheses 70
4.2 General study setting........ 70
4.3 Sample size 70
5. Study population 71
5.1 Patient recruitment 71
5.2 Eligibility Criteria 71
6.Randomization and assignment of interventions 72
6.1 Patients screening procedures 72
6.2 Recruitment........ 72
6.3 Randomization procedures........ 72
6.4. Study procedures........ 73
7. Outcome measures 74
7.1 Primary outcome measures 74
7.2 Secondary outcome measures 74
7.3 Definition of outcomes 75
8. Data management, analysis, and statistics........ 76
8.1 Data collection 76
8.2 Participant timeline 77
8.3 The flow of ICUs and patients 77
8.4 Basic principle of analysis 77
8.5 Interim analysis........ 78
9.Ethics and dissemination........ 78
9.1 Ethical issues of this study........ 78
9.2 Potential risks and benefits 79
9.3 Consent and confidentiality 79
9.4 Dissemination policy 79
10. Safety issues and monitoring 79
10.1 Data and safety monitoring board........ 79
10.2 Adverse events 79

10.3 Serious adverse events 80
10.4 Monitoring of potential adverse events 80
11. References 81
Figure 1: Graphic Feeding Protocol 84
Figure 2: Protocols for the management of adverse events 85
Table 1: EN tolerance score 86
Appendix 1. Participant ICUs 87

1.Abbreviations

CDMC	Coordinating and Data Management Center
DSMB	Data and Safety Monitoring Board
SAE	Serious Adverse Events
ICU	Intensive Care Unit
PN	Parenteral Nutrition
EN	Enteral Nutrition
SOFA	Sequential Organ Failure Assessment
MAP	Mean Artery Pressure
CPAP	Continuous Positive Airway Pressure
NIPPV	Non-Invasive Positive Pressure Ventilation
NOK	Next Of Kin
ITT	Intention-to-treat
FAS	Full-Analysis Set
AEs	Adverse Events
AGI	Acute Gastrointestinal injury
CRP	C-reaction protein
RRT	Renal Replacement Therapy
MV	Mechanical Ventilation

2.Study Administrative information

2.1 Study timelines

December 2015	Feeding guideline drafted
April 2016	The first National Expert Symposium
May 2016	Feeding guideline and graphic protocol V1 finalized
June 2016	Communication with potential sites for research interests
May 2017	Hospital Human Research Ethics Committee (HREC) submissions
July 2017	HREC approvals obtained
August 2017	Commence vanguard phase and training in test sites
January 2018	Patient recruitment commences (all sites)
February 2019	Interim analysis
June 2019	Patient enrollment completed
July 2019	Follow-up completed
November 2019	Analysis Plan finalized
December 2019	Initiation of analysis

2.2 Steering and management committee

The steering and management committee is responsible for the approval of the full protocol, database, and its related methods. The members of the committee will also oversee the implementation of the study and play an advisory role.

Members of the steering committee are listed below

**Name**	**Email**
Prof. Weiqin Li (Primary investigator)	ctgchina@medbit.cn
Prof. Zhihui Tong	njzyantol@hotmail.com

Prof. Lu Ke	ctgkelu@nju.edu.cn
Dr. Zhongheng Zhang	zh_zhang1984@zju.edu.cn
Juan Xing	lasaison@126.com
Lixin Zhou	drzhoulixin@gmail.com
Dongpo Jiang	66dbjiang@163.com
Qindong Shi	shiqindong@163.com
Jiandong Lin	linjd01680067@sina.com
Jun Liu	liujunphd@sina.cn
Aibin Cheng	myfyicu@126.com
Yafeng Liang	13954551363@163.com
Peiyang Gao	GaoPY930@126.com
Junli Sun	zxyysicu@163.com
Wenming Liu	lwmicu@163.com
Zhenyu Yang	Yang_19840214@163.com
Rumin Zhang	sdzhangrm@126.com
Weixing	xy3yyxw@csu.edu.cn
An Zhang	zhangan@hospital.cqmu.edu.cn
Zhigang Zhou	ynkmzzg@163.com
Tingfa Zhou	zhoutingfa66@126.com
Yang Liu	13933358101@139.com
Fei Tong	tongfei168@163.com
Qiuhui Wang	qiuhui4403@sina.com
Aijun Pan	aijunpan@fsyy.ustc.edu.cn
Xiaobo Huang	814129752@qq.com
Chuming Fan	fanchuming@sina.com
Weihua Lu	lwh683@126.com
Dongwu Shi	shidw1@163.com
Lei Wang	413060682@163.com
Wei Li	sicu.lw@163.com
Liming Gu	glm39887@126.com
Yingguang Xie	zzyxkx@163.com
Rongqing Sun	rongqing.sun@126.com
Feng Guo	zjguofeng@aliyun.com

Lin Han	xhan0507@sina.com
Lihua Zhou	xiulika@163.com
Xiangde Zheng	zxdfg123@163.com
Feng Shan	mountain1963@163.com
Jianbo Liu	qicaixia2007@126.com
Yuhang Ai	ayhicu1978@sina.com
Yan Qu	qdquyan@aliyun.com
Liandi Li	liliandi75@sina.com
Hailing Li	lihailing608@163.com
Zhiguo Pan	gzpzg@126.com
Donglin Xu	syicuxdl@126.com
Zhiqiang Zou	396746023@qq.com
Yan Gao	Gaoyan1515@163.com
Chunli Yang	646233247@qq.com
Qiuye Kou	kouqiuye@126.com
Xijing Zhang	zhangxj918@163.com
Jinglan Wu	doctorwu99@126.com
Chuanyun Qian	qianchuanyun@126.com
Weixing Zhang	zhangwx@hotmail.com
Minjie Zhang	hychen427@163.com
Yuan Zong	chuchu20040317@qq.com
Bingyu Qin	nicolasby@126.com
Fusen Zhang	13793812109@126.com
Zhe Zhai	zhaizhe0357@163.com
Yun Sun	sunyun15@163.com
Ping Chang	changp963@163.com
Bo Yu	yuboicu@csu.edu.cn
Min Yu	ycyyicuyumin@163.com
Shiying Yuan	yuan_shiying@163.com
Yijun Deng	dengyj2003@163.com
Liyun Zhao	403756433@qq.com
Bin Zang	zangb@sj-hospital.org
Yuanfei Li	156636305@qq.com

Fachun Zhou	zfc88@126.com
Xiaomei Chen	chenxm008@163.com
Min Shao	shaominmail @yahoo.com
Weidong Wu	13503545010@163.com
Ming Wu	Boshiyy@126.com
Zhaohui Zhang	1410956890@qq.com
Yimin Li	dryiminli@vip.163.com
Qiang Guo	ggqq7910@163.com
Zhiyong Wang	18533112993@126.com
Yuanqi Gong	760225787@qq.com
Yunlin Song	17929294@qq.com
Kejian Qian	2682244275@qq.com
Yongjian Feng	horse1980@163.com
Baocai Fu	kailiaokailiao@sina.com
Xueyan Liu	1736574753@qq.com
Zhiping Li	354855270@qq.com
Chuanyong Gong	chuanyonggong@163.com
Cheng Sun	suncheng2008@139.com
Jian Yu	yujian0427@163.com
Zhongzhi Tang	Jzktzz_wh2014@163.com
Linxi Huang	13502733122@126.com
Biao Ma	305058382@qq.com
Zhijie He	805545775@qq.com
Qingshan Zhou	white1962@qq.com
Rongguo Yu	garyyrg@yahoo.com

2.3 Coordinating and data management center

Coordinating and data management centers (CDMC) was organized before the implementation of the current study. They are responsible for day to day management of the trial, assistance for ethic application in each center, protocol and case report form design, online database design and maintenance, protocol and case report form design, online database design and maintenance, protocol training for the participating

centers, enrollment, data entry and quality control, severe adverse event monitor and notification and data analysis. The CDMC plans to meet before enrollment, three months after initial enrollment, and six months after initial enrollment to ensure qualified data entry.

Members of CDMC are listed below

Prof. Zhihui Tong

Prof. Lu Ke

Dr. Jing Zhou

Mr. Yafei Yan

Dr. Juan Xing

Dr. Jiajia Lin

Ms. Yan Chen

2.4 Writing and publication committee

The writing and publication committee is responsible for drafting the manuscript and submission of the manuscript to adequate journals. The Writing and publication committee will also decide on the authorship of this study. After the conclusion of this study, all participating centers are welcome to submit proposals for post-hoc analysis to the writing and publication committee is responsible for reviewing and rating all the proposals for further analysis.

Members are listed below

Prof. Weiqin Li

Prof. Lu Ke

Dr. Yang Wang

Prof. Zhongheng Zhang

Prof. Gordon Doig

Prof. Arthur R.H. van Zanten

Ms. Jiajia Lin

2.5 Data and safety monitoring board

Data and safety monitoring board (DSMB) is an independent group of experts that offers advice during the implementation of the study. The DSMB can recommend that a trial be stopped early because of concerns about participant safety.

Members of DSMB are listed below

Prof. Yuxiu Liu

Dr. Mengjie Lu

Prof. Wenkui Yu

Prof. Qiang Li

2.6 Registration

The NEED trial was registered on the ISRCTN registry (ISRCTN12233792) before commencement of the study.

2.7 Funding

The study was funded partly by the Key Research and Development Program Foundation of Jiangsu Province of China (No. BE2015685) and partly by the Nutricia, Wuxi, China.

3. Background and rationale

3.1 The role of nutritional therapy in critical care

In the intensive care unit (ICU), nutritional therapy is one of the most crucial treatments for critically ill patients, which may significantly influence the clinical outcomes [1,2]. There is a large body of evidence showing that malnutrition is associated with a significantly increased risk of death. Besides, ICU patients are prone

to suffer from underfeeding, which could further exacerbate the existing gap between energy demand and intake [3]. The route of nutrition delivery is another important issue when starting nutritional therapy for ICU patients. Enteral feeding has been repeatedly proven to be superior over parenteral nutrition (PN) with respect to clinical outcomes such as nosocomial infection, mortality, and also medical resource utilization [4-7].

As nutritional therapy involves a battery of interventions and procedures, a variety of clinical practice guidelines have been published to standardize nutritional therapy. However, it was reported that the adherence to these guidelines was suboptimal so that the clinical practices of feeding vary substantially across different regions and hospitals. Moreover, it remains controversial whether a feeding guideline could improve the adherence to current international guidelines and therefore benefit the patients in terms of clinical outcomes [8-11].

3.2 The rationale for conducting this study

China has the world’s largest population of critically ill patients, and nutritional therapy varies massively. Previous small studies have shown that the proportion of EN was as low as 40% on the second day after ICU admission, which could be potentially improved with the implementation of a feeding guideline [12]. Our previous cross-sectional study showed that the proportions of study subjects starting EN within 24, 48 and 72 h after ICU entry were 24.8% (84/352), 32.7% (150/459) and 40.0% (200/541), respectively, suggesting the unsatisfying adherence to the current international guidelines and huge space for improvement [13]. Accordingly, we developed an evidence-based, multifaceted, and practical feeding guideline and related educational materials to overcome barriers and enhance nutritional therapy performance. A before-and-after pilot study was conducted in 2016 to test the feasibility of the feeding guideline [12]. The results showed that it significantly changed the practice but failed to provide clinically meaningful benefits, warranting a large-scale study to verify the findings further. Considering the possibility that

patients assigned to the control group may receive nutritional therapy guided or partly guided by the feeding guideline if it was simultaneously operational at the same institute, randomization was performed at ICU-level rather than individual patient level. A cluster-randomized method can effectively control the confounding factors between groups to obtain more reliable conclusions.

4. Study Design

4.1 Aims and hypotheses

We aimed to assess the effect of an evidence-based feeding guideline on nutritional therapy and clinical outcomes in critically ill patients. The hypothesis is that a feeding guideline, compared to routine clinical practice, could reduce 28-day mortality in critically ill patients. The null hypothesis is there is no difference in terms of 28-day mortality in patients assigned to either feeding guideline group or routine group.

4.2 General study setting

The present clinical interventional study will be performed in 97 different ICUs across China with different clinical settings, including surgical ICU, medical ICU, neurological ICU, etc. It is a stratified, cluster-randomized, parallel-controlled study. Stratified randomization will be used based on the provincial/state distribution of the hospitals and type of ICUs (emergency, medical, surgical, neurosurgery, and general). Study treatment assignment will not be blinded, but the allocation of study groups will be concealed before randomization.

4.3 Sample size

According to previous studies in China, we presumed that the 28-day mortality for mixed ICU patients was 20%, which was used as the event rate in the control group.

The nutrition guideline was assumed to be able to reduce the mortality rate by 8% at least. The type I error was 0.05, and the statistical power was 80%. The inter-class correlation was 0.1. A total of 90 centers with 2250 participants were required to meet the statistical power. The sample size calculation was performed by the CRTSize package. The full code for the calculation was: n4props (pe=0.12, pc=0.20, m=25, ICC=0.10, AR=1, alpha=0.05, power=0.80).

5. Study population

5.1 Patient recruitment

We are going to recruit approximately 2250 patients presenting to the 97 participating ICUs into the study during an estimated one-year period. Based on the volume of all the participating centers, the aim should be able to be achieved within the study period.

5.2 Eligibility Criteria

5.21 Inclusion Criteria

1. Informed consent form obtained from the patient or next of kin;
2. 18 years old or older;
3. Within 24h of ICU admission;
4. With one or more organ failure (SOFA for any individual organ system≥2);
5. Expected to stay in ICU for more than seven days;
6. Oral diet is not likely to be restored within three days.

5.22 Exclusion Criteria

1. Patients receive EN in the past three days before admission;
2. Patients receiving palliative treatment or expected to die within 48 hours;
3. Women in pregnancy;
4. Long-term use of steroids or immunosuppressive agents;

5. Patients with malignant diseases receiving radiotherapy or chemotherapy.

6.Randomization and assignment of interventions

6.1 Patients screening procedures

All patients presenting to the ICUs of each participating hospital will be assessed by the treating physician and receive medical care immediately according to the current best clinical practice. The treating clinician team will be responsible for identifying potential patients and contact the NEED coordinator or a member of the NEED study team who will assess the patients for eligibility into the study. Screening tools will be provided, and a screening log will be kept.

Each participating center will be led by a NEED project leader (center primary investigator) and a site coordinator. The former will be responsible for implementing the feeding guideline if assigned to the study group and monitor the NEED trial. The latter will be responsible for daily screening and data management.

6.2 Recruitment

All patients admitted to the participating ICUs will be considered for enrollment and assessed for inclusion and exclusion criteria. Where possible, informed consent will be obtained from the patient or next of kin (NOK) prior to enrollment into the study.

6.3 Randomization procedures

6.3.1 Sequence generation

The R language will be used for randomization for this study. All the participating centers will be stratified according to the provincial/state distribution of the hospitals and types of ICUs (emergency, medical, surgical, neurosurgery, and general). Randomization will occur in a 1:1 fashion for the participating sites within the same

category with computer-generated random numbers. Allocation concealment was maintained by conducting randomization after consent to participate was obtained from the sites.

6.3.2 Blinding method

This study is an open-label study, and no blinding method would be applied for both the participants and the investigators.

6.4. Study procedures

6.4.1 Interventional arms

Arm#1 feeding guideline group

Before EN initiation, hemodynamic parameters should be stabilized evidenced by MAP≥65 mmHg and lactate<4mmol/L, with decreasing vasoactive dose. The gastrointestinal function will then be evaluated with the acute gastrointestinal injury (AGI) grading system. For patients with AGI of I or none, EN will be started at 25 ml/h. For patients with AGI II-III, predigested EN will be started at 10–15 ml/h. EN should be withheld for those with AGI IV. If patients are at high risk of malnutrition based on the Nutric score ≥5 (IL-6 not included), but EN cannot be initiated, PN should be started. Otherwise, PN will be withheld for 7–10 days. Patients on EN will be evaluated using a tolerance score for every 4-6 hours. The graphic feeding protocol derived from the guideline and the tolerance score are shown in Figure 1 and Table 1, respectively. EN will be discontinued when the EN tolerance score is greater than 5 points. Adverse events will be treated and managed with a standardized protocol (Figure 2) by the treating team. In detail, EN will be discontinued in the presence of persistent abdominal pain. Physical examination and abdominal computed tomography will be ordered if deemed necessary by the treating team. If there are signs of bowel obstruction and/or ischemia, EN should be discontinued immediately. Diarrhea can be caused by enteral feeding, specific diseases and drugs, and infections, which should be considered and diagnosed by the treating team. If *Clostridium difficile* infection is identified, the patient will be treated with metronidazole or vancomycin.

Arm#2 Control group

All the participants in this group will be treated without any change to the current clinical practice. No adherence to uniform protocol or guidelines will be required during the study period.

6.4.2 General management

All patients will be cared for by the local treating team at each participating ICU. In the control group, nutritional therapy would be implemented routinely at the discretion of the treating team. All co-interventions will be left to the discretion of the treating clinical teams and recorded in the patient's medical record.

7. Outcome measures

7.1 Primary outcome measures

The primary outcome measure is all-cause mortality at day 28 after enrollment (the day of enrollment will be set as day 1).

7.2 Secondary outcome measures

7.2.1 Process measures

1. Time to start EN
2. Time to start PN
3. Mean nutrition support days within first seven days after enrollment
    3.1 Mean nutritional support(either EN or PN or both) days within first seven days after enrollment
    3.2 Mean EN support days within first seven days after enrollment
    3.3 Mean PN support days within first s even days after enrollment
4. Mean energy per day over the first seven days for patients who were fed
    4.1 Mean EN per day over the first seven days for patients who were fed
    4.2 Mean PN per day over the first seven days for patients who were fed
5. The proportion of patients never fed during the first seven days after enrollment
6. The proportion of patients received EN within two days after enrollment

7. The proportion of patients received PN within two days after enrollment
8. The proportion of fed patients within two days after enrollment
9. The proportion of patients received EN or PN within two days after enrollment
10. EN tolerance score during the first seven days after enrollment
11. Days requiring prokinetic agents within the first seven days after enrollment
12. The proportion of patients who received a post-pyloric feeding tube (patients receiving EN) within first seven days after enrollment.

7.2.2 Organ dysfunction-related outcomes

1.New-onset organ failure within first seven days
   1.1 New-onset respiratory failure;
   1.2 New-onset cardiovascular failure;
   1.3 New-onset renal failure
2. New receipt of organ support therapy within first seven days
   2.1 New receipt of mechanical ventilation (non-invasive included)
   2.2 New receipt of renal replacement therapy
   2.3 New receipt of vasoactive agents
   2.4 Days requiring CRRT within first seven days after enrollment
   2.5 Days requiring insulin within first seven days after enrollment
   2.6 Days requiring MV within first seven days after enrollment

7.2.3 Key secondary outcomes

1. Incidence of new infection in ICU
2. ICU-free days within 28 days

7.3 Definition of outcomes

New-onset organ failure: organ failure occurring during the first seven days and not present at enrollment. Organ failure is defined as an increase in the Sequential Organ Failure Assessment (SOFA) score of 2 points or more for each organ system(Respiration, Cardiovascular or Renal).

New receipt of organ support therapy: requirement of organ support therapy (mechanical ventilation, renal replacement therapy, and vasoactive agents) not applied at enrollment.

All-cause mortality at day 28: defined as patient vital status (alive/dead) to be determined on the 28th calendar day post-enrollment (the day of enrollment will be set as day1).

New infection in the ICU: defined as a new infection appearing at least 48 hrs after ICU admission. The diagnosis of infection was made on the basis of medical history, physical examination, and the results of laboratory examinations requiring antimicrobial therapy.

ICU-free days within 28 days: defined as days alive and free from the need for intensive care) from enrollment to day 28. In patients who admitted to the study ICU more than once through day 28, only the final period of ICU stay was included in ICU-free days. Patients who ICU discharged on day 28 or died before day 28 were assigned zero ICU-free days.

8. Data management, analysis, and statistics

8.1 Data collection

All the data that is necessary to define baseline patient characteristics, the implementation of the feeding guideline and control therapies, potential confounding co-interventions, and outcomes will be collected.

The primary investigator of each site will be responsible for patients enrollment and data input. A group of statisticians will be accountable for the pre-definition of statistical analysis and subgroup analysis.

A web-based electrical database will be used for data collection and storage. All data will be input by the primary investigator or nominated investigator (less than two for each participating center) approved by the primary investigator. Training for data entry will be performed by the supplier of the electrical database and the sponsor of the NEED trial.

8.2 Participant timeline

All patients enrolled will be followed until either 28 days after enrollment or death, depending on which comes first. Follow-up will be restricted to information regarding the vital status and other related study clinical outcomes. Follow-up will be conducted by the study staff through either direct contact with the patient or their next of kin. Patients who withdraw from the study for any reason will also be followed up according to the study follow-up schedule and analyzed on an intention-to-treat principle unless they withdraw from data collection and use.

8.3 The flow of ICUs and patients

The flow of hospitals and patients through the trial will be reported in accordance with the CONSORT extension statement for cluster trials. The flow diagram will include the number of eligible and recruited sites, the number of eligible and recruited patients and then, by allocated group, the number of patients who continued through the trial, the number of withdrawing, the number of data missing, and the numbers included in the analysis.

8.4 Basic principle of analysis

The reporting and presentation of this trial will be following the CONSORT guidelines for cluster-randomized trials [14], with the primary comparative analysis being conducted on an intention-to-treat basis.

Descriptive statistics will be used to assess any marked baseline differences in demographics or outcome measures between the two groups, taking clustering into account. Comparisons of binary outcomes will be expressed as relative difference with 95% confidence intervals and comparisons of continuous outcomes as mean differences together with 95% confidence intervals. Between-group comparisons will be made using a generalized linear mixed-model (weighed by clusters).

All analyses will account for clustering to ensure correct type I error rates and confidence intervals. Our cluster-randomized trial will be analyzed at the individual level, and account for clustering among patients in the same cluster.

Based on the principle of intention to treat (ITT), full-analysis set (FAS) will be performed on all the randomized subjects. FAS will be used for the analysis of baseline characteristics and main therapeutic interventions

Two-sided 5% significance levels will be used to identify statistically significant results. All confidence intervals reported will be 95% confidence intervals. All p-values and estimates of change will be calculated at the individual level.

8.5 Interim analysis

A formal interim analysis will be performed at study mid-recruitment to ensure the safety of the participants. The DSMB will meet and determine whether the trial should continue.

9.Ethics and dissemination

This study has been approved by the ethics committee of the Jinling Hospital.

Ethics approval of each participating center is required before initiation of enrollment.

9.1 Ethical issues of this study

The major ethical considerations include:

1. Some potential participants are unable to give consent for themselves;
2. The nutritional therapy in the control arm will be conventional.

This study has been approved by the ethics committee of the Jinling Hospital, Nanjing University, which is the sponsor of the NEED trial. Ethics approval of each participating center is required before initiation of enrollment.

9.2 Potential risks and benefits

As the feeding guideline used in this study has been tested in a pilot study without significant adverse effects, the application of this protocol should be largely safe. No specific monetary compensation is available for each participant in this study.

9.3 Consent and confidentiality

Informed consent is required for each participant of this study, either signed by the patients himself or next of kin. All the data stored in the electronic database are de-identified to guarantee the patients' privacy.

9.4 Dissemination policy

All the primary investigators of the participating ICUs and the sponsor will have full access to the data after the conclusion of the study. Anyone who wants to do a post-hoc analysis needs to submit a formal writing proposal to the *writing and publication committee*. Only approved authors can have access to the database.

10. Safety issues and monitoring

10.1 Data and safety monitoring board

Data and safety monitoring board (DSMB) is an independent group of experts that offers advice during the implementation of the study. The DSMB can recommend that a trial be stopped early because of concerns about participant safety.

10.2 Adverse events

Adverse events (AEs) are defined in accordance with the National Cancer Institute-Common Terminology Criteria for Adverse Events (National Cancer Institute-

Common Terminology Criteria for Adverse Events) as any untoward medical occurrence in a patient or clinical investigation subject administered an investigational intervention and which does not necessarily have to have a causal relationship with this treatment.

It is recognized that the patient population admitted to ICUs will experience a number of common aberrations in laboratory values, signs, and symptoms due to the severity of the underlying disease and the impact of standard therapies. These will not necessarily constitute an adverse event unless they require significant intervention or are considered to be of concern in the investigator's clinical judgment.

In all cases, the condition or disease underlying the symptom, sign, or laboratory value should be reported, e.g., renal failure rather than hyperkalemia and agitation rather than self-extubation.

10.3 Serious adverse events

SAEs are defined when any untoward medical occurrence that:

1.Results in death

2.Is life-threatening

3.Requires inpatient hospitalization or prolongation of existing hospitalization

4. Results in persistent or significant disability/incapacity

5. Is a congenital anomaly/birth defect

6. Is an important medical event that may require intervention to prevent one of the previously listed outcomes.

In this study, all SAEs will be reported regardless of suspected causality.

10.4 Monitoring of potential adverse events

The DSMB will be responsible for overseeing all subjects' safety and monitoring total mortality and serious adverse events. All serious adverse events occurring during the trial will be reported to the Coordinating and data management center (CDMC) within 48 hours. Minimum information to report will include:

1. Initials of the patient and study number

2. Course and nature of the event

3. An investigator's opinion of the relationship between study involvement and the event (unrelated, possibly, probably or definitely related)

4. Whether treatment is required and what treatment was applied

The contact information for CDMC

Telephone number

+86 80860007

+86 80863073

Mobile number of the primary investigator:
+86 13951839654

11. References

1. Stuani Franzosi O, Delfino von Frankenberg A, Loss SH, Silva Leite Nunes D, Rios Vieira SR. Underfeeding versus full enteral feeding in critically ill patients with acute respiratory failure: a systematic review with meta-analysis of randomized controlled trials. Nutr Hosp. 2017; 34: 19–29.
2. Hofman Z, Swinkels S, van Zanten ARH. Glutamine, fish oil and antioxidants in critical illness: MetaPlus trial post hoc safety analysis. Ann Intensive Care. Springer Paris; 2016; 6: 119.
3. Heyland DK, Dhaliwal R, Wang M, Day AG. The prevalence of iatrogenic underfeeding in the nutritionally "at-risk" critically ill patient: Results of an international, multicenter, prospective study. Clin Nutr. Elsevier; 2015; 34: 659–666.
4. McClave SA, Taylor BE, Martindale RG, Warren MM, Johnson DR, Braunschweig C, et al. Guidelines for the Provision and Assessment of Nutrition Support Therapy in the Adult Critically Ill Patient: Society of Critical Care Medicine (SCCM) and American Society for Parenteral and Enteral Nutrition

(ASPEN). Vol. 40, JPEN. Journal of parenteral and enteral nutrition. 2016. pp. 159–211.

5. Gramlich L, Kichian K, Pinilla J, Rodych NJ, Dhaliwal R, Heyland DK. Does enteral nutrition compared to parenteral nutrition result in better outcomes in critically ill adult patients? A systematic review of the literature. Nutrition. Elsevier; 2004 Oct;20(10):843–8.
6. Elke G, van Zanten ARH, Lemieux M, McCall M, Jeejeebhoy KN, Kott M, et al. Enteral versus parenteral nutrition in critically ill patients: an updated systematic review and meta-analysis of randomized controlled trials. Crit Care. BioMed Central; 2016 Apr 29;20(1):117.
7. Heyland DK, Dhaliwal R, Drover JW, Gramlich L, Dodek P, Canadian Critical Care Clinical Practice Guidelines Committee. Canadian clinical practice guidelines for nutrition support in mechanically ventilated, critically ill adult patients. Vol. 27, JPEN. Journal of parenteral and enteral nutrition. SAGE PublicationsSage UK: London, England; 2003. pp. 355–73.
8. Padar M, Uusvel G, Starkopf L, Starkopf J, Reintam Blaser A. Implementation of enteral feeding protocol in an intensive care unit: Before-and-after study. World J Crit Care Med. 2017; 6: 56–64.
9. Cunningham CA, Gervais LB, Mazurak VC, Anand V, Garros D, Crick K, et al. adherence to a NurseDriven Feeding Protocol in a Pediatric Intensive Care Unit. JPEN J Parenter Enteral Nutr. SAGE PublicationsSage CA: Los Angeles, CA; 2017; 170: 148607117692751.
10. Ventura AMC, Waitzberg DL. Enteral nutrition protocols for critically ill patients: are they necessary? Nutr Clin Pract. SAGE PublicationsSage CA: Los Angeles, CA; 2015; 30: 351–362.
11. Mehta NM, Bechard LJ, Cahill N, Wang M, Day A, Duggan CP, et al. Nutritional practices and their relationship to clinical outcomes in critically ill children—an international multicenter cohort study*. Crit Care Med. 2012; 40: 2204–2211..
12. Li Q, Zhang Z, Xie B, Ji X, Lu J, Jiang R, et al. Effectiveness of enteral feeding protocol on clinical outcomes in critically ill patients: A before and after study.

Brakenridge S, editor. PLoS ONE. Public Library of Science; 2017;12(8):e0182393.

13. Xing J, Zhang Z, Ke L, Zhou J, Qin B, Liang H, Chen X, Liu W, Liu Z, Ai Y et al: Enteral nutrition feeding in Chinese intensive care units: a cross-sectional study involving 116 hospitals. Crit Care 2018, 22(1):229.

14. Hemming, K.; Taljaard, M.; McKenzie, J. E.; Hooper, R.; Copas, A.; Thompson, J. A.; Dixon-Woods, M.; Aldcroft, A.; Doussau, A.; Grayling, M.; Kristunas, C.; Goldstein, C. E.; Campbell, M. K.; Girling, A.; Eldridge, S.; Campbell, M. J.; Lilford, R. J.; Weijer, C.; Forbes, A. B.; Grimshaw, J. M., Reporting of stepped wedge cluster randomised trials: extension of the CONSORT 2010 statement with explanation and elaboration. BMJ 2018, 363, k1614.

Figure 1: Graphic Feeding Protocol

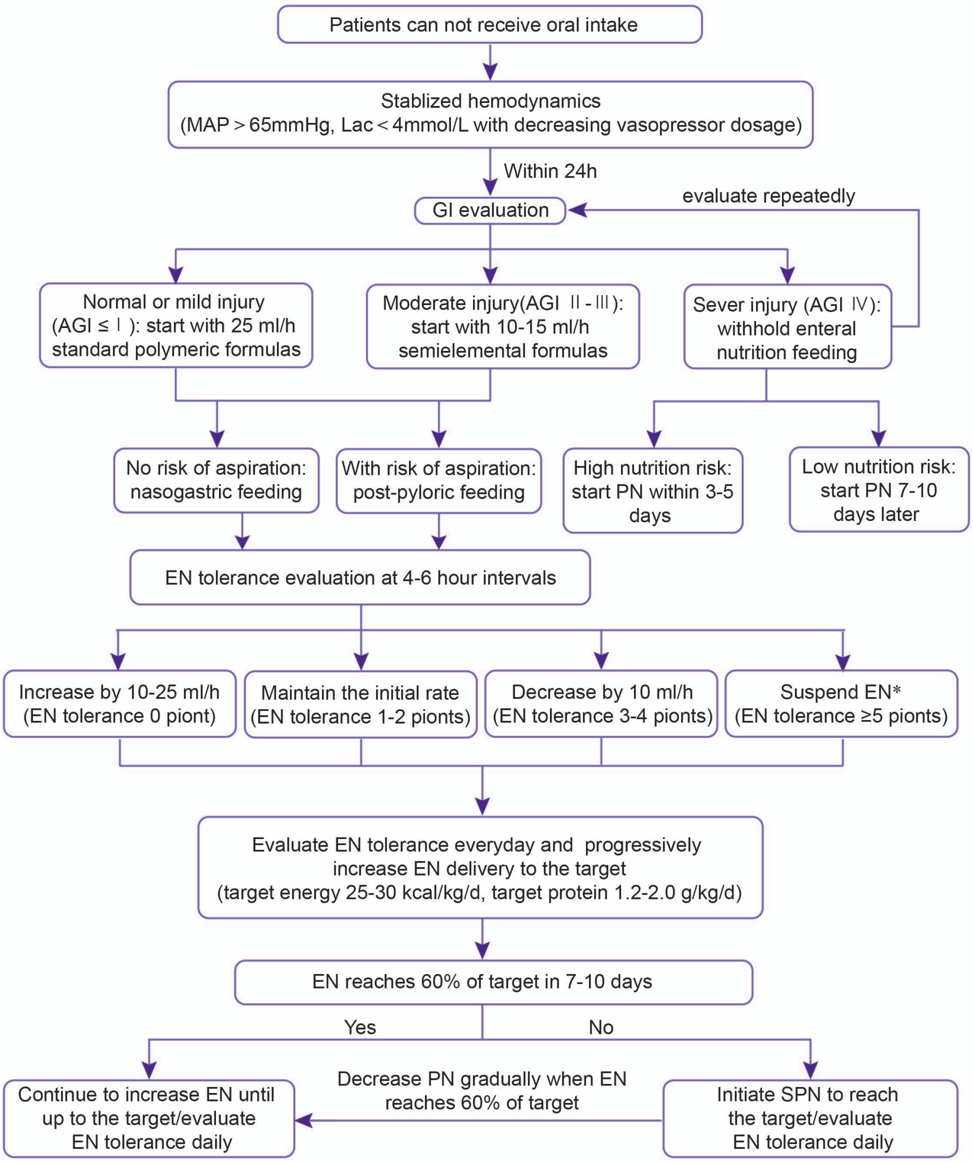

Figure 2: Protocols for the management of adverse events

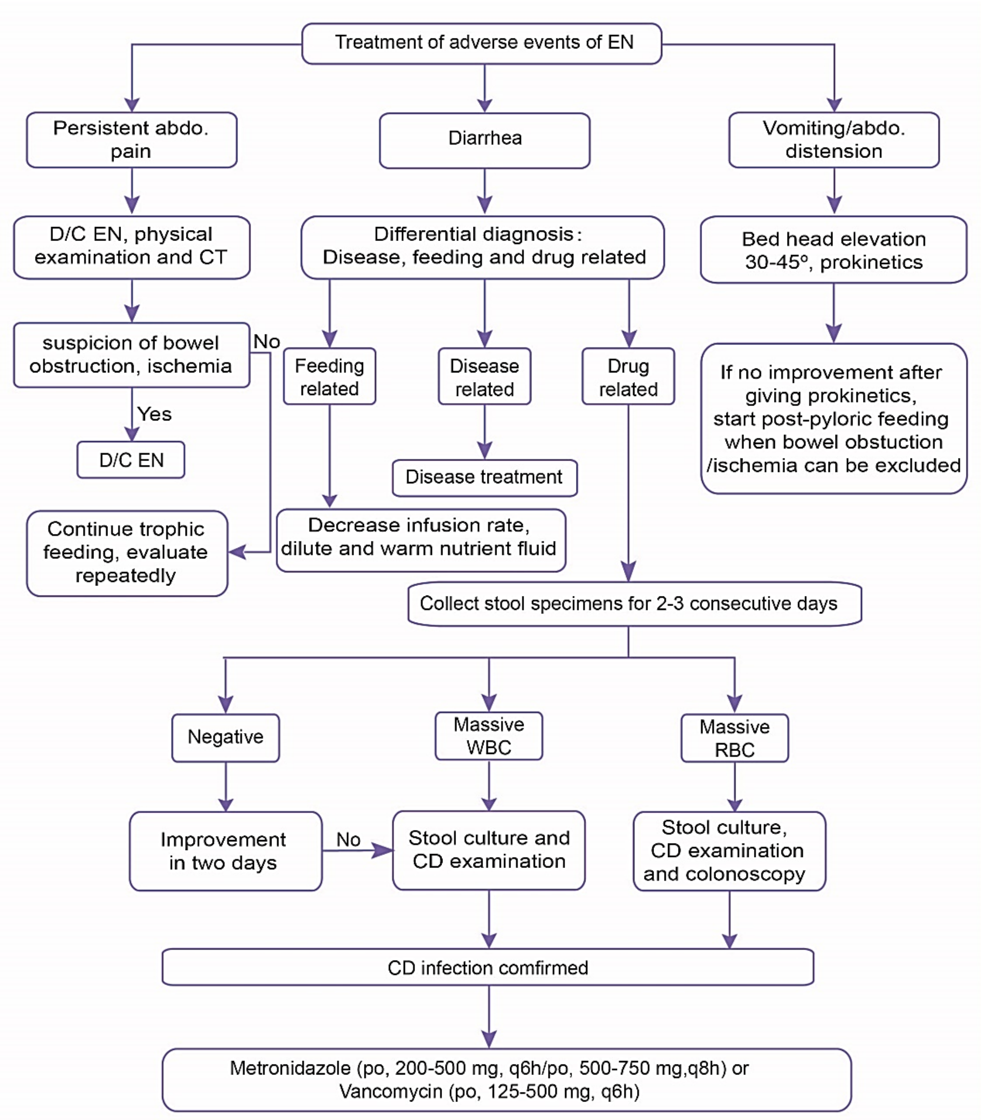

Table 1: EN tolerance score

Points	0	1	2	5
Abdominal distension/pain	None	Mild distension and no distension	Moderate distension **OR** IAP 15~20mmHg **OR** Spontaneous resolution of abdominal pain	Severe distension **OR** IAP>20mmHg **OR** No spontaneous resolution of abdominal pain
Nausea/vomiting	None **OR** Continuous gastric decompression without symptom	Nausea but no vomiting	Nausea and vomiting without need for decompression **OR** 250ml≦ GRV<500ml	Vomiting requiring gastric decompression **OR** GRV≧500ml
Diarrhea	None	Loose stools≧3 times/day with 250≦ volume<500 ml	Loose stools≧3 times/day with 500≦ volume<1500ml	Loose stools≧3 times/day with volume≧1500ml

Total score= Abdominal distension/pain + Nausea/vomiting + Diarrhea

0-2 points: continue enteral nutrition, increase or maintain initial speed, symptomatic treatment;

3-4 points: continue enteral nutrition, slow down the speed, reevaluate EN tolerance after 2h;

≥ 5 points: suspend enteral nutrition, reevaluate or replace the infusion route;

Appendix 1. Participant ICUs

Participant center	ICU type
Anhui Provincial Hospital	General ICU
Yijishan Hospital of Wannan Medical College	General ICU
Anhui Medical University Second Affiliated Hospital	General ICU
The First Affiliated Hospital of Anhui Medical University	General ICU
First Affiliated Hospital of Fujian Medical University	General ICU
The People’s Hospital of Fujian Province	General ICU
Union Hospital of Fujian Medical University	General ICU
Fujian Provincial Hospital	Surgical ICU
First People's Hospital of Foshan	General ICU
General Hospital of Southern Theatre Command	Emergency ICU
Guangzhou First People's Hospital	General ICU
The Sixth Affiliated Hospital, Sun Yat-Sen University	General ICU
Huazhong University of Science and Technology Union Shenzhen Hospital	General ICU
Peking University Shenzhen Hospital	General ICU
General ICU, Jinan University First Affiliated Hospital	Surgical ICU
Southern Medical University Zhujiang Hospital	General ICU
Guangdong Second Traditional Chinese Medicine Hospital	General ICU
The Second People’s Hospital of Shenzhen	Medical ICU
First Affiliated Hospital of Guangzhou Medical University	General ICU
Jinan University First Affiliated Hospital	Neurosurgical ICU
Shenzhen People's Hospital	General ICU
Guangdong Provincial People's Hospital	General ICU
Shantou University Medical College First Affiliated Hospital	General ICU
Sun Yat-sen Memorial Hospital, Sun Yat-sen University	General ICU
The First Affiliated Hospital, Sun Yat-sen University	Surgical ICU
Zhongshan People's Hospital	General ICU
the First Affiliated Hospital of Guangzhou University of Chinese Medicine	General ICU
People's Hospital of Guangxi Zhuang Autonomous Region	Medical ICU
North China University of Science and Technology Affiliated Hospital	General ICU
Tangshan Gongren Hospital	General ICU
Hebei Medical University Second Affiliated Hospital	General ICU
Hebei Medical University Third Affiliated Hospital	General ICU
Luoyang Central Hospital Affiliated to Zhengzhou University	General ICU
Zhengzhou University First Affiliated Hospital	General ICU
Henan Provincial People's Hospital	General ICU
The Second Affiliated Hospital of Harbin Medical University	General ICU
The Fourth Hospital of Medical University	General ICU
First People's Hospital of Yichang	General ICU
Union Hospital Affiliated to Tongji Medical College of Huazhong University of Science and Technology	General ICU
Yichang Central People's Hospital	General ICU
Wuhan General Hospital of Guangzhou Military Region	General ICU

Hubei Provincial People's Hospital	General ICU
Zhongnan Hospital of Wuhan University	General ICU
The Third Xiangya Hospital of Central South University	General ICU
Xiangya Hospital Central South University	General ICU
The Second Xiangya Hospital of Central South University	General ICU
Changsha Central Hospital	General ICU
Hunan Provincial People's Hospital	General ICU
Suzhou Municipal Hospital	General ICU
Changzhou No.2 People's Hospital affiliated to Nanjing Medical University	General ICU
Wuxi People's Hospital	General ICU
Benq Medical Center	General ICU
Jinling Hospital	Surgical ICU
Yancheng First People's Hospital	General ICU
First Affiliated Hospital of Soochow University	Emergency ICU
Jiangxi Provincial People's Hospital	General ICU
Nanchang University Second Affiliated Hospital	General ICU
The First Affiliated Hospital of Nanchang University	General ICU
The General Hospital of Shenyang Military	Emergency ICU
China Medical University Second Affiliated Hospital	General ICU
The Second Hospital of Dalian Medical University	General ICU
Affiliated Hospital of Inner Mongolia Medical College	General ICU
Inner Mongolia People's Hospital	General ICU
Qindao University Medical College Affiliated Yantai Yuhuangding Hospital	General ICU
Zibo Central Hospital	General ICU
Linyi City People Hospital	General ICU
Jining First People's Hospital	General ICU
Qindao University Medical College Affiliated Hospital	General ICU
Qingdao Municipal Hospital Group	General ICU
Qindao University Medical College Affiliated Hospital	General ICU
No.971 hospital of People’s Liberation Army Navy	General ICU
Tai'an City Central Hospital	General ICU
Qilu Hospital, Shandong University	General ICU
Yantai Mountain Hospital	General ICU
Jining Medical College Affiliated Hospital	General ICU
Qindao University Medical College Affiliated Yantai Yuhuangding Hospital	Neurosurgical ICU
Shanxi Provincial People's Hospital	General ICU
Shanxi Medical University First Affiliated Hospital	General ICU
Shanxi Bethune Hospital	General ICU
First Affiliated Hospital of Xi'an Jiao Tong University	General ICU
Xijing Hospital	General ICU
Shaanxi Provincial People's Hospital	General ICU
Chengdu University of Traditional Chinese Medicine Affiliated Hospital	General ICU
Sichuan Provincial People's Hospital	General ICU

Dazhou Central Hospital	General ICU
Tianjing Hospital of Integration of Chinese and Western Medicine	General ICU
Tianjin Union Medical Center	General ICU
The First Affiliated Hospital of Xinjiang Medical University	General ICU
General Hospital of Xinjiang Military Command	General ICU
First People's Hospital of Kunming	General ICU
First People's Hospital of Yunnan	General ICU
People's Hospital of Yuxi City	General ICU
Kuming Medical University First Affiliated Hospital	Emergency ICU
Zhejiang University School of Medicine Sir Run Run Shaw Hospital	General ICU
Daping Hospital, Army Medical University	General ICU
The Second Affiliated Hospital of Chongqing Medical University	General ICU
Chongqing Medical University First Affiliated Hospital	General ICU

Summary of protocol changes

List of Changes

Protocol Amendment 1 (version 2, 07 Feb 2018)

Page/ Line No.	Original text	New text	Reason
Page 10, Section3.1 The role of nutritional therapy in critical care Line 15	Moreover, it remains controversial whether feeding protocol is effective in improving clinical outcomes such as mortality, nosocomial infections, duration of mechanical ventilation, and length of stay in ICU	Moreover, it remains controversial whether a feeding guideline could improve the adherence to current international guidelines and therefore benefit the patients in terms of clinical outcomes	Describe more accurate
Page10, Section 3.2 The rationale for conducting this study Line 1	China has the world's largest population of critically ill patients, and the clinical practice of nutrition therapy varies massively.	China has the world's largest population of critically ill patients, and nutritional therapy varies massively.	Describe more accurate
Page10, Section 3.2 The rationale for conducting this study Line 2	Previous small studies have shown that the proportion of EN was as low as 40% on the second day after ICU admission, which could be potentially improved with the implementation of a feeding protocol.	Previous small studies have shown that the proportion of EN was as low as 40% on the second day after ICU admission, which could be potentially improved with the implementation of a feeding guideline.	Change "a feeding protocol" to "a feeding guideline."
Page 10, Section 3.2 The rationale for conducting this study Line 8	A cluster randomized method can effectively control the confounding factors between groups to obtain more reliable conclusions. In addition, the interventional study of feeding protocol would lead to pollution effect. Randomization on the hospital level and stratification could avoid the pollution.	Accordingly, we developed an evidence-based, multifaceted, and practical feeding guideline and related educational materials to overcome barriers and enhance nutritional therapy performance. A before-and-after pilot study was conducted in 2016 to test the feasibility of the feeding guideline. The results showed that it significantly changed the practice but failed to provide clinically meaningful benefits, warranting a large-scale study to verify the findings further. Considering the possibility that patients assigned to the control group may receive nutritional therapy guided or partly guided by the feeding guideline if it was simultaneously operational at the same institute, randomization was performed at ICU-level	Describe in detail

		rather than individual patient level. A cluster-randomized method can effectively control the confounding factors between groups to obtain more reliable conclusions.	
Page 11, Section 4.1 Aim of the study First Line	4.1 Aim of the study<br>We aimed to verify the effect of feeding protocol on nutrition therapy and clinical outcome in critically ill patients.	4.1 Aims and hypotheses<br>We aimed to assess the effect of an evidence-based feeding guideline on nutritional therapy and clinical outcomes in critically ill patients. The hypothesis is that a feeding guideline, compared to routine clinical practice, could reduce 28-day mortality in critically ill patients. The null hypothesis is there is no difference in terms of 28-day mortality in patients assigned to either feeding guideline group or routine group.	Describe more accurate
Page 12, Section 5.2 Eligibility criteria First Line	5.2 Eligibility Criteria<br>5.21 Inclusion Criteria<br>1. 18 years old or older;<br>2. Expected to stay in ICU for more than three days;<br>5.22 Exclusion Criteria<br>1. Subjects receiving EN in previous seven days;<br>2. Contraindications for nasogastric or nasoenteric tube placement;<br>3. Subjects who have already undergone percutaneous endoscopic jejunostomy (PEJ), percutaneous endoscopic gastrostomy (PEG) and surgical jejunostomy;<br>4. Age younger than 18 years old;<br>5. Women who are pregnant or undergo breast feeding;<br>6. Burn patients;	5.2 Eligibility Criteria (**This change was made before commencement of patient enrollment in Protocol V2.0**)<br>5.21 Inclusion Criteria<br>1. Informed consent form obtained from the patient or next of kin;<br>2. 18 years old or older;<br>3. Within 24h of ICU admission;<br>4. With one or more organ failure (SOFA for any individual organ system⩾2);<br>5. Expected to stay in ICU for more than seven days;<br>6. Oral diet is not likely to be restored within three days.<br>5.22 Exclusion Criteria<br>1. Patients receive EN in the past three days before admission;<br>2. Patients receiving palliative treatment or expected to die within 48 hours;<br>3. Women in pregnancy;<br>4. Long-term use of steroids or immunosuppressive agents;<br>5. Patients with malignant diseases receiving radiotherapy or chemotherapy.	The original eligibility criteria is not specific enough

Page 13, Section 6. Randomization and assignment of interventions First Line	Blank	6.1 Patients screening procedures<br>All patients presenting to the ICUs of each participating hospital will be assessed by the treating physician and receive medical care immediately according to the current best clinical practice. The treating clinician team will be responsible for identifying potential patients and contact the NEED coordinator or a member of the NEED study team who will assess the patients for eligibility into the study. Screening tools will be provided, and a screening log will be kept. Each participating center will be led by a NEED project leader (center primary investigator) and a site coordinator. The former will be responsible for implementing the feeding guideline if assigned to the study group and monitor the NEED trial. The latter will be responsible for daily screening and data management.<br>6.2 Recruitment<br>All patients admitted to the participating ICUs will be considered for enrollment and assessed for inclusion and exclusion criteria. Where possible, informed consent will be obtained from the patient or next of kin (NOK) prior to enrollment into the study.	Add new section parts
Page 13, Section 7. Outcome measures First Line	7.1 Primary outcome measures<br>28-day mortality<br>7.2 Secondary outcome measures<br>1.Duration of mechanical ventilation at 28 days;<br>2.New nosocomial infection;<br>3.Proportion of patients receive nutrition therapy (EN/PN);<br>4.Mean energy delivery of EN;<br>5.Mean energy delivery of PN;<br>6.Time to start nutrition therapy (EN/PN);<br>7.EN tolerance score;<br>8.Days requiring prokinetic agents;<br>9.New-onset organ failure;	7.1 Primary outcome measures<br>The primary outcome measure is all-cause mortality at day 28 after enrollment (the day of enrollment will be set as day 1).<br>7.2 Secondary outcome measures<br>1. Time to start EN<br>2. Time to start PN<br>3. Mean nutrition support days within first seven days after enrollment<br>4. Mean energy per day over the first seven days for patients who were fed<br>5. The proportion of patients never fed during the first seven days after enrollment<br>6. The proportion of patients received EN within two days after enrollment<br>7. The proportion of patients received PN within two days after	To make the endpoints more specific

		enrollment<br>8. The proportion of fed patients within two days after enrollment<br>9. The proportion of patients received EN or PN within two days after enrollment<br>10. EN tolerance score during the first seven days after enrollment<br>11. Days requiring prokinetic agents within the first seven days after enrollment<br>12. The proportion of patients who received a post-pyloric feeding tube (patients receiving EN) within first seven days after enrollment.<br>13. New-onset organ failure within first seven days (respiratory, cardiovascular, renal)<br>14. Days requiring MV within first seven days after enrollment.<br>15. Incidence of new infection in ICU	

Protocol Amendment 2 (version 3, 13 May 2018)

Page/ Line No.	Original text	New text	Reason
Page 35, Section 2.3 Writing and publication committee Line 8	Prof. Weiqin Li<br>Dr. Lu Ke<br>Dr. Zhongheng Zhang<br>Ms. Jiajia Lin	Prof. Weiqin Li<br>Prof. Lu Ke<br>Dr. Yang Wang<br>Dr. Zhongheng Zhang<br>Prof. Gordon Doig<br>Prof. Arthur R.H. van Zanten<br>Ms. Jiajia Lin	Add members of the writing and publication committee
Page 42, Section 7.2 Secondary outcome measures First Line	1. Time to start EN<br>2. Time to start PN<br>3. Mean nutrition support days within first seven days after enrollment<br>4. Mean energy per day over the first seven days for patients who were fed<br>5. The proportion of patients never fed during the first seven days after enrollment<br>6. The proportion of patients received EN within two days after enrollment<br>7. The proportion of patients received PN within two days after enrollment<br>8. The proportion of fed patients within two days after enrollment<br>9. The proportion of patients received EN or PN within two days after enrollment<br>10. EN tolerance score during the first seven days after enrollment<br>11. Days requiring prokinetic agents within the first seven days after enrollment	*7.2.1 Process measures*<br>1. Time to start EN<br>2. Time to start PN<br>3. Mean nutrition support days within first seven days after enrollment<br>3.1 Mean nutritional support(either EN or PN or both) days within first seven days after enrollment<br>3.2 Mean EN support days within first seven days after enrollment<br>3.3 Mean PN support days within first s even days after enrollment<br>4. Mean energy per day over the first seven days for patients who were fed<br>4.1 Mean EN per day over the first seven days for patients who were fed<br>4.2 Mean PN per day over the first seven days for patients who were fed<br>5. The proportion of patients never fed during the first seven days after enrollment<br>6. The proportion of patients received EN within two days after enrollment<br>7. The proportion of patients received PN within two days after enrollment<br>8. The proportion of fed patients within two days after enrollment<br>9. The proportion of patients received EN or PN within two days after enrollment<br>10. EN tolerance score during the first seven days after enrollment<br>11. Days requiring prokinetic agents within the first seven days after enrollment<br>12. The proportion of patients who received a post-pyloric feeding tube (patients receiving EN) within first seven days after enrollment.<br>*7.2.2 Organ dysfunction-related outcomes*	To make the endpoints more specific and practical

	12. The proportion of patients who received a post-pyloric feeding tube (patients receiving EN) within first seven days after enrollment.<br>13. New-onset organ failure within first seven days (respiratory, cardiovascular, renal)<br>14. Days requiring MV within first seven days after enrollment.<br>15. Incidence of new infection in ICU	1.New-onset organ failure within first seven days<br>1.1 New-onset respiratory failure;<br>1.2 New-onset cardiovascular failure;<br>1.3 New-onset renal failure<br>2. New receipt of organ support therapy within first seven days<br>2.1 New receipt of mechanical ventilation (non-invasive included)<br>2.2 New receipt of renal replacement therapy<br>2.3 New receipt of vasoactive agents<br>2.4 Days requiring CRRT within first seven days after enrollment<br>2.5 Days requiring insulin within first seven days after enrollment<br>2.6 Days requiring MV within first seven days after enrollment<br>*7.2.3 Additional outcomes*<br>1. Incidence of new infection in ICU<br>2. ICU-free days within 28 days	
Page 42, Section 7.2 Secondary outcome measures Last Line	Blank	7.3 Definition of outcomes<br>*New-onset organ failure:* organ failure occurring during the first seven days and not present at enrollment. Organ failure is defined as an increase in the Sequential Organ Failure Assessment (SOFA) score of 2 points or more for each organ system (Respiration, Cardiovascular, Renal).<br>*New receipt of organ support therapy:* requirement of organ support therapy (mechanical ventilation, renal replacement therapy, and vasoactive agents) not applied at enrollment.<br>*All-cause mortality at day 28:* defined as patient vital status (alive/dead) to be determined on the 28th calendar day post-enrollment (the day of enrollment will be set as day1).<br>*New infection in the ICU:* defined as a new infection appearing at least 48hrs after ICU admission. The diagnosis of infection was made on the basis of medical history, physical examination, and the results of laboratory examinations requiring antimicrobial therapy.<br>*ICU-free days within 28 days:* defined as days alive and free from the need for intensive care) from enrollment to day 28. In patients who admission to the study ICU more than once through day 28, only the final period of ICU stay was included in ICU-free days. Patients who ICU discharged on day 28 or died before day 28 were assigned zero ICU-free days.	Describe in detail

Protocol Amendment 3 (version 4, 02 Jan 2019)

Original text	New text	Reason
Blank	**2.1 Study timelines**<br>December 2015 Feeding guideline drafted<br>April 2016 The first National Expert Symposium<br>May 2016 Feeding guideline and graphic protocol V1 finalized<br>June 2016 Communication with potential sites for research interests<br>May 2017 Hospital Human Research Ethics Committee (HREC) submissions<br>July 2017 HREC approvals obtained<br>August 2017 Commence vanguard phase and training in test sites<br>January 2018 Patient recruitment commences (all sites)<br>February 2019 Interim analysis<br>June 2019 Patient enrollment completed<br>July 2019 Follow-up completed<br>November 2019 Analysis Plan finalized<br>December 2019 Initiation of analysis	Add Study timelines
Blank	8.3 The flow of ICUs and patients<br>The flow of hospitals and patients through the trial will be reported in accordance with the CONSORT extension statement for cluster trials. The flow diagram will include the number of eligible and recruited sites, the number of eligible and recruited patients and then, by allocated group, the number of patients who continued through the trial, the number of withdrawing, the number of data missing, and the numbers included in the analysis.	Describe in detail

EffectiveNess of a feEding protocol on nutritional thErapy and clinical outcomes in critically ill patients: a multi-center, cluster-ranDomized, parallel-controlled trial

NEED

Statisical Analysis Plan

ISRCTN registry: ISRCTN12233792

Corrsponding protocol version and date: Version4.0- 01/Jan/2019

Date: Nov 12, 2019

Primary Investigator

Professor. Weiqin Li

Director of surgical intensive care unit, Jinling Hospital, Nanjing University

305 East Zhongshan Rd,210002

Nanjing, Jiangsu province, China

Phone: +8680860007

Fax:+8680863073

This analysis plan was reviewed and approved by,

Weiqin Li, Professor in Intensive Care Medicine, Jinling Hospital, Nanjing, China.

Lu Ke, Professor in Intensive Care Medicine, Jinling Hospital, Nanjing, China.

Yang Wang, Statistician, Department of Medical Research & Biometrics Center, Chinese Academy of Medical Sciences & Peking Union Medical College, Beijing, China.

Gordon Doig, Associate Professor in Intensive Care Medicine, Northern Clinical School, Royal, North Shore Hospital, University of Sydney, Sydney, Australia

Signed agreement on Statistical Analysis Plan

**Signatures**		
	Signature	Date
(Trial Statistician)	Yang Wang	2019.11.12
(Chief Investigator)	[illegible]	2019.11.12
(DSMB Chair)	Yuxin Liu	2019.11.12

TABLE OF CONTENTS

ABBREVIATIONS
BRIEF BACKGROUND
1 Introduction ... 101
2 Development of the feeding guideline ... 101
3 Hypotheses to be tested ... 102
4 Eligibility criteria ... 102
4.1 Inclusion Criteria ... 102
4.2 Exclusion Criteria ... 103
5 Randomization ... 103
6 Intervention arm ... 103
6.2 Control group ... 104
6.3 General management ... 104
7 Data collection and follow up ... 104
7.1 Data collection ... 104
7.2 Follow up ... 105
8 Outcome measures ... 105
8.1 Primary outcome measures ... 105
8.2 Secondary outcome measures ... 105
8.3 Definition of outcomes ... 106
STATISTICAL ANALYSIS
9 Data and safety monitoring board ... 107
10 Sample size and power ... 107
11 The flow of ICUs and patients ... 108
12 Withdrawals ... 108
13 Basic principles of analysis ... 108
14 Unadjusted analysis and adjusted analyses ... 109
15 Presentation of comparative analyses ... 109
16 Analysis of primary outcome ... 110
17 Analysis of secondary outcomes ... 110
18 Missing primary outcomes ... 111
19 Missing baseline prognostic variables ... 111
REFERENCE

ABBREVIATIONS

SCCM	Society of Critical Care Medicine
ASPEN	American Society for Parenteral and Enteral Nutrition
ESICM	European society of intensive medicine
SOFA	Sequential Organ Failure Assessment
ICU	Intensive Care Unit
AGI	Acute Gastrointestinal Injury
EN	Enteral Nutrition
PN	Parenteral Nutrition
CRRT	Continuous Renal Replacement Therapy
MV	Mechanical Ventilation
DSMB	Data and safety monitoring board
AE	Adverse Event
ICC	Intraclass Correlation Coefficient
ITT	Intention To Treat
FAS	Full-Analysis Set
BMI	Body Mass Index
MAR	Missing At Random

BRIEF BACKGROUND

1 Introduction

China has one of the largest populations of critically ill patients, causing a great burden on the healthcare system, and the clinical practice for nutritional therapy varies massively across China [1-2]. A small study has shown that a feeding protocol could improve the proportion of EN from 32% to 78% at day7 [3]. However, to the best of our knowledge, there is a lack of large-scale data on the practice of enteral nutrition in Chinese ICUs. Our earlier cross-sectional study showed that the proportions of subjects starting EN within 24, 48 and 72 h after ICU entry was 24.8% (84/352), 32.7% (150/459), and 40.0% (200/541), respectively, suggesting the suboptimal implementation of the current guidelines [4]. Therefore we conducted this multicenter cluster-randomized study to assess the effect of a feeding guidelien based on the latest guidelines with educational materials in a group of ICUs. For a protocol study, a cluster-randomized method can effectively control the confounding factors across groups to obtain more reliable conclusions [5]. In addition, patient-level randomization would inevitably lead to pollution effect as the feeding protocol in the study group would always affect the clinical practice in the controls [6]. Randomization at the ICU level and stratification before randomization could help avoid pollution and between-group imbalance.

2 Development of the feeding guideline

According to the Society of Critical Care Medicine (SCCM) and American Society for Parenteral and Enteral Nutrition (ASPEN), European society of intensive medicine (ESICM) clinical practice guidelines and Surviving Sepsis Campaign guidelines, we developed an evidence-based feeding guideline and related educational materials like graphic feeding protocol for critically ill patients and expected to improve patient outcomes with the implementation of the guideline [7-9]. The ACCEPT trial has

demonstrated that evidence-based algorithms for the critically ill patients could improve the nutritional practice, reduce hospital stay, and may decrease the hospital mortality rate [10]. Conversely, two other cluster-randomized trials have indicated that implementation of a feeding protocol could provide more and earlier energy supply, but did not improve clinical outcomes [11-12]. However, controversy remains due to the lack of large-scale adequately-powered randomized controlled trials in the literature. A small pilot study had shown that our feeding protocol was able to increase the proportion of EN feeding [3]. Due to the before-and-after nature of the pilot study and the limited sample size, that study was not powered enough to fully demonstrate the effect of this feeding guideline on clinical outcomes of critically ill patients, but the preliminary data it showed justified a large randomized controlled study.

3 Hypotheses to be tested

We aimed to assess the effect of a feeding guideline on nutritional therapy and clinical outcome in critically ill patients. The hypothesis is that implementation of a feeding guideline, compared to routine clinical practice, could reduce 28-day mortality in critically ill patients admitted to ICUs in China.

4 Eligibility criteria

4.1 Inclusion Criteria

1. Informed consent form obtained from the patient or next of kin;
2. 18 years old or older;
3. Within 24h of ICU admission;
4. With one or more organ failure(SOFA for any single organ system≥2);
5. Expected to stay in ICU for more than seven days;
6. Oral diet is not likely to be restored within three days.

4.2 Exclusion Criteria

1. Patients received EN in the past three days;
2. Patients receiving palliative treatment or expected to die within 48 hours;
3. Women in pregnancy;
4. Long-term use of steroids or immunosuppressive agents;
5. Patients with malignant diseases receiving radiotherapy or chemotherapy.

5 Randomization

The R language was used for randomization in this study. All the participating centers were stratified according to the provincial distribution and type of ICU (emergency, medical, surgical, and others). Randomization occurred in a 1:1 fashion for the participating centers within the same category with computer-generated random numbers.

A web-based electrical database was used for data collection and storage. All data was input by the primary investigator or nominated investigator (two or fewer nominated investigators for each participating center) approved by the primary investigator.

6 Intervention arm

Before EN initiation, hemodynamic parameters should be stabilized evidenced by MAP≥65 mmHg and lactate<4mmol/L, with decreasing vasoactive dose. The gastrointestinal function will then be evaluated with the acute gastrointestinal injury (AGI) grading system. More details for implementation of the feeding protocol could be found in Fig. 1. Patients on EN will be evaluated using a tolerance score for every 4-6 hours. EN will be discontinued when the EN tolerance score is greater than 5 points. Adverse events during EN implementation will be managed with a standardized protocol (Fig. 2) by the treating team.

6.2 Control group

All the participants in this group will be treated without any change to current clinical practice. No adherence to uniform protocol or guidelines will be required for the study period.

6.3 General management

All patients would be cared for by the local treating team in each participating ICU, including monitor of vital signs, harvesting necessary blood samples for laboratory measurement, fluid therapy, etc. In the control group, nutritional therapy would be implemented routinely in each participating ICU. All co-interventions will be left to the discretion of the treating clinical teams and recorded in the patient's medical record.

7 Data collection and follow up

7.1 Data collection

All the data that is necessary to define baseline patient characteristics, the implementation of the feeding protocol and control therapies, potential confounding co-interventions, and outcomes will be collected.

The primary investigator of each center will be responsible for the enrollment of patients and data input. A group of statisticians will be accountable for the pre-definition of statistical analysis and subgroup analysis.

A web-based electrical database will be used for data collection and storage. All data will be input by the primary investigator or nominated investigator(less than two for each participating center) approved by the primary investigator. Training for data entry will be performed by the supplier of the electrical database and the sponsor of the NEED trial.

7.2 Follow up

All patients recruited will be followed until either 28 day after enrollment or death, depending on which comes first. Follow-up will be restricted to information regarding the vital status and other related study clinical outcomes. Follow-up will be conducted by study staff through either direct contact with the patient or their next of kin. Patients who withdraw from the study for any reason will also be followed up according to the study follow-up schedule, unless they also withdrawed consent for data collection. All patient will be analyzed on an intention-to-treat principle.

8 Outcome measures

8.1 Primary outcome measures

The primary outcome measure is all-cause mortality at day 28 after enrollment (the day of enrollment will be set as day 1).

8.2 Secondary outcome measures

8.2.1 Process measures

1. Time to start EN
2. Time to start PN
3. Mean nutrition support days within first seven days after enrollment
   3.1 Mean nutritional support(either EN or PN or both) days within first seven days after Enrollment
   3.2 Mean EN support days within first seven days after enrollment
   3.3 Mean PN support days within first s even days after enrollment
4. Mean energy per day over the first seven days for patients who were fed
   4.1 Mean EN per day over the first seven days for patients who were fed
   4.2 Mean PN per day over the first seven days for patients who were fed
5. The proportion of patients never fed during the first seven days after enrollment
6. The proportion of patients received EN within two days after enrollment
7. The proportion of patients received PN within two days after enrollment

8. The proportion of fed patients within two days after enrollment

9. The proportion of patients received EN or PN within two days after enrollment

10. EN tolerance score during the first seven days after enrollment

11. Days requiring prokinetic agents within the first seven days after enrollment

12. The proportion of patients who received a post-pyloric feeding tube (patients receiving EN) within first seven days after enrollment.

8.2.2 Organ dysfunction-related outcomes

1.New-onset organ failure within first seven days

1.1 New-onset respiratory failure;

1.2 New-onset cardiovascular failure;

1.3 New-onset renal failure

2. New receipt of organ support therapy within first seven days

2.1 New receipt of mechanical ventilation (non-invasive included)

2.2 New receipt of renal replacement therapy

2.3 New receipt of vasoactive agents

2.4 Days requiring CRRT within first seven days after enrollment

2.5 Days requiring insulin within first seven days after enrollment

2.6 Days requiring MV within first seven days after enrollment

8.2.3 Additional outcomes

1. Incidence of new infection in ICU

2. ICU-free days within 28 days

8.3 Definition of outcomes

New-onset organ failure: organ failure occurring during the first seven days and not present at enrollment. Organ failure is defined as an increase in the Sequential Organ Failure Assessment (SOFA) score of 2 points or more for each organ system(Respiration, Cardiovascular, Renal).

New receipt of organ support therapy: requirement of organ support therapy (mechanical ventilation, renal replacement therapy, and vasoactive agents) not applied at enrollment.

All-cause mortality at day 28: defined as patient vital status (alive/dead) to be determined on the 28th calendar day post-enrollment (the day of enrollment will be set as day1).

New infection in the ICU: defined as a new infection appearing at least 48

hrs after ICU admission. The diagnosis of infection was made on the basis of medical history, physical examination, and the results of laboratory examinations requiring antimicrobial therapy.

ICU-free days within 28 days: defined as days alive and free from the need for intensive care) from enrollment to day 28. In patients who admission to the study ICU more than once through day 28, only the final period of ICU stay was included in ICU-free days. Patients who ICU discharged on day 28 or died before day 28 were assigned zero ICU-free days.

STATISTICAL ANALYSIS

9 Data and safety monitoring board

Data and safety monitoring board (DSMB) was composed of an independent group of experts that offered advice during the implementation of the study. The DSMB can recommend that a trial should be stopped early because of concerns about participant safety. Safety variables will be mainly analysis in descriptive way using the number of AEs, the number (%) of participants with AEs by two arms.

10 Sample size and power

According to previous studies, 28-day mortality for mixed ICU patients was demonstrated to be 20%, which was used as the event rate in the control group. The Nutrition protocol was assumed to be able to reduce the mortality rate to at least 12% [10]. The type I error was 0.05, and the statistical power was 80%. The inter-class correlation was 0.1. A total of 90 centers with 2250 subjects were required,which corresponds to a 2250 paticipants.

The sample size calculation was performed by using the CRTSize in R. The full code for the calculation was: n4props (pe=0.12, pc=0.20, m=25, ICC=0.10, AR=1, alpha=0.05, power=0.80)

11 The flow of ICUs and patients

The flow of ICUs and patients through the trial will be reported in accordance with the CONSORT extension statement for cluster trials. The flow diagram will include the number of eligible and recruited centers, the number of eligible and recruited patients, and then, by allocated group, the number of patients who continued through the trial, the number of withdrawing, the number of data missing and the numbers included in the analysis.

12 Withdrawals

In ICUs allocated to the intervention group, patients or their next of kin could choose to opt-out from participating in the collection of outcome measures, while remaining under the intervention if appropriate. Patients who discontinued completing the data collection prior to the end of the trial period will still be included in the full analysis population unless they requested otherwise. Reasons for withdrawal should be documented wherever possible.

13 Basic principles of analysis

The reporting and presentation of this trial will be following the CONSORT guidelines for cluster-randomized trials [13], with the primary comparative analysis being conducted on an intention-to-treat basis.

Descriptive statistics will be used to assess any marked baseline differences in demographics or outcome measures between the two groups, taking clustering into account. Comparisons of binary outcomes will be expressed as risk differences with 95% confidence intervals and comparisons of continuous outcomes as mean differences together with 95% confidence intervals. Between-group comparisons will be made using a generalized linear mixed-model (weighed by clusters).

All analyses will account for clustering to ensure correct type I error rates and confidence intervals. Our cluster-randomized trial will be analyzed on the individual level, and account for clustering among patients in the same cluster.

Based on the principle of intention to treat (ITT), full-analysis set (FAS) will be performed on all the randomized population. FAS will be used for the analysis of baseline characteristics and primary therapeutic interventions

Two-sided 5% significance levels will be used to identify statistically significant results. A two-sided 10% significance level will be used to identify results that are trending towards statistical significance. All confidence intervals reported will be 95% confidence intervals. All p-values and estimates of change will be calculated at the individual level.

14 Unadjusted analysis and adjusted analyses

All comparative analyses will allow for the clustered nature of the data to ensure correct confidence intervals and type I error rates are calculated [14-16]. As the trial includes a reasonable number of clusters (more than 90 ICUs), the analyses will be based on the individual patient-level data, allowing for the clustering between patients within the same ICU, rather than on the cluster-level summarized data, which is appropriate when only a small number of clusters are present [14]. For each outcome, unless otherwise specified, the primary analysis will be the unadjusted analysis taking effects of clustering into account.

Baseline imbalances in potentially confounding variables (P<0.10) will be selected for inclusion in the additional covariate-adjusted analysis, with the statistical models including the two stratification variables (ICU type and provincial distribution) and baseline values for the outcomes under consideration. The results of the adjusted analysis will also be presented for completeness.

15 Presentation of comparative analyses

For each of the continuous outcome variables, the mean and standard deviation for each allocated group will be presented, together with the mean between-group difference, 95% confidence interval for the difference and p value. For binary outcomes, the percentage and frequency of patients in the outcome category of interest (e.g., percentage of new infection) will be presented for each allocated group, along with the relative differences for the intervention effect, 95% confidence

intervals for the relative differences and p value. Besides, the intra-cluster correlation coefficient will be reported for each outcome based on the unadjusted analyses, together with a 95% confidence interval.

16 Analysis of primary outcome

The primary conclusions of the NEED trial will be based on analyses conducted under the principle of intention to treat (ITT). Comparisons between the two groups will be implemented using a generalized linear mixed-model, allowing for the clustered nature of the data (see ‘Unadjusted and adjusted analyses’ section above).

17 Analysis of secondary outcomes

We consider all secondary analyses to be exploratory and hypothesis-generating and, therefore, do not adjust for multiple comparisons. Secondary outcomes based on count data were analyzed using a Poisson model. Baseline balance of proportions will be assessed using a Chi-square test, and continuous variables are assessed using t-tests. All analyses will be appropriately adjusted for the effects of clustering.

The following are baseline variables ascertained at the time of enrollment: Age, Gender, Weight, BMI (as a continuous variable *and* categorized into underweight [BMI $< 18.5 kg/m^2$] and obese [BMI ≥ 30 kg/m^2]), APACHE II score, NUTRIC score, SOFA score, proportion of infectious patients within first 7 days, confirmed infection site (Pulmonary, Urinary tract, Abdominal cavity, Blood, Catheter, Intracranial, Others), Source of admission (Emergency department, Surgical department, Medical department, Other hospital, others), Surgical admission type (Elective, Emergency), Comorbidities (Hypertension, Coronary disease, Insulin dependent Type I or II diabetes, Chronic Respiratory diseases, Stroke, Gastrointestinal disease, Malignant tumor, others), Admission diagnosis (Cardiovascular, Respiratory, Trauma, Post-CPR, Neurological, End-stage disease, Metabolic, Perioperative, Sepsis, others), current status of organ failure (respiration, renal and cardiovascular described by individual SOFA score), current therapy for sedation and analgesia(Ramsay score), current status of infection (site, culture results and date of diagnosis) and gastrointestinal function (described by AGI score).

18 Missing primary outcomes

Missing primary outcomes (Day 28 vital status) will be assumed to be missing at random (MAR) and thus will be ‘ignored’ in the primary analysis. However, if greater than 5% of all primary outcomes that should be available for analysis are missing, a sensitivity analysis will be undertaken in addition to the primary MAR analysis.

19 Missing baseline prognostic variables

Missing baseline prognostic variables will be replaced with mean values calculated from the observed non-missing instances of that baseline prognostic variable. The imputed means will be calculated using pooled data from both arms. Imputed means will *not* be calculated within the treatment arm using treatment arm-specific data *nor* will any post-enrollment information be incorporated into the calculation. Furthermore, replacement values for missing calculated constructs such as BMI and APACHE II score will be estimated using non-missing component-level information. For example, if one of the components of BMI is missing, such as height, overall mean height will be imputed, and BMI will be calculated with the known weight and imputed mean height.

If a baseline prognostic variable requires an imputation of missing values, the percent of cases that were originally missing will be reported.

REFERENCE

1.Liu, P.; Jiang, L.; Li, C.; Sun, M.; Rieger, A.; Hao, M., How to deal with burden of critical illness: A comparison of strategies in different areas of China. *Pak J Med Sci* **2014,** *30* (3), 659-63.

2.Adhikari, N. K.; Fowler, R. A.; Bhagwanjee, S.; Rubenfeld, G. D., Critical care and the global burden of critical illness in adults. *Lancet* **2010,** *376* (9749), 1339-46.

3.Li, Q.; Zhang, Z.; Xie, B.; Ji, X.; Lu, J.; Jiang, R.; Lei, S.; Mao, S.; Ying, L.; Lu, D.; Si, X.; Ji, M.; He, J.; Chen, M.; Zheng, W.; Wang, J.; Huang, J.; Wang, J.; Ji, Y.; Chen, G.; Zhu, J.; Shao, Y.; Lin, R.; Zhang, C.; Zhang, W.; Luo, J.; Lou, T.; He, X.; Chen, K.; Peng, W.; Sun, R., Effectiveness of enteral feeding protocol on clinical outcomes in critically ill patients: A before and after study. *PLoS One* **2017,** *12* (8), e0182393.

4.Xing, J.; Zhang, Z.; Ke, L.; Zhou, J.; Qin, B.; Liang, H.; Chen, X.; Liu, W.; Liu, Z.; Ai, Y.; Wang, D.; Wang, Q.; Zhou, Q.; Zhang, F.; Qian, K.; Jiang, D.; Zang, B.; Li, Y.; Huang, X.; Qu, Y.; Xie, Y.; Xu, D.; Zou, Z.; Zheng, X.; Liu, J.; Guo, F.; Liang, Y.; Sun, Q.; Gao, H.; Liu, Y.; Chang, P.; Ceng, A.; Yang, R.; Yao, G.; Sun, Y.; Wang, X.; Zhang, Y.; Wen, Y.; Yu, J.; Sun, R.; Li, Z.; Yuan, S.; Song, Y.; Gao, P.; Liu, H.; Zhang, Z.; Wu, Y.; Ma, B.; Guo, Q.; Shan, F.; Yang, M.; Li, H.; Li, Y.; Lu, W.; Wang, L.; Qian, C.; Wang, Z.; Lin, J.; Zhang, R.; Wan, P.; Peng, Z.; Gong, Y.; Huang, L.; Wu, G.; Sun, J.; Deng, Y.; Shi, D.; Zhou, L.; Zhou, F.; Shi, Q.; Guo, X.; Liu, X.; Wu, W.; Meng, X.; Li, L.; Chen, W.; Li, S.; Wan, X.; Chao, Z.; Zhang, A.; Gu, L.; Chen, W.; Wu, J.; Zhou, L.; Zhang, Z.; Weng, Y.; Feng, Y.; Yang, C.; Feng, Y.; Zhao, S.; Tong, F.; Hao, D.; Han, H.; Fu, B.; Gong, C.; Li, Z.; Hu, K.; Kou, Q.; Zhang, H.; Liu, J.; Fan, C.; Zhou, X.; Chen, X.; Sun, J.; Zhou, X.; Song, B.; Sun, C.; Zhao, L.; Dong, X.; Zhang, L.; Tong, D.; Pan, Z.; Cai, C.; Wang, D.; Dong, Y.; Gong, Y.; Wu, Z.; Meng, X.; Wang, P.; Li, W., Enteral nutrition feeding in Chinese intensive care units: a cross-sectional study involving 116 hospitals. *Crit Care* **2018,** *22* (1), 229.

5.Campbell, M. K.; Mollison, J.; Steen, N.; Grimshaw, J. M.; Eccles, M., Analysis of cluster randomized trials in primary care: a practical approach. *Fam Pract* **2000,** *17* (2), 192-6.

6.Wears, R. L., Advanced statistics: statistical methods for analyzing cluster and cluster-randomized data. *Acad Emerg Med* **2002,** *9* (4), 330-41.

7.Taylor, B. E.; McClave, S. A.; Martindale, R. G.; Warren, M. M.; Johnson, D. R.; Braunschweig, C.; McCarthy, M. S.; Davanos, E.; Rice, T. W.; Cresci, G. A.; Gervasio, J. M.; Sacks, G. S.; Roberts, P. R.; Compher, C.; Society of Critical Care, M.; American Society of, P.; Enteral, N., Guidelines for the

Provision and Assessment of Nutrition Support Therapy in the Adult Critically Ill Patient: Society of Critical Care Medicine (SCCM) and American Society for Parenteral and Enteral Nutrition (ASPEN). *Crit Care Med* **2016,** *44* (2), 390-438.

8.Reintam Blaser, A.; Starkopf, J.; Alhazzani, W.; Berger, M. M.; Casaer, M. P.; Deane, A. M.; Fruhwald, S.; Hiesmayr, M.; Ichai, C.; Jakob, S. M.; Loudet, C. I.; Malbrain, M. L.; Montejo Gonzalez, J. C.; Paugam-Burtz, C.; Poeze, M.; Preiser, J. C.; Singer, P.; van Zanten, A. R.; De Waele, J.; Wendon, J.; Wernerman, J.; Whitehouse, T.; Wilmer, A.; Oudemans-van Straaten, H. M.; Function, E. W. G. o. G., Early enteral nutrition in critically ill patients: ESICM clinical practice guidelines. *Intensive Care Med* **2017,** *43* (3), 380-398.

9.Rhodes, A.; Evans, L. E.; Alhazzani, W.; Levy, M. M.; Antonelli, M.; Ferrer, R.; Kumar, A.; Sevransky, J. E.; Sprung, C. L.; Nunnally, M. E.; Rochwerg, B.; Rubenfeld, G. D.; Angus, D. C.; Annane, D.; Beale, R. J.; Bellinghan, G. J.; Bernard, G. R.; Chiche, J. D.; Coopersmith, C.; De Backer, D. P.; French, C. J.; Fujishima, S.; Gerlach, H.; Hidalgo, J. L.; Hollenberg, S. M.; Jones, A. E.; Karnad, D. R.; Kleinpell, R. M.; Koh, Y.; Lisboa, T. C.; Machado, F. R.; Marini, J. J.; Marshall, J. C.; Mazuski, J. E.; McIntyre, L. A.; McLean, A. S.; Mehta, S.; Moreno, R. P.; Myburgh, J.; Navalesi, P.; Nishida, O.; Osborn, T. M.; Perner, A.; Plunkett, C. M.; Ranieri, M.; Schorr, C. A.; Seckel, M. A.; Seymour, C. W.; Shieh, L.; Shukri, K. A.; Simpson, S. Q.; Singer, M.; Thompson, B. T.; Townsend, S. R.; Van der Poll, T.; Vincent, J. L.; Wiersinga, W. J.; Zimmerman, J. L.; Dellinger, R. P., Surviving Sepsis Campaign: International Guidelines for Management of Sepsis and Septic Shock: 2016. *Intensive Care Med* **2017,** *43* (3), 304-377.

10.Martin, C. M.; Doig, G. S.; Heyland, D. K.; Morrison, T.; Sibbald, W. J.; Southwestern Ontario Critical Care Research, N., Multicentre, cluster-randomized clinical trial of algorithms for critical-care enteral and parenteral therapy (ACCEPT). *CMAJ* **2004,** *170* (2), 197-204.

11.Doig, G. S.; Simpson, F.; Finfer, S.; Delaney, A.; Davies, A. R.; Mitchell, I.; Dobb, G.; Nutrition Guidelines Investigators of the, A. C. T. G., Effect of evidence-based feeding guidelines on mortality of critically ill adults: a cluster randomized controlled trial. *JAMA* **2008,** *300* (23), 2731-41.

12.Heyland, D. K.; Murch, L.; Cahill, N.; McCall, M.; Muscedere, J.; Stelfox, H. T.; Bray, T.; Tanguay, T.; Jiang, X.; Day, A. G., Enhanced protein-energy provision via the enteral route feeding protocol in critically ill patients: results of a cluster randomized trial. *Crit Care Med* **2013,** *41* (12), 2743-53.

13.Hemming, K.; Taljaard, M.; McKenzie, J. E.; Hooper, R.; Copas, A.; Thompson, J. A.; Dixon-Woods, M.; Aldcroft, A.; Doussau, A.; Grayling, M.; Kristunas, C.; Goldstein, C. E.; Campbell, M. K.; Girling, A.; Eldridge, S.; Campbell, M. J.; Lilford, R. J.; Weijer, C.; Forbes, A. B.; Grimshaw, J. M., Reporting of stepped wedge cluster randomised trials: extension of the CONSORT 2010 statement with explanation and elaboration. *BMJ* **2018,** *363*, k1614.

14.Flynn, T. N., Design and Analysis of Cluster Randomization Trials in Health Research.: Allan Donner and Neil Klar. London: Arnold, 2000, pp.178, £35.00. ISBN: 0-340-69153-0. *International Journal of Epidemiology* **2001,** *30* (2), 407-408.

15.Kahan, B. C.; Morris, T. P., Assessing potential sources of clustering in individually randomised trials. *BMC Med Res Methodol* **2013,** *13*, 58.

16.Hemming, K.; Eldridge, S.; Forbes, G.; Weijer, C.; Taljaard, M., How to design efficient cluster randomised trials. *BMJ* **2017,** *358*, j3064.

Fig. 1 Graphic Feeding Protocol

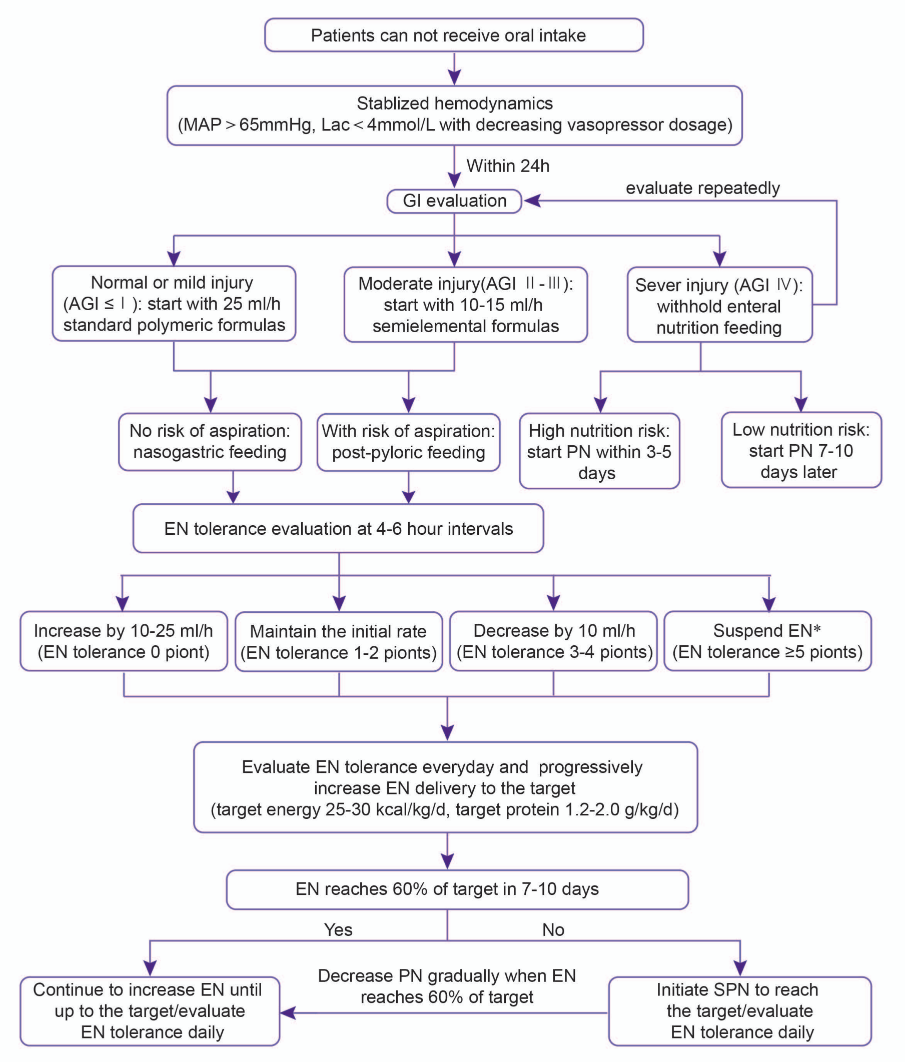

Fig. 2 Protocols for the management of adverse events during feeding

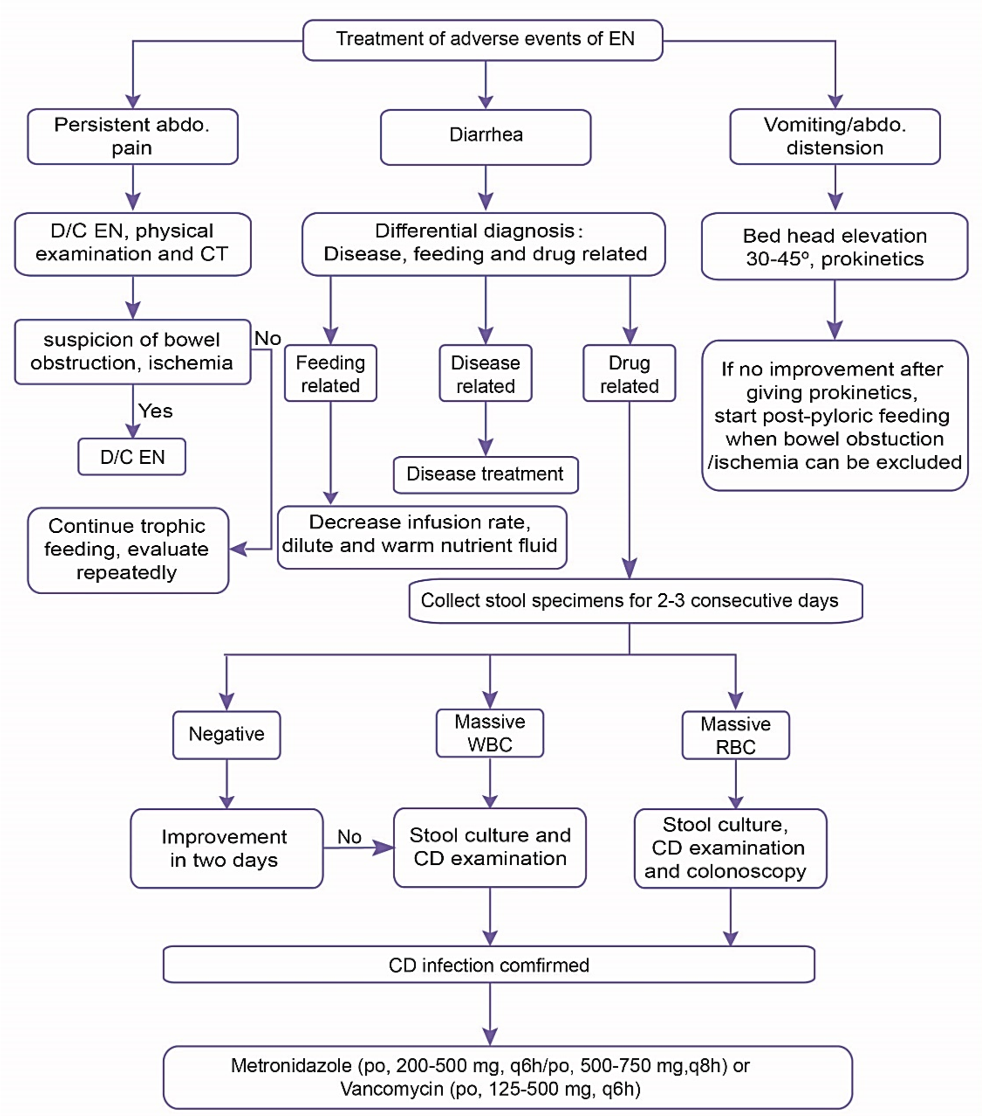

Table 1. EN intolerance score

Points	0	1	2	5
Abdominal distention/pain	None	Mild distension and no abdominal pain	Moderate distension **OR** IAP 15~20mmHg **OR** transient abdominal pain	Severe distension **OR** IAP>20mmHg **OR** persistent abdominal pain
Nausea/vomiting	None	Nausea but no vomiting	Nausea and vomiting without a requirement for decompression **OR** 250ml≦GRV<500ml	Vomiting requiring gastric decompression **OR** GRV≧500ml
Diarrhea	None	Loose stools≧3 times/day with 250≦volume<500ml	Loose stools≧3 times/day with 500≦volume<1500ml	Loose stools≧3 times/day with volume≧1500ml

Total score= Abdominal distension/pain + Nausea/vomiting + Diarrhea

0-2 points: continue enteral nutrition, increase or maintain initial speed, symptomatic treatment;

3-4 points: continue enteral nutrition, slow down the speed, reevaluate EN tolerance after 2h;

≥ 5 points: suspend enteral nutrition, reevaluate or replace the infusion route;
