# Supplementary material for: Actively implementing an evidence-based feeding guideline for critically ill patients (NEED): a multicenter, cluster-randomized, controlled trial
Source: Crit Care. 2022 Feb 16;26:46. doi: 10.1186/s13054-022-03921-5 (PMC8848648; doi:10.1186/s13054-022-03921-5)
Supplement: Supplementary file 2 — Additional file 2. Table S1. Feeding intolerance score. Table S2. Recruitment of patients. Table S3. Proportion of patients receiving enteral and/or parenteral nutrition. Table S4. Proportion of patients receiving enteral nutrition. Table S5. Proportion of patients receiving parenteral nutrition. Table S6. Proportion of enteral nutrition delivered energy in daily energy delivery. Table S7. Target-reaching rate in fed patients from day1 to day7 after enrollment. Table S8. Protein intake from day1 to day7 after enrollment. Table S9. Organ failure-related outcomes. Figure S1. Proportion of enteral nutrition delivered energy in daily energy delivery within the first seven days after enrollment. Figure S2. Target-reaching (more than 70% of the estimated energy target) rate in fed patients for energy delivery from day1 to day7 after enrollment. Figure S3. Daily protein intake from day1 to day7 after enrollment. [file 13054_2022_3921_MOESM2_ESM.docx]

**Electronic supplementary material**

**Actively Implementing an Evidence-based Feeding Guideline for Critically Ill Patients (NEED): A Multicenter, Cluster-randomized, Controlled Trial**

**The NEED Study Investigators:** Lu Ke, Jiajia Lin, Gordon S. Doig, Arthur R H van Zanten, Yang Wang, Juan Xing, Zhongheng Zhang, Tao Chen, Lixin Zhou, Dongpo Jiang, Qindong Shi, Jiandong Lin, Jun Liu, Aibin Cheng, Yafeng Liang, Peiyang Gao, Junli Sun, Wenming Liu, Zhenyu Yang, Rumin Zhang, Wei Xing, An Zhang, Zhigang Zhou, Tingfa Zhou, Yang Liu, Fei Tong, Qiuhui Wang, Aijun Pan, Xiaobo Huang, Chuming Fan, Weihua Lu, Dongwu Shi, Lei Wang, Wei Li, Liming Gu, Yingguang Xie, Rongqing Sun, Feng Guo, Lin Han, Lihua Zhou, Xiangde Zheng, Feng Shan, Jianbo Liu, Yuhang Ai, Yan Qu, Liandi Li, Hailing Li, Zhiguo Pan, Donglin Xu, Zhiqiang Zou, Yan Gao, Chunli Yang, Qiuye Kou, Xijing Zhang, Jinglan Wu, Chuanyun Qian, Weixing Zhang, Minjie Zhang, Yuan Zong, Bingyu Qin, Fusen Zhang, Zhe Zhai, Yun Sun, Ping Chang, Bo Yu, Min Yu, Shiying Yuan, Yijun Deng, Liyun Zhao, Bin Zang, Yuanfei Li, Fachun Zhou, Xiaomei Chen, Min Shao, Weidong Wu, Ming Wu, Zhaohui Zhang, Yimin Li, Qiang Guo, Zhiyong Wang, Yuanqi Gong, Yunlin Song, Kejian Qian, Yongjian Feng, Baocai Fu, Xueyan Liu, Zhiping Li, Chuanyong Gong, Cheng Sun, Jian Yu, Zhongzhi Tang, Linxi Huang, Biao Ma, Zhijie He, Qingshan Zhou, Rongguo Yu, Zhihui Tong, Weiqin Li for the Chinese Critical Care Nutrition Trials Group (CCCNTG)

Contents

**Tables**

[Table S1. Feeding intolerance score. 3](#_Toc93002315)

[Table S2. Recruitment of patients 4](#_Toc93002316)

[Table S3. Proportion of patients receiving enteral and/or parenteral nutrition 8](#_Toc93002317)

[Table S4. Proportion of patients receiving enteral nutrition 9](#_Toc93002318)

[Table S5. Proportion of patients receiving parenteral nutrition 10](#_Toc93002319)

[Table S6. Proportion of enteral nutrition delivered energy in daily energy delivery 11](#_Toc93002320)

[Table S7. Target-reaching rate in fed patients from day1 to day7 after enrollment 12](#_Toc93002321)

[Table S8. Protein intake from day1 to day7 after enrollment 13](#_Toc93002322)

[Table S9. Organ failure-related outcomes 14](#_Toc93002323)

**Figures**

[Figure S1. Proportion of enteral nutrition delivered energy in daily energy delivery within the first seven days after enrollment 15](#_Toc93002324)

[Figure S2. Target-reaching (more than 70% of the estimated energy target) rate in fed patients for energy delivery from day1 to day7 after enrollment 16](#_Toc93002325)

[Figure S3. Daily protein intake from day1 to day7 after enrollment 17](#_Toc93002326)

# Table S1. Feeding intolerance score.

| Points | 0 | 1 | 2 | 5 |
| --- | --- | --- | --- | --- |
| Abdominal distention/pain | None | Mild distension and no abdominal pain | Moderate distension **OR** IAP 15~20mmHg **OR** transient abdominal pain | Severe distension **OR** IAP>20mmHg **OR** persistent abdominal pain |
| Nausea/vomiting | None | Nausea but no vomiting | Nausea and vomiting without a requirement for decompression **OR** 250ml≦GRV<500ml | Vomiting requiring gastric decompression **OR** GRV≧500ml |
| Diarrhea | None | Loose stools≧3 times/day with 250≦volume<500ml | Loose stools≧3 times/day with 500≦volume<1500ml | Loose stools≧3 times/day with volume≧1500ml |

Total score= Abdominal distension/pain + Nausea/vomiting + Diarrhea

0-2 points: continue enteral nutrition, increase or maintain initial speed, symptomatic treatment;

3-4 points: continue enteral nutrition, slow down the speed, reevaluate EN tolerance after 2h;

≥ 5 points: suspend enteral nutrition, reevaluate or replace the infusion route;

# Table S2. Recruitment of patients

| **Participant center** | **ICU type** | **Pts** |
| --- | --- | --- |
| Anhui Provincial Hospital | General ICU | 38 |
| Yijishan Hospital of Wannan Medical College | General ICU | 37 |
| Anhui Medical University Second Affiliated Hospital | General ICU | 28 |
| The First Affiliated Hospital of Anhui Medical University | General ICU | 26 |
| First Affiliated Hospital of Fujian Medical University | General ICU | 43 |
| The People’s Hospital of Fujian Province | General ICU | 37 |
| Union Hospital of Fujian Medical University | General ICU | 33 |
| Fujian Provincial Hospital | Surgical ICU | 1 |
| First People's Hospital of Foshan | General ICU | 73 |
| General Hospital of Southern Theatre Command | Emergency ICU | 35 |
| Guangzhou First People's Hospital | General ICU | 35 |
| The Sixth Affiliated Hospital, Sun Yat-Sen University | General ICU | 31 |
| Huazhong University of Science and Technology Union Shenzhen Hospital | General ICU | 31 |
| Peking University Shenzhen Hospital | General ICU | 30 |
| General ICU, Jinan University First Affiliated Hospital | Surgical ICU | 30 |
| Southern Medical University Zhujiang Hospital | General ICU | 28 |
| Guangdong Second Traditional Chinese Medicine Hospital | General ICU | 27 |
| The Second People’s Hospital of Shenzhen | Medical ICU | 26 |
| First Affiliated Hospital of Guangzhou Medical University | General ICU | 25 |
| Jinan University First Affiliated Hospital | Neurosurgical ICU | 25 |
| Shenzhen People's Hospital | General ICU | 25 |
| Guangdong Provincial People's Hospital | General ICU | 12 |
| Shantou University Medical College First Affiliated Hospital | General ICU | 4 |
| Sun Yat-sen Memorial Hospital, Sun Yat-sen University | General ICU | 4 |
| People's Hospital of Guangxi Zhuang Autonomous Region | Medical ICU | 35 |
| North China University of Science and Technology Affiliated Hospital | General ICU | 42 |
| Tangshan Gongren Hospital | General ICU | 38 |
| Hebei Medical University Second Affiliated Hospital | General ICU | 38 |
| Hebei Medical University Third Affiliated Hospital | General ICU | 25 |
| Luoyang Central Hospital Affiliated to Zhengzhou University | General ICU | 41 |
| Zhengzhou University First Affiliated Hospital | General ICU | 36 |
| Henan Provincial People's Hospital | General ICU | 29 |
| The Second Affiliated Hospital of Harbin Medical University | General ICU | 40 |
| The Fourth Hospital of Medical University | General ICU | 28 |
| First People's Hospital of Yichang | General ICU | 28 |
| Union Hospital Affiliated to Tongji Medical College of Huazhong University of Science and Technology | General ICU | 28 |
| Yichang Central People's Hospital | General ICU | 26 |
| Wuhan General Hospital of Guangzhou Military Region | General ICU | 8 |
| Hubei Provincial People's Hospital | General ICU | 3 |
| The Third Xiangya Hospital of Central South University | General ICU | 39 |
| Xiangya Hospital Central South University | General ICU | 35 |
| The Second Xiangya Hospital of Central South University | General ICU | 28 |
| Changsha Central Hospital | General ICU | 26 |
| Hunan Provincial People's Hospital | General ICU | 19 |
| Suzhou Municipal Hospital | General ICU | 43 |
| Changzhou No.2 People's Hospital affiliated to Nanjing Medical University | General ICU | 40 |
| Wuxi People's Hospital | General ICU | 38 |
| Benq Medical Center | General ICU | 38 |
| Jinling Hospital | Surgical ICU | 36 |
| Yancheng First People's Hospital | General ICU | 27 |
| First Affiliated Hospital of Soochow University | Emergency ICU | 25 |
| Jiangxi Provincial People's Hospital | General ICU | 32 |
| Nanchang University Second Affiliated Hospital | General ICU | 25 |
| The First Affiliated Hospital of Nanchang University | General ICU | 25 |
| The General Hospital of Shenyang Military | Emergency ICU | 32 |
| China Medical University Second Affiliated Hospital | General ICU | 27 |
| The Second Hospital of Dalian Medical University | General ICU | 9 |
| Affiliated Hospital of Inner Mongolia Medical College | General ICU | 35 |
| Inner Mongolia People's Hospital | General ICU | 35 |
| Qindao University Medical College Affiliated Yantai Yuhuangding Hospital | General ICU | 42 |
| Zibo Central Hospital | General ICU | 39 |
| Linyi City People Hospital | General ICU | 38 |
| Jining First People's Hospital | General ICU | 36 |
| Qindao University Medical College Affiliated Hospital | General ICU | 35 |
| Qingdao Municipal Hospital Group | General ICU | 35 |
| Qindao University Medical College Affiliated Hospital | General ICU | 35 |
| No.971 hospital of People’s Liberation Army Navy | General ICU | 35 |
| Tai'an City Central Hospital | General ICU | 29 |
| Qilu Hospital, Shandong University | General ICU | 26 |
| Yantai Mountain Hospital | General ICU | 25 |
| Jining Medical College Affiliated Hospital | General ICU | 4 |
| Shanxi Provincial People's Hospital | General ICU | 37 |
| Shanxi Medical University First Affiliated Hospital | General ICU | 37 |
| Shanxi Bethune Hospital | General ICU | 26 |
| First Affiliated Hospital of Xi'an Jiao Tong University | General ICU | 43 |
| Xijing Hospital | General ICU | 31 |
| Shaanxi Provincial People's Hospital | General ICU | 30 |
| Chengdu University of Traditional Chinese Medicine Affiliated Hospital | General ICU | 41 |
| Sichuan Provincial People's Hospital | General ICU | 38 |
| Dazhou Central Hospital | General ICU | 35 |
| Tianjing Hospital of Integration of Chinese and Western Medicine | General ICU | 18 |
| The First Affiliated Hospital of Xinjiang Medical University | General ICU | 25 |
| First People's Hospital of Kunming | General ICU | 39 |
| First People's Hospital of Yunnan | General ICU | 37 |
| People's Hospital of Yuxi City | General ICU | 36 |
| Kuming Medical University First Affiliated Hospital | Emergency ICU | 31 |
| Zhejiang University School of Medicine Sir Run Run Shaw Hospital | General ICU | 36 |
| Daping Hospital, Army Medical University | General ICU | 45 |
| The Second Affiliated Hospital of Chongqing Medical University | General ICU | 39 |
| Chongqing Medical University First Affiliated Hospital | General ICU | 26 |

ICU denotes intensive care unit.

# Table S3. Proportion of patients receiving enteral and/or parenteral nutrition

| Outcome Measure | Feeding guideline  48 ICUs, 1399 pts | Control  49 ICU, 1373 pts | P-value |
| --- | --- | --- | --- |
|  |  |  |  |
| **The proportion of patients receiving enteral and/or parenteral nutrition within first seven days after enrollment, mean (95% CI)** |  |  |  |
| Day 1 | 47.68（38.47, 56.89） | 53.73（45.66, 61.81） | 0.32 |
| Day 2 | 76.86（70.02, 83.69） | 73.11（66.39, 79.83） | 0.43 |
| Day 3 | 87.65（82.99, 92.32） | 84.46（79.74, 89.19） | 0.34 |
| Day 4 | 91.13（87.32, 94.93） | 86.66（81.32, 92.01） | 0.17 |
| Day 5 | 92.84（89.63, 96.05） | 89.11（83.83, 94.39） | 0.23 |
| Day 6 | 92.23（89.43, 95.03） | 88.55（83.21, 93.88） | 0.22 |
| Day 7 | 93.31（91.29, 95.33） | 89.65（86.02, 93.29） | 0.08 |

CI denotes confidence interval.

# Table S4. Proportion of patients receiving enteral nutrition

| Outcome Measure | Feeding guideline  48 ICUs, 1399 pts | Control  49 ICU, 1373 pts | P-value |
| --- | --- | --- | --- |
|  |  |  |  |
| **The proportion of patients receiving enteral nutrition within first seven days after enrollment, mean (95% CI)** |  |  |  |
| Day 1 | 36.14（28.71, 43.57） | 27.94（22.66, 33.22） | 0.07 |
| Day 2 | 67.53（60.49, 74.56） | 50.57（44.10, 57.04） | 0.0006 |
| Day 3 | 80.08（74.70, 85.46） | 67.45（61.50, 73.4） | 0.0021 |
| Day 4 | 84.94（80.31, 89.57） | 71.59（65.32, 77.86） | 0.0009 |
| Day 5 | 87.73（83.60, 91.85） | 76.35（70.06, 82.63） | 0.0031 |
| Day 6 | 88.95（85.39, 92.51） | 77.70（71.58, 83.81） | 0.002 |
| Day 7 | 91.44（89.15, 93.73） | 82.27（77.43, 87.12） | 0.001 |

CI denotes confidence interval.

# Table S5. Proportion of patients receiving parenteral nutrition

| Outcome Measure | Feeding guideline  48 ICUs, 1399 pts | Control  49 ICU, 1373 pts | P-value |
| --- | --- | --- | --- |
|  |  |  |  |
| **The proportion of patients receiving parenteral nutrition within first seven days after enrollment, mean (95% CI)** |  |  |  |
| Day 1 | 14.92（7.03, 22.80） | 32.08（22.48, 41.67） | 0.0066 |
| Day 2 | 18.77（9.94, 27.61） | 36.56（26.65, 46.47） | 0.0084 |
| Day 3 | 19.32（10.75, 27.89） | 37.47（27.76, 47.17） | 0.0059 |
| Day 4 | 19.62（11.54, 27.70） | 37.23（27.65, 46.80） | 0.0058 |
| Day 5 | 18.18（10.54, 25.82） | 35.47（26.26, 44.67） | 0.0046 |
| Day 6 | 17.98（10.61, 25.34） | 33.66（24.66, 42.65） | 0.0081 |
| Day 7 | 16.36（9.99, 22.72） | 32.24（23.40, 41.08） | 0.0043 |

CI denotes confidence interval.

# Table S6. Proportion of enteral nutrition delivered energy in daily energy delivery

| Outcome Measure | Feeding guideline  48 ICUs, 1399 pts | Control  49 ICU, 1373 pts | P-value |
| --- | --- | --- | --- |
|  |  |  |  |
| **The proportion of EN in daily energy delivery within the first seven days after enrollment, mean ± SD** |  |  |  |
| Day 1 | 70.37 ± 42.76 | 41.66 ± 45.44 | 0.0003 |
| Day 2 | 78.77 ± 36.32 | 57.96 ± 44.66 | 0.0009 |
| Day 3 | 82.82 ± 32.58 | 66.47 ± 41.40 | 0.0035 |
| Day 4 | 84.50 ± 30.71 | 70.10 ± 39.80 | 0.0058 |
| Day 5 | 86.89 ± 28.34 | 73.27 ± 38.22 | 0.0032 |
| Day 6 | 87.60 ± 27.14 | 73.67 ± 37.60 | 0.0015 |
| Day 7 | 88.32 ± 26.22 | 76.21 ± 36.19 | 0.0004 |

EN denotes enteral nutrition; SD denotes standard deviation.

# Table S7. Target-reaching rate in fed patients from day1 to day7 after enrollment

| Outcome Measure | Feeding guideline  48 ICUs, 1399 pts | Control  49 ICU, 1373 pts | P-value |
| --- | --- | --- | --- |
|  |  |  |  |
| **The target-reaching^#^ rate in fed patients^*^ from day3 to day7 after enrollment, mean (95% CI)** |  |  |  |
| Day 1 | 26.36 (21.93, 30.79) | 32.65 (28.15, 37.15) | 0.81 |
| Day 2 | 37.01 (32.43, 41.60) | 38.59 (34.32, 42.86) | 0.69 |
| Day 3 | 47.99 (44.01, 51.97) | 47.74 (43.84, 51.65) | 0.96 |
| Day 4 | 57.38 (53.63, 61.13) | 52.74 (49.18, 56.29) | 0.72 |
| Day 5 | 62.54 (58.84, 66.24) | 58.37 (54.84, 61.91) | 0.77 |
| Day 6 | 67.23 (63.64, 70.83) | 61.61 (58.14, 65.07) | 0.82 |
| Day 7 | 68.84 (65.50, 72.18) | 66.47 (63.36, 69.59) | 0.76 |

CI denotes confidence interval.

**^#^** target-reaching denotes more than 70% of the estimated energy target

*fed patients denotes patients who received oral intake, EN or PN, either alone or in combination

# Table S8. Protein intake from day1 to day7 after enrollment

| Outcome Measure | Feeding guideline  48 ICUs, 1399 pts | Control  49 ICU, 1373 pts | P-value |
| --- | --- | --- | --- |
|  |  |  |  |
| **Protein intake from day1 to day7 after enrollment, g/kg, mean ± SD** |  |  |  |
| Day 1 | 0.20 ± 0.29 | 0. 28 ± 0.39 | 0.20 |
| Day 2 | 0.40 ± 0.56 | 0.46 ± 0.64 | 0.42 |
| Day 3 | 0.49 ± 0.36 | 0.54 ± 0.45 | 0.33 |
| Day 4 | 0.56 ± 0.38 | 0.59 ± 0.43 | 0.65 |
| Day 5 | 0.60 ± 0.38 | 0.65 ± 0.44 | 0.43 |
| Day 6 | 0.62 ± 0.42 | 0.65 ± 0.47 | 0.55 |
| Day 7 | 0.64 ± 0.43 | 0.67 ± 0.48 | 0.61 |

SD denotes standard deviation.

# Table S9. Organ failure-related outcomes

| Outcome Measure | Feeding guideline  48 ICUs, 1399 pts | Control  49 ICU, 1373 pts | Difference (95% CI) | P-value |
| --- | --- | --- | --- | --- |
|  |  |  |  |  |
| **New-onset organ failure within first seven days after enrollment, patients/No. (%)** |  |  |  |  |
| New-onset respiratory failure | 178 (13.3%) | 138 (10.3%) | 3.1% [ -0.8%; 6.9%] | 0.11 |
| New-onset cardiovascular failure | 88 (6.6%) | 97 (7.3%) | -1.2% [ -3.8%; 1.4%] | 0.36 |
| New-onset renal failure | 75 (5.6%) | 82 (6.2%) | -1.1% [ -3.0%; 0.7%] | 0.22 |
| **Organ support therapy within first seven days after enrollment, treatment days/10 patient-days** |  |  |  |  |
| Renal replacement therapy | 0.97 ± 2.50 | 1.46 ± 3.05 | -0.48 [ -0.88, -0.08] | 0.02 |
| Vasoactive agents | 2.19 ± 3.39 | 2.98 ± 3.75 | -0.73 [ -1.34, -0.12] | 0.02 |
| Mechanical ventilation^a^ | 7.18 ± 3.85 | 7.28 ± 3.80 | -0.01 [ -0.63, 0.61] | 0.97 |

CI denotes confidence interval.

^a^ non-invasive mechanical ventilation included.

# Figure S1. Proportion of enteral nutrition delivered energy in daily energy delivery within the first seven days after enrollment

**
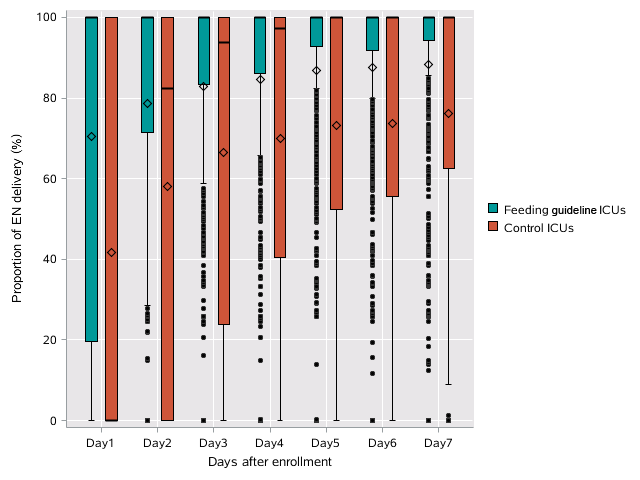
**

EN denotes enteral nutrition. P<0.05 (adjusted for cluster effect) between feeding guidelines and control groups at each day within seven days of enrollment.

# Figure S2. Target-reaching (more than 70% of the estimated energy target) rate in fed patients for energy delivery from day1 to day7 after enrollment

**
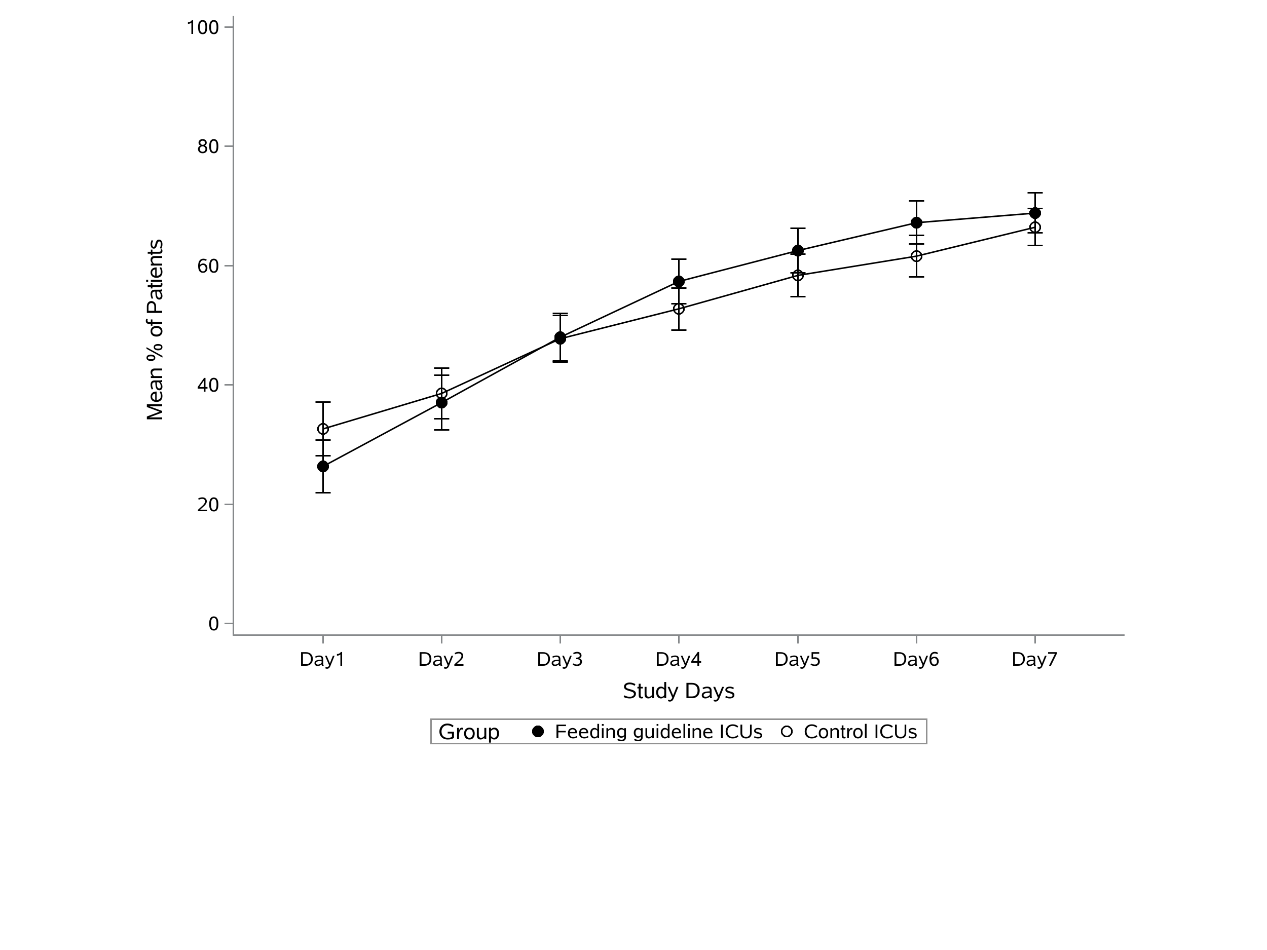
**

*fed patients denotes patients who received oral intake, EN or PN, either alone or in combination.

# Figure S3. Daily protein intake from day1 to day7 after enrollment

**
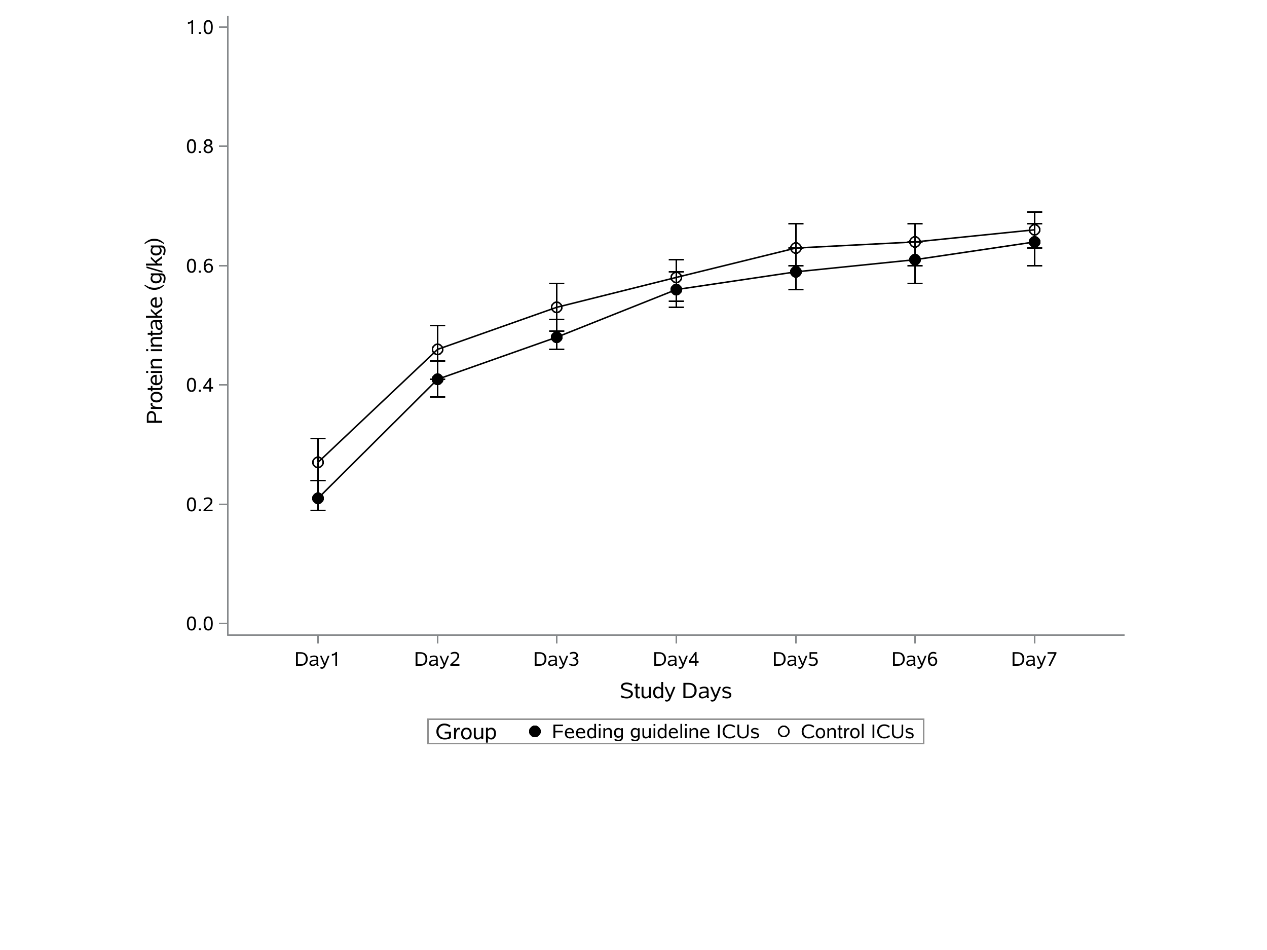
**
