# Supplementary material for: Actively implementing an evidence-based feeding guideline for critically ill patients (NEED): a multicenter, cluster-randomized, controlled trial
Source: Crit Care. 2022 Feb 16;26:46. doi: 10.1186/s13054-022-03921-5 (PMC8848648; doi:10.1186/s13054-022-03921-5)
Supplement: Supplementary file 3 — Additional file 3. Continued Table 1. Baseline ICU and Patient-Level Characteristics.. [file 13054_2022_3921_MOESM3_ESM.docx]

**Continued Table 1**

**Table 1. Baseline ICU and Patient-Level Characteristics**

| Characteristics | Feeding guideline  48 ICUs, 1399 pts | Control  49 ICU, 1373 pts | P-value |
| --- | --- | --- | --- |
| **ICU-level characteristics** |  |  |  |
| Geographical region, No. (%) |  |  | 0.62 |
| Western | 8 (16.7) | 10 (20.4) |  |
| Eastern | 25 (52.1) | 25 (51) |  |
| Central Region | 12 (25) | 12 (24.5) |  |
| Northeast | 3 (6.2) | 2 (4.1) |  |
| **Patient-level characteristics** |  |  |  |
| SOFA score for individual organ systems, mean±SD |  |  |  |
| Respiration | 2.2 ±1.2 | 2.3 ±1.1 | 0.15 |
| Renal | 0.8 ±1.3 | 0.9 ±1.4 | 0.29 |
| Cardiovascular | 1.1 ±1.7 | 1.4 ±1.7 | 0.31 |
| Proportion of infectious patients after enrollment, No. (%) | 1177(84.1%) | 1145(83.4%) | 0.97 |
| Confirmed infection site, No. (%) |  |  |  |
| Pulmonary | 998(71.3%) | 901(65.6%) | 0.23 |
| Urinary tract | 50( 3.6%) | 53( 3.9%) | 0.74 |
| Abdominal cavity | 113(8.1%) | 212(15.4%) | 0.03 |
| Blood | 56(4.0%) | 70( 5.1%) | 0.42 |
| Catheter | 6(0.4%) | 26(1.9%) | 0.21 |
| Intracranial | 74(5.3%) | 73(5.3%) | 0.76 |
| Others | 91(6.5%) | 75(5.5%) | 0.85 |
| Location before ICU admission, No. (%) |  |  | 0.27 |
| Emergency department | 580 (42.6%) | 547 (40.0%) |  |
| Surgical department | 351 (25.8%) | 346 (25.3%) |  |
| Medical department | 235 (17.3%) | 233 (17.0%) |  |
| Other hospital | 175 (12.9%) | 233 (17.0%) |  |
| Others | 19 (1.4%) | 9 (0.7%) |  |
| Admission diagnosis, No. (%) |  |  |  |
| Cardiovascular | 364(26.8%) | 495(36.2%) | 0.11 |
| Respiratory | 808(59.4%) | 838(61.3%) | 0.98 |
| Trauma | 132(9.7%) | 140(10.2%) | 0.30 |
| Post-CPR | 64(4.7%) | 75(5.5%) | 0.43 |
| Neurological | 439(32.3%) | 344(25.1%) | 0.13 |
| End-stage disease | 23(1.7%) | 17(1.2%) | 0.41 |
| Metabolic | 289(21.3%) | 311(22.7%) | 0.96 |
| Perioperative | 183(13.5%) | 199(14.5%) | 0.84 |
| Others | 129(9.5%) | 99(7.2%) | 0.28 |

ICU denotes intensive care unit; SOFA denotes sequential organ failure assessment; CPR denotes cardiopulmonary resuscitation.
